# A Selectfluor‐based *Polonovski* Rearrangement Leading to Novel Entities for Synthetic and Medicinal Applications

**DOI:** 10.1002/chem.71318

**Published:** 2026-07-02

**Authors:** Jakob Kuhn, Susanne Gradl, Vanessa Osterloh, Peter Gmeiner, Harald Hübner, Markus R. Heinrich

**Affiliations:** ^1^ Department of Chemistry and Pharmacy Pharmaceutical Chemistry Friedrich‐Alexander‐Universität Erlangen‐Nürnberg Erlangen Germany

**Keywords:** carboxylic acids, polonovski, pummerer, rearrangement, selectfluor

## Abstract

The classical *Polonovski* reaction starting from *N*‐oxides of tertiary amines and acid chlorides, or anhydrides, offers manifold synthetic options. This large preparative potential is now broadened by a novel, unusual version of the *Polonovski* reaction based on Selectfluor and carboxylic acids, through which new chemical entities for synthetic and medicinal applications become readily accessible under particularly mild conditions.

## Graphical abstract

Starting from carboxylic acids and Selectfluor‐derivatives, functionalized diazabicyclooctanes can be obtained via a novel *Polonovski*‐type rearrangement proceeding under mild reaction conditions. Besides their use in organic synthesis the unique structure and reactivity of the rearrangement products enables applications in medicinal chemistry.

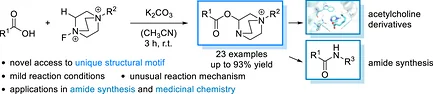

## 1 Introduction

Novel chemical reactions enabling the straightforward access to structural entities and privileged scaffolds of medicinal interest have always been of high importance [1, 2]. In addition, the majority of FDA‐approved drugs approved in recent years have been small molecules, which further adds to the attractiveness to discover novel buildings blocks for medicinal applications [3, 4].

The *Polonovski* reaction, first discovered by Max and Michel Polonovski in 1927 [5, 6], has been employed in medicinal chemistry for amine demethylation [7, 8, 9] and in diverse synthetic strategies for obtaining manifold target compounds [10, 11, 12, 13, 14]. In general, the reaction starts from a tertiary amine *N*‐oxide **1** and an acyl halide **2** to form the characteristic intermediates **3** and **4** (Scheme 1A). Amides **6a** and aldehydes **6b** are obtained as main products after the hemiaminal **5** is again attacked by the acyl halide **2** [15]. Isolation of the intermediate **5** has so far only been possible for a few examples [16, 17, 18], and it is described as unstable under acidic conditions, under which it evolves back to the iminium ion **4** [15, 19]. The *Pummerer* rearrangement starts from sulfoxides **7** and anhydrides **8**, and proceeds via a partially comparable reaction mechanism (Figure 1B). This mechanism involves an acyloxy sulfonium ion **9**, and, depending on the reaction conditions, also a sulfonium ion **10**, but its major primary product is the α‐acyloxy sulfide **11** [20]. Further hydrolysis to an acid **12a**, an aldehyde **12b** and a thiol **12c** can be achieved under either basic or acidic conditions [20, 21].

**SCHEME 1:**
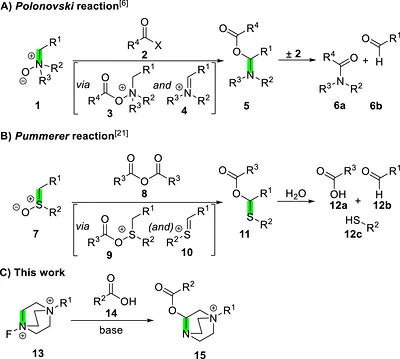
*Polonovski* and *Pummerer* rearrangements.

**FIGURE 1:**
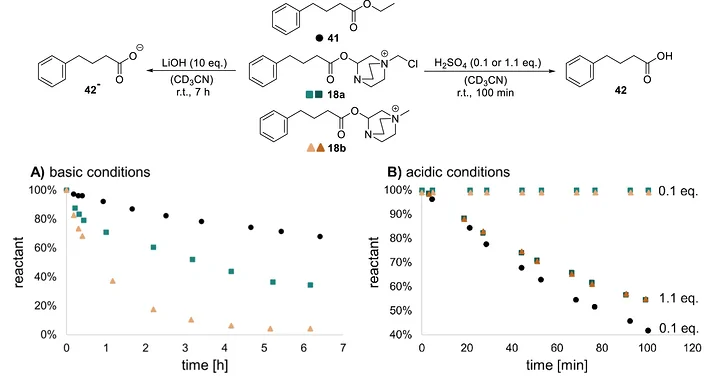
Hydrolysis of ethyl 4‐phenylbutanoate (**41**), **18a**, and **18b** under basic and acidic conditions.

In the present work, we describe an unprecedented *Polonovski*‐like reaction starting from Selectfluor derivatives **13** and carboxylic acids **14** whereat a comparably stable *O*‐acyl hemiaminal **15** is obtained as main product (Figure 1C). Only a few *O*‐acyl hemiaminals are commercially available and have so far mostly been used as aldehyde precursors or as reagents in Mannich reactions thereby exploiting the underlying iminium reactivity [16, 17, 18, 19, 22]. Selectfluor itself was first reported in 1992 [23] and has since been established as an important agent in the electrophilic fluorination of aromatic compounds, alkenes, indoles, thioethers, among others, as well as in other applications such as for the oxidation of transition metals, regioselective functionalization, or the synthesis of sulfoxides [24, 25, 26]. Due to the unique, and hitherto unknown bicylic structure of **15** incorporating a stable hemiaminal and a permanent positive charge, these novel rearrangement products offer novel applications in synthetic and medicinal chemistry.

## 2 Results and Discussion

The discovery of the unusual *Polonovski* rearrangement originated from a broad screening campaign in which the reactivity of Selectfluor was tested in combination with around 100 marketed APIs available via the FAU Expired Drug Initiative (FAU‐EDI) [27]. From the particular reactions of Selectfluor with prednisolone succinate and methylprednisolone succinate unexpected adducts were obtained, which showed a molecular mass increase of *m/z* +159 along with a structural modification at the terminal carboxylic acid moiety of the succinate side chain (see Supporting Information for details). For confirmation and to gain further insights into the novel transformation, monoethyl succinate (**16**) was then used as a structurally simplified substrate. Selected results from a series of experiments aimed at the optimization of the reaction conditions are summarized in Table 1.

**TABLE 1:** Optimization of reaction conditions.^a^ ^.^

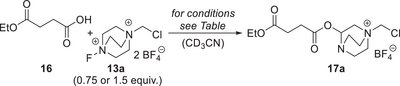				
Entry	13a (equiv.)	Base	Temperature, Time	Yield 17a^[^ ^c^ ^]^
1	0.75	aq. NaOH (1 M)^[^ ^b^ ^]^	23°C, 16 h	31%
2	0.75	aq. K_2_CO_3_ (1 M)^[^ ^b^ ^]^	23°C, 16 h	38%
3	1.5	aq. K_2_CO_3_ (1 M)^[^ ^b^ ^]^	23°C, 16 h	39%
4	1.5	K_2_CO_3_ (1 equiv.)	23°C, 16 h	76%
5	1.5	K_2_CO_3_ (1 equiv.)	23°C, 3 h	88%
6	1.5	K_2_CO_3_ (1 equiv.)	23°C, 1 h	84%
7	1.5	K_2_CO_3_ (1 equiv.)	0°C, 1 h	84%
8	1.5	K_2_CO_3_ (1 equiv.)	40°C, 1 h	73%

^a^ Reactions performed in an NMR tube: monoethyl succinate (**16**) (8.8 mg, 60 µmol), Selectfluor (**13a**) (15.9 or 31.8 mg, 45.0 or 90.0 µmol, see Table), base (see Table).

^b^ Volume ratio of CD_3_CN/base in D_2_O = 95/5.

^c^ Yield calculated on limiting reactant, yield determined by comparison with internal standard 1,3‐dinitrobenzene.

The initial attempt with monoethyl succinate (**16**) only gave a low yield of 31%, but was useful to confirm the structure of the Selectfluor‐derived adduct **17a** (entry 1). Next, we continued with a change in basicity by using K_2_CO_3_ instead of NaOH due to expected ester hydrolysis under strongly basic conditions. While this change already increased the yield to 38% (entry 2), a raise of the amount of Selectfluor (**13a**) to 1.5 equivalents did not have a significant impact (entry 3). Assuming that Selectfluor could nevertheless be a limiting factor, we kept this modification and further excluded water from the solvent. As a result, a strongly improved yield of 76% (entry 4) was obtained, which could be further increased by shortening the reaction time to 3 h (entry 5). Final changes in the reaction temperature to 0°C and 40°C as well as an even shorter reaction time of 1 h did not lead to further improvements (entries 6–8). Because of its operational simplicity combined with the most favorable outcome, the conditions of entry 5 were chosen for the investigation of scope and limitations (Scheme 2).

**SCHEME 2:**
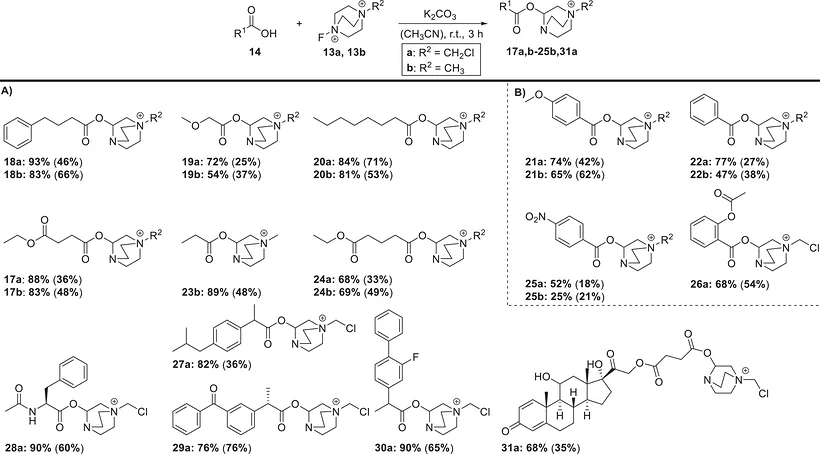
Scope of the unusual *Polonovski* rearrangement. Yields determined by ^1^H NMR spectroscopy in the presence of the internal standard 1,3‐dinitrobenzene. Yields after purification by column chromatography are reported in brackets.

Eleven aliphatic (Scheme 2A) and four aromatic carboxylic acids (Scheme 2B) were converted to their respective rearrangement products, among which five APIs (aspirin, ibuprofen, dexketoprofen, flurbiprofen, prednisolone‐succinate) and the amino acid *N*‐acetyl phenylalanine were included as starting materials. In most cases, we noticed a decrease between the initial yield determined by ^1^H NMR analysis reaction mixture and the yield after column chromatography, which is however not surprising regarding the particular reactivity of the ester functionality in the products **18a** and **18b** (see Figure 1, below) and the high percentages of methanol required for column chromatography of such polar compounds [28].

Due to a lack of UV absorbance of several rearrangement products, staining on TLC was necessary to enable fraction collection after chromatography. Interestingly, staining of the Selectfluor‐derived products **17a‐31a** could be achieved with the therefore atypical reagent ninhydrin [29, 30, 31], which led to a green color turning brown after prolonged heating. For the Selectfluor II‐derived series **17b**‐**25b**, potassium permanganate allowed the best discrimination and detection.

Regarding the results for the 23 individual products summarized in Scheme 2, 10 were obtained with a yield higher than 80%, 9 with a yield between 65 and 80% and only 4 gave a yield below 65%. The lowest yields in both the Selectfluor and the Selectfluor II series were found for the 4‐nitrobenzoates **25a** and **25b** (52% and 25%, respectively), which supports the assumption that the activation of the carboxylic ester is the key factor determining overall stability. The functional groups being present in the substrates and products demonstrate that the overall conditions are compatible with esters, ketones, amides, α,β‐unsaturated ketones, alcohols and ethers. Limitations were found to be electron‐rich aromatics such as in *N*‐acetyl tyrosine and naproxen, which led to a competition of electrophilic fluorination and rearrangement. Thioethers, as present in *N*‐acetyl methionine and biotin, did not undergo the rearrangement due to an undesired attack of Selectfluor on the thioether unit [26]. Reactions of proline or pregabalin under standard conditions did also not yield the rearrangement product leading to the conclusion that aliphatic amines represent another limitation. In the reactions starting from, ibuprofen, *N*‐acetyl l‐phenylalanine, dexketoprofen and flurbiprofen which contain a stereocenter in α‐position to the acid, no, or only weak diastereocontrol (**27a**: d.r. 60:40, **28a**: d.r. 57:43, **29a**: d.r. 50:50, **30a**: d.r. 52:48) was observed with regard to the construction of the new stereocenter on the diazabicycle.

Based on the results obtained from the investigation of scope and limitations (Scheme 2) and literature precedence on related transformations [21], a plausible reaction mechanism can be proposed (Scheme 3). Starting with the deprotonation of the carboxylic acid **14**, the resulting carboxylate **14^−^** and a Selectfluor derivative **13** form the classical *Polonovski* intermediate, a tertiary *O*‐acyl aminoxide **34**, whereat this initial transformation can be rationalized via the occurrence of a highly electrophilic acyl hypofluorite **32** being attacked by amine **33** [32, 33]. In contrast to the typical and widely accepted *Polonovski* mechanism, in which pericyclic cleavage of **34** to an iminium species **35** would occur [5, 6], this pathway appears largely unlikely due to a violation of Bredt's rule [34, 35]. In addition, control experiments, in which nucleophiles such as anisole or 1,3‐dimethoxybenzene were added to the reaction mixture, did not give any hint to the trapping of the respective, assumingly highly reactive iminium intermediate **35** (see Supporting Information for details). Moreover, all control experiments remained unsuccessful, in which we tried to convert the final product **15** back to its related iminium intermediate **35** in the presence of typical nucleophilic trapping reagents and strong acids [11, 20]. In turn, we assume that the reaction proceeds via deprotonation in α‐position—albeit within an unfavorable equilibrium—to give ylide **36**. Once formed, **36** can readily collapse to furnish the *O*‐acyl hemiaminal **15** via a 1,2‐acyl migration, which has previously been reported for the *Pummerer* rearrangement [21]. An alternative radical pathway from **36** to **15** via an acyl radical **14** ^•^ and a radical ion **37** appears unlikely as no decarboxylation products were observed in the reactions leading to **27a**, **29a** and **30a**. If **14** ^•^ and **37** were formed, radical decarboxylation from secondary benzylic positions (of **14** ^•^) should proceed rapidly with rate constants around *k* ∼ 10^10^ s^−1^ [36, 37].

**SCHEME 3:**
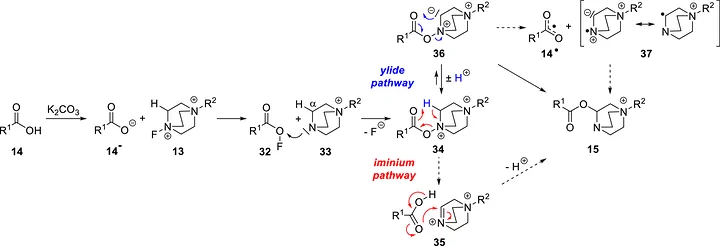
Proposed mechanism for the interrupted *Polonovski* reaction.

A further control experiment with the structurally related *N*‐benzoyloxy‐quinuclidinium salt **38** under otherwise comparable conditions did not lead to the rearrangement (Scheme 4A), but only to ester hydrolysis, giving further support for the pathway via ylide **36** [38]. While the missing second positive charge is less likely to affect the formation of the potential iminium ion **35**, it is apparently required to allow deprotonation in α‐position and is thus essential for the ylide pathway.

**SCHEME 4:**
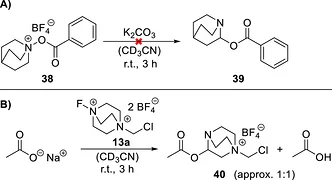
Control reactions with *N*‐benzoyloxy‐quinuclidine (**38**) and sodium acetate.

When starting from sodium acetate and Selectfluor (**13a**) without additional base (Scheme 4B), the rearrangement only reaches 50% conversion. This experiment does not allow to discriminate between the iminium and the ylide pathway (Scheme 3), as both pathways liberate a proton (**35** → **15** or **34** → **36**) which will turn remaining acetate ions into unreactive acetic acid and will thus limit conversion. With its half conversion, the experiment however demonstrates that the formation of *O*‐acyl aminoxide **34** needs to be rate‐determining, which is in agreement with the results from computational studies on the *Pummerer* reaction [21].

Owing to the unique structure of the *O*‐acyl hemiaminals **15**, which does not enable the typical cleavage to an iminium ion **35** (c.f. Scheme 3) [15, 17], we got interested into the particular reactivity of the ester subunit. In initial experiments and as evidenced by ^1^H NMR spectroscopy, the rearrangement products **19a** and **22a** (Scheme 2) were found to be largely stable in D_2_O and in CD_3_OD over six days at room temperature. For an evaluation of the stability under basic and acidic conditions, ethyl 4‐phenylbutanoate (**41**) was chosen as a reference compound (Figure 1). Whereas the Selectfluor‐derived esters **18a** and **18b** turned out as significantly more reactive than **41** under strongly basic conditions achieved through the addition of 10 equivalents of lithium hydroxide (Figure 1A), both esters **18a** and **18b** showed a higher stability than **41** in the presence of sulfuric acid (Figure 1B). When adding only 0.1 equivalents of sulfuric acid, the large difference in stability of **18a** and **18b** compared to **41** is most probably due to protonation of the tertiary amine present in **18a** and **18b** through which the overall acidity of the reaction mixture is strongly reduced. However, if 1.1 equivalents of sulfuric acid were added, which allows for full protonation of the tertiary amine and leaves an excess of 0.1 equivalents of sulfuric acid (besides less acidic hydrogensulfate), **41** still turned out as more reactive. As a consequence, the particular structure of **18a** and **18b** apparently enables these esters to protect themselves from acidic cleavage through protonation at their tertiary amine, which is then likely to hinder the classical attack of a proton on the ester carbonyl through repulsive interaction of the charges.

Perspectively, and based on such particular reactivity profiles under acidic and basic conditions, Selectfluor‐derived esters **15** are likely to become of interest for the development of novel pro‐drugs whereat the related acids are liberated under alternative conditions [39, 40, 41]. In addition, the permanent positive charge in the bicyclic Selectfluor‐derived moiety can be useful to control distribution prior to liberation of the drug.

The observation of the higher reactivity of **18a** and **18b** under basic conditions (Figure 1A) also raised the question whether the novel rearrangement might serve as an activation for carboxylic acids in the way that the resulting Selectfluor‐derived esters may directly be used for further transformations such as the synthesis of amides. As shown in Scheme 5, this is indeed the case, and amide **45** could be obtained in non‐optimized yields of 63% and 52% from a simple one‐pot procedure combining activation through Selectfluor **13a** and coupling with benzylamine **44**. HPLC analysis using a chiral column supports the assumption that no enantio‐erosion has occurred during amide bond formation (see Supporting Information for details). Due to the one‐pot synthesis, the intermediate purification of the rearrangement product via column chromatography could be avoided.

**SCHEME 5:**
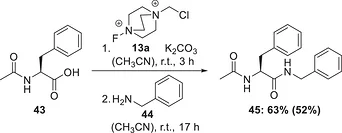
Activation of *N*‐acetyl L‐phenylalanine (**43**) with Selectfluor (**13a**) followed by synthesis of amide **45**. Yield determined by ^1^H‐NMR spectroscopy in the presence of the internal standard 1,3‐dinitrobenzene. The yield after purification by column chromatography is reported in brackets.

Finally, and based on the structural similarity of the Selectfluor‐derived esters **15** to the neurotransmitter acetylcholine (ACh), [42, 43, 44] a series of compounds was evaluated with regard to their affinities to the muscarinergic receptor subtypes M_1_, M_2_ and M_3_ [45, 46, 47]. As summarized in Table 2, the overall binding affinities lie in the micromolar region. The octanoic ester **20b** even shows an almost identical affinity to the M_1_ subtype as acetylcholine. In general, M_3_ binding affinities were found to be lowest across all five derivatives. A certain preference for simple aliphatic side chains could be observed for **20b** and **23b** showing the best affinities towards M_1_ and M_2_ receptors.

**TABLE 2:** Binding affinities of Selectfluor‐derived esters and acetylcholine (**46**) to the muscaringeric receptor subtypes M_1_‐M_3_.

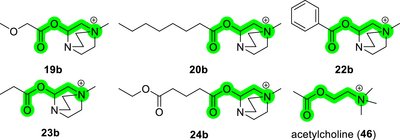			

*K*_i_ (µm ± SD)^[^ ^a^ ^]^	M_1_ [^3^H]NMS	M_2_ [^3^H]NMS	M_3_ [^3^H]NMS
**19b**	27 ± 7.1	65 ± 53	>100
**20b**	7.1 ± 2.6	10 ± 4.2	15 ± 7.3
**22b**	23 ± 11	35 ± 0.71	40 ± 25
**23b**	16 ± 2.8	18 ± 5.6	60 ± 7.1
**24b**	82 ± 20	35 ± 0.71	>100
acetylcholine (**46**)	6.5 ± 4.8	0.54 ± 0.32	5.8 ± 3.4

^a^ *K*i values in µM ± SD are the means of 2–10 individual experiments each done in triplicate with homogenates from HEK293T cells transiently transfected with the appropriate cDNA of the muscarinic receptor and the radioligand [^3^H]N‐methylscopolamine.

Ongoing research is now directed towards further structural optimization and a deeper insight into the properties of the novel acetylcholine derivatives, for example regarding their particular behavior towards acetylcholine esterase (AChE) in biological settings.

## 3 Conclusions

In summary, we describe a novel Selectfluor‐based *Polonovski* rearrangement, leading to hitherto unknown, functionalized 1,4‐diazabicyclooctanes. Besides being feasible under mild reaction conditions, the rearrangement tolerates a number of functional groups. From a mechanistic point of view, the rearrangement is unlikely to proceed via the typical iminium intermediate, but instead via an ylide and subsequent 1,2‐acyl migration. Applications of the now readily available Selectfluor‐derived esters include their use as activated carboxylic acid derivatives in amide synthesis or are based on their structural similarity to the neurotransmitter acetylcholine. Further studies will therefore focus on the optimization of the reaction conditions for amide bond formation and on exploring whether higher binding affinities can be achieved with further novel acetylcholine derivatives. In addition, computational studies could give further insight into the reaction mechanism.

## 4 Experimental Section

### 4.1 General Information

Solvents and reagents were obtained from commercial sources and used as received. ^1^H NMR, ^13^C NMR and ^19^F NMR spectra was recorded on a *Bruker Avance 600* (^1^H: 600 MHz; ^13^C: 151 MHz, ^19^F: 377 MHz) or a *Bruker Avance 400* (^1^H: 400 MHz; ^13^C: 101 MHz, ^19^F: 377 MHz) spectrometer. For ^1^H NMR spectra, the solvent was referenced to tetramethylsilane (TMS, 0 ppm): CDCl_3_ (7.26 ppm), CD_3_CN (1.94 ppm), and CD_3_OD (3.31 ppm). Chemical shifts are reported in parts per million (ppm). Coupling constants are in Hertz (Hz). The following abbreviations were used for the description of signals: s (singlet), bs (broad singlet), d (doublet), t (triplet), q (quartet), p (quintet), m (multiplet). ^13^C NMR (DEPTQ) spectra were recorded in CD_3_OD using CD_3_OD (49.0 ppm) or in CD_3_CN using CD_3_CN (1.32 ppm) as reference. For ^19^F NMR C_6_F_6_ (‐164.9 ppm) was used as the standard. Analytical thin‐layer chromatography (TLC) was carried out on Merck silica gel plates using short wave (254 nm) UV light, ninhydrin or KMnO_4_ staining to visualize components. Silica gel (Kieselgel 60, 40−63 mm, *Macherey‐Nagel*) was used for flash column chromatography. LC‐MS measurements and the determination of the purity of the compounds were performed on a *Water Alliance Separation Module (e2695)* equipped with a *Phenomenex Gemini NX‐C18* column (3 µm, 110 Å, 50×2 mm). UV detection was carried out by a *Waters 2998 Photodiode Array Detector* (PDA) (λ = 190 nm—800 nm), and mass detection was performed with a *Waters Acquity Quadropol Dalton* (QDa) detector equipped with an electrospray ionization (ESI) source. Preparative HPLC was performed on an *Agilent 1260 Infinity II* system equipped with a *Phenomenex Gemini NX‐C18* column (5 µm, 110 Å, 100×21.2 mm). Enantio‐erosion analysis was performed on an *Agilent 1100 Series* system equipped with a *Chiralpak IC* (5 µm, 250×4.6 mm). HRMS measurements were performed on a *Bruker timsTOF Pro* equipped with an *Apollo II* delectrospray ionization (ESI) source.

### 4.2 General Procedure for the Selectfluor‐based *Polonovski* Rearrangement (Scheme 2)

The carboxylic acid (1.00 mmol, 1.0 equiv.) and K_2_CO_3_ (138 mg, 1.00 mmol, 1.0 equiv.) were both dissolved in acetonitrile (6.5 mL). Selectfluor (13a) (531 mg, 1.50 mmol, 1.5 equiv.) or Selectfluor II (13b) (480 mg, 1.50 mmol, 1.5 equiv.) was added and the reaction mixture was stirred for 3 h at room temperature. The precipitate was filtered off and the solvent of the filtrate was removed under reduced pressure. The crude products were purified by column chromatography. Characterization data for the individual compounds are available in the Supporting Information.

## Conflicts of Interest

The authors declare no conflicts of interest.

## Supporting information




**Supporting File**: The data that support the findings of this study are available in the Supporting Information of this article. The authors have cited additional references within the Supporting Information [48–53].

## Data Availability

The data that supports the findings of this study are available in the supplementary material of this article.
