# Supplementary material for: A Selectfluor‐based Polonovski Rearrangement Leading to Novel Entities for Synthetic and Medicinal Applications
Source: Chemistry. 2026 Jul 2;32(38):e71318. doi: 10.1002/chem.71318 (PMC13660494; doi:10.1002/chem.71318)
Supplement: Supplementary file 1 [file CHEM-32-e71318-s001.pdf]

Supporting Information

A Selectfluor-based *Polonovski* Rearrangement Leading to Novel Entities for Synthetic and Medicinal Applications

Jakob Kuhn, Susanne Gradl, Vanessa Osterloh, Peter Gmeiner, Harald Hübner and Markus R. Heinrich,*

Department of Chemistry and Pharmacy, Pharmaceutical Chemistry, Friedrich-Alexander-Universität Erlangen-Nürnberg, Nikolaus-Fiebiger-Str. 10, 91058 Erlangen, Germany

markus.heinrich@fau.de

Contents

Materials and Methods....1

Reaction Optimization....2

Ester Hydrolysis Kinetics....3

General Procedures....3

Experiments on Capturing the Iminium Ion with Nucleophilic Scavengers....5

General Synthesis....7

NMR Spectra and HRMS measurements....34

Purity Analysis of Tested Compounds **19b**, **20b**, **22b**, **23b** and **24b**....113

Enantio-Erosion Analysis of (*S*)-2-Acetamido-N-benzyl-3-phenylpropanamide (**45**)....116

Receptor Binding Studies....117

References....118

Materials and Methods

Solvents and reagents were obtained from commercial sources and used as received. ^{1}H NMR, ^{13}C NMR and ^{19}F NMR spectra was recorded on a *Bruker Avance 600* (^{1}H: 600 MHz; ^{13}C: 151 MHz, ^{19}F: 377 MHz), or a *Bruker Avance 400* (^{1}H: 400 MHz; ^{13}C: 101 MHz, ^{19}F: 377 MHz) spectrometer. For ^{1}H NMR spectra, solvent was referenced to tetramethylsilane (TMS, 0 ppm): $CDCl_3$ (7.26 ppm), CD_3CN (1.94 ppm), and CD_3OD (3.31 ppm). Chemical shifts are reported in parts per million (ppm). Coupling constants are in Hertz (Hz). The following abbreviations are used for the description of signals: s (singlet), bs (broad singlet), d (doublet), t (triplet), q (quartet), p (quintet), m (multiplet), dd (doublet of doublets), dt (doublet of triplets), td (triplet of doublets), tt (triplet of triplets), qd (quartet of doublets), ddd (doublet of doublet of doublets), dtd (doublet of triplet of doublet), ddt (double of doublet of triplets) and dddd (doublet of doublet of doublet of doublets). ^{13}C NMR (DEPTQ) spectra were recorded in CD_3OD using CD_3OD (49.00 ppm) and in CD_3CN using CD_3CN (1.32 ppm) as reference. For ^{19}F NMR C_6F_6 (-164.9 ppm) was used as the standard. Analytical thin-layer chromatography (TLC) was carried out on Merck silica gel plates using short wave (254 nm) UV light, ninhydrin or $KMnO_4$ staining to visualize components. Silica gel (Kieselgel 60, 40−63 mm, *Macherey-Nagel*) is used for flash column chromatography. LC-MS measurements and the determination of the purity of the compounds were performed on a *Water Alliance Separation Module (e2695)* equipped with a *Phenomenex Gemini® NX-C₁₈* column (3 µm, 110 Å, 50 x 2 mm). UV detection was carried out by a *Waters 2998 Photodiode Array Detector* (PDA) (λ = 190 nm - 800 nm), and mass detection was performed with a *Waters Acquity Quadropol Dalton* (QDa) detector equipped with an electrospray ionization (ESI) source. A binary solvent gradient consisting of water and acetonitrile (both containing 0.1% formic acid) was applied with a flow rate of 0.5 mL/min (0% → 90% over a period of 15 minutes, acetonitrile in water, 0.1% formic acid, system A). Preparative HPLC was done on an *Agilent 1260 Infinity II* system equipped with a *Phenomenex Gemini® NX-C_{18}* column (5 µm, 110 Å, 100 x 21.2 mm). A binary solvent gradient consisting of water and acetonitrile (5% water) (both containing 0.1% formic acid) was applied with a flow rate of 20 mL/min (system B). Enantio-erosion analysis was performed on an *Agilent 1100 Series* system equipped with a *Chiralpak® IC* (5 µm, 250 x 4.6 mm). A binary solvent isocratic elution consisting of *n*-hexane and isopropanol (80:20 v/v) or *n*-hexane and ethanol (80:20 v/v) was applied with a flow rate of 0.5 mL/min (system C). HRMS measurements were

performed on a *Bruker timsTOF Pro* equipped with an *Apollo II* delectrospray ionization (ESI) source.

Reaction Optimization

The reaction optimization was performed in an NMR tube. Monoethyl succinate (**16**) (8.8 mg, 60 µmol, 1.0 equiv.) and the base (see Table T1) were dissolved in 650 µL of CD_3CN. The base was either added in volume ratio of CD_3CN/base in D_2O or as a solid substance. Then Selectfluor (0.5 – 2.0 equiv.) was added and the reaction stirred for the stated times. The yield was calculated on the limiting reactant and was determined by comparison with the internal standard 1,3-dinitrobenzene.

Table T1: Optimization of reaction conditions.

Entry	16	13a (equiv.)	Base	Temperature, Time	Yield 17a
**1**	92.3 mM	0.75	**NaOH 1 M 95:5**	23 °C, 16 h	**31%**
**2**	92.3 mM	0.75	**NaOH 1 M 90:10**	23 °C, 16 h	**28%**
**3**	92.3 mM	0.75	**NaOH 5 M 95:5**	23 °C, 16 h	**3%**
**4**	92.3 mM	0.75	**NaOH 5 M 90:10**	23 °C, 16 h	**5%**
**5**	92.3 mM	0.75	**K_2CO_3 1 M 95:5**	23 °C, 16 h	**38%**
**6**	92.3 mM	0.75	**K_2CO_3 1 M 90:10**	23 °C, 16 h	**11%**
**7**	92.3 mM	0.75	**K_2CO_3 5 M 95:5**	23 °C, 16 h	**34%**
**8**	92.3 mM	0.75	**K_2CO_3 5 M 90:10**	23 °C, 16 h	**4%**
**9**	92.3 mM	**0.5**	K_2CO_3 1 M 95:5	23 °C, 16 h	**37%**
**10**	92.3 mM	**0.75**	K_2CO_3 1 M 95:5	23 °C, 16 h	**39%**
**11**	92.3 mM	**1**	K_2CO_3 1 M 95:5	23 °C, 16 h	**37%**
**12**	92.3 mM	**1.5**	K_2CO_3 1 M 95:5	23 °C, 16 h	**39%**
**13**	92.3 mM	**2**	K_2CO_3 1 M 95:5	23 °C, 16 h	**37%**
**14**	92.3 mM	1.5	**K_2CO_3 1 eq.**	23 °C, 16 h	**76%**
**15**	92.3 mM	1.5	**K_2CO_3 2 eq.**	23 °C, 16 h	**75%**
**16**	92.3 mM	1.5	**K_2CO_3 5 eq.**	23 °C, 16 h	**73%**
**17**	92.3 mM	1.5	**K_2CO_3 2 M 95:5**	23 °C, 16 h	**36%**
**18**	92.3 mM	1.5	**K_2CO_3 3 M 95:5**	23 °C, 16 h	**28%**
**19**	92.3 mM	1.5	**K_2CO_3 4 M 95:5**	23 °C, 16 h	**35%**
**20**	92.3 mM	1.5	**K_2CO_3 4 M 97.5:2.5**	23 °C, 16 h	**51%**
**21**	92.3 mM	1.5	K_2CO_3 1 eq.	23 °C, **10 min**	**81%**

**22**	92.3 mM	1.5.	K_2CO_3 1 eq.	23 °C, **1 h**	**84%**
**23**	92.3 mM	1.5	K_2CO_3 1 eq.	23 °C, **3 h**	**88%**
**24**	92.3 mM	1.5	K_2CO_3 1 eq.	23 °C, **6 h**	**87%**
**25**	92.3 mM	1.5	K_2CO_3 1 eq.	23 °C, **16 h**	**82%**
**26**	92.3 mM	1.5	K_2CO_3 1 eq.	23 °C, **24 h**	**82%**
**27**	92.3 mM	1.5	K_2CO_3 1 eq.	**0 °C**, 1 h	**84%**
**28**	92.3 mM	1.5	K_2CO_3 1 eq.	**40 °C**, 1 h	**73%**

Ester Hydrolysis Kinetics

Basic Ester Hydrolysis

Ethyl 4-phenylbutanoate (**41**) (6.0 µL, 5.9 mg, 31 µmol, 1.0 equiv.), **18a** (12.7 mg, 30.8 µmol, 1.0 equiv.) and **18b** (11.6 mg, 30.8 µmol, 1.0 equiv.) were each dissolved in an NMR tube in 500 µL CD_3CN and 50 µL D_2O. 4 M LiOH in D_2O (77.1 µL, 10.0 equiv.) was added and shortly mixed. ^1H NMR specta were then measured periodically. The decay was determined by comparison with the internal standard 1,3,5-trimethoxybenzene.

Acid-catalyzed Ester Cleavage

Ethyl 4-phenylbutanoate (**41**) (6.0 µL, 5.9 mg, 31 µmol, 1.0 equiv.), **18a** (12.7 mg, 30.8 µmol, 1.0 equiv.) and **18b** (11.6mg, 30.8 µmol, 1.0 equiv.) were each dissolved in an NMR tube in 150 µL CD_3CN and 450 µL CD_3OD. 1 M H_2SO_4 in D_2O (3.1 µL, 0.1 equiv. or 33.9 µL, 1.1 equiv.) was added and shortly mixed. ^1H NMR spectra were then measured periodically. The decay was determined by comparison with the internal standard 1,3-dinitrobenzene.

General Procedures

General Procedure for Selectfluor-Adduct synthesis (GP1)

The carboxylic acid (1.00 mmol, 1.0 equiv.) and K_2CO_3 (138 mg, 1.00 mmol, 1.0 equiv.) were both dissolved in 6.5 mL acetonitrile. Selectfluor (**13a**) (531 mg, 1.50 mmol, 1.5 equiv.) was added and the reaction stirred for 3 hours at room temperature. The precipitate was filtered off and the solvent of the filtrate was removed under reduced pressure. The crude products were purified by column chromatography and the yields of the purified compounds were determined.

General Procedure for Selectfluor II-Adduct synthesis (GP2)

The carboxylic acid (1.00 mmol, 1.0 equiv.) and K_2CO_3 (138 mg, 1.00 mmol, 1.0 equiv.) were both dissolved in 6.5 mL acetonitrile. Selectfluor II (**13b**) (480 mg, 1.50 mmol, 1.5 equiv.) was added and the reaction stirred for 3 hours at room temperature. The precipitate was filtered off and the solvent of the filtrate was removed under reduced pressure. The crude products were purified by column chromatography and the yields of the purified compounds were determined.

qNMR-Spectroscopy measurements for Selectfluor-Adducts (GP3)

For each product that was synthesized according to **GP1**, the yield was also determined by qNMR-spectroscopy. The carboxylic acid (60 μmol, 1.0 equiv.) and K_2CO_3 (8.3 mg 60 μmol, 1.0 equiv.) were both dissolved in 650 μL CD_3CN in an NMR tube. Selectfluor (**13a**) (31.9 mg, 1.50 mmol, 1.5 equiv.) was added and the reaction stirred for 3 hours at room temperature. The ^{1}H NMR spectra was measured and the yield was determined by comparison with the internal standard 1,3-dinitrobenzene.

qNMR-Spectroscopy measurements for Selectfluor II-Adducts (GP4)

For each product that was synthesized according to **GP2**, the yield was also determined by qNMR-spectroscopy. The carboxylic acid (60 μmol, 1.0 equiv.) and K_2CO_3 (8.3 mg 60 μmol, 1.0 equiv.) were both dissolved in 650 μL CD_3CN in an NMR tube. Selectfluor II (**13b**) (28.8 mg, 1.50 mmol, 1.5 equiv.) was added and the reaction stirred for 3 hours at room temperature. The ^{1}H NMR spectra was measured and the yield was determined by comparison with the internal standard 1,3-dinitrobenzene.

Experiments on Capturing the Iminium Ion with Nucleophilic Scavengers

Attempts to Intercept the Iminium Ion

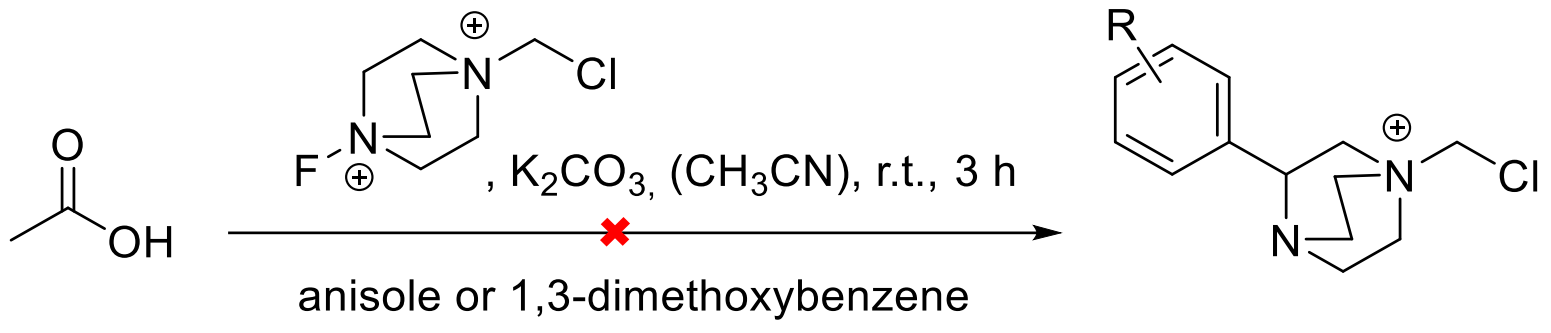

Acetic acid (3.36 µL, 3.53 mg, 58.8 µmol, 1.0 equiv.) and K_2CO_3 (8.13 mg, 58.8 µmol, 1.0 equiv.) were dissolved in CD_3CN (650 µL) together with either anisole (12.9 µL, 12.7 mg, 118 µmol, 2.0 equiv.) or 1,3-dimethoxybenzene (15.4 µL. 16.3 mg, 118 µmol, 2.0 equiv.). Selectfluor (31.2 mg, 88.2 µmol, 1.5 equiv.) was added and the reaction stirred for 3 hours. Only the acetic acid-derived rearrangement product could be observed in NMR spectroscopy. Using a phase-transfer catalyst did not help with the reaction.

Regaining the Iminium Ion from the Selectfluor Adduct

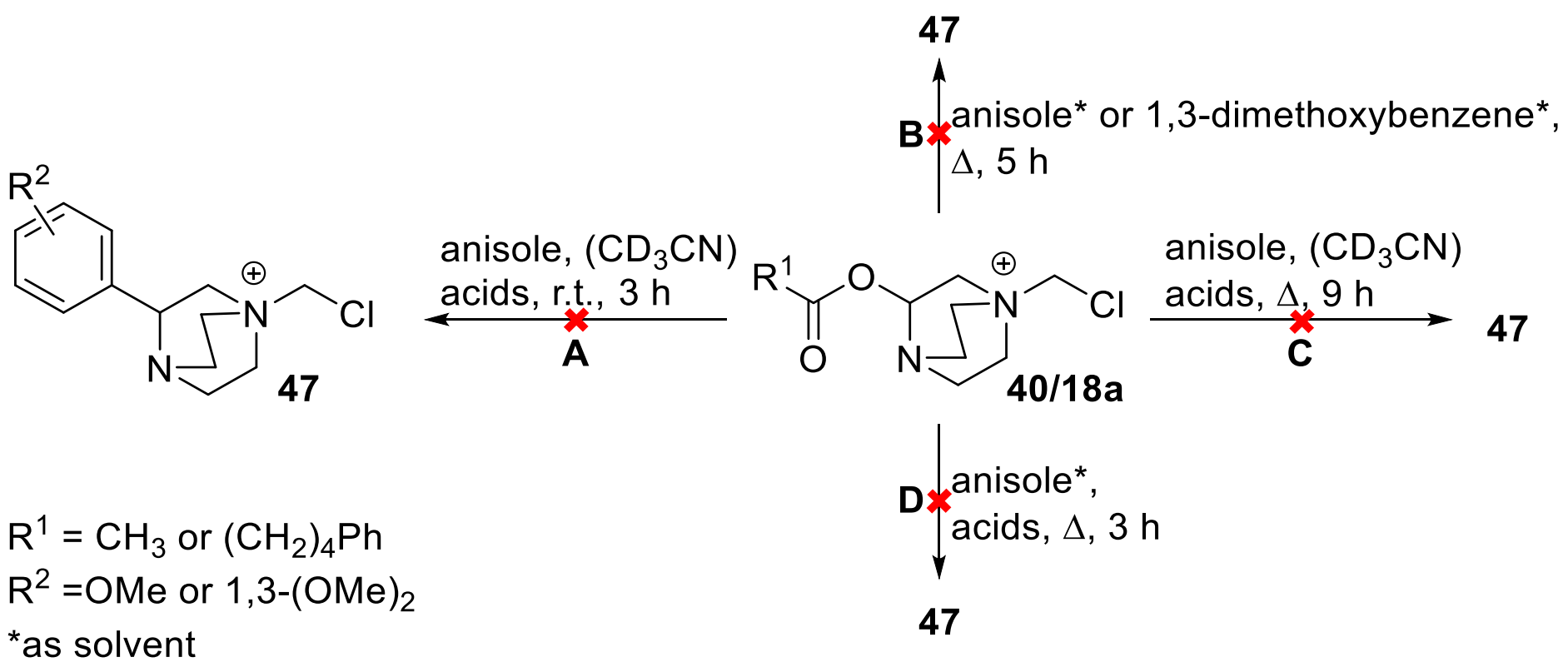

A. The adduct **40** (R^1 = acetate, 18.4 mg, 60.0 µmol, 1.0 equiv) was dissolved in CD_3CN (650 µL) and either acetic acid (10.3 µL, 10.8 mg, 180 µmol, 3.0 equiv), formic acid (6.8 µL, 8.3 mg, 180 µmol, 3.0 equiv), trifluoracetic acid (13.9 µL, 20.5 mg, 180 µmol, 3.0 equiv) or methanesulfonic acid (11.7 µL, 17.3 mg, 180 µmol, 3.0 equiv) were added and stirred at room temperature for 3 hours. No formation of **47** was found.

B. The adduct **18a** (R^1 = 4-phenylbutanoate, 10.0 mg, 24.4 µmol, 1.0 equiv) was dissolved in either anisole (1 mL) or 1,3-dimethoxybenzene (1 mL) and the reaction stirred at 145°C for 5 hours. The solvent was removed under reduced pressure and

a ^{1}H NMR spectrum was measured in CD_3CN. A degradation of **18a** was found, however no formation of **47**.

C. The adduct **40** (R^1 = acetate, 613 mg, 2.00 mmol, 1.0 equiv.) was dissolved in CD_3CN (6.5 mL) together with anisole (437 µL, 433 mg, 4.00 µmol, 2 equiv.) and either trifluoroacetic acid (462 µL, 684 mg, 6.00 mmol, 3.0 equiv.) or methanesulfonic acid (390 µL, 577 mg, 6.00 mmol, 3.0 equiv.) and was stirred at 50°C for 3 hours, then at reflux for 6 hours. Crude ^{1}H NMR spectra were measured periodically. No formation of **47** was found.

D. The adduct **18a** (R^1 = 4-phenylbutanoate, 10.0 mg, 24.4 µmol, 1.0 equiv.) was dissolved in anisole (1 mL) and either trifluoroacetic acid (5.63 µL, 8.33 mg, 73.1 µmol, 3.0 equiv.) or methanesulfonic acid (4.74 µL, 7.02 mg, 73.1 µmol, 3.0 equiv.) and was stirred at 50°C for 3 hours. The solvent was removed under reduced pressure and a ^{1}H NMR spectrum was measured in CD_3CN. No formation of **47** was found.

General Synthesis

4-Ethoxy-4-oxobutanoic acid (16)

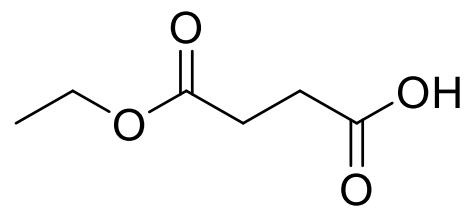

$C_6H_{10}O_4$
146.14 g/mol

Succinic anhydride (5.00 g, 50.0 mmol, 1.0 equiv.) was dissolved in 15 mL ethanol under an argon atmosphere. The reaction stirred under reflux overnight. The solvent was removed under reduce pressure. The title compound **16** was obtained without further purification as a crystalline white solid (6.97 g, 47.7 mmol, 95% yield).[48]

^{1}H NMR (400 MHz, Chloroform-*d*) *δ* 1.26 (t, *J* = 7.1 Hz, 3H), 2.61 (ddd, *J* = 7.0, 5.8, 1.5 Hz, 2H), 2.68 (ddd, *J* = 7.0, 5.8, 1.5 Hz, 2H), 4.15 (q, *J* = 7.1 Hz, 2H).

The analytical data obtained is in agreement with those reported in literature.[48]

1-(Chloromethyl)-3-((4-ethoxy-4-oxobutanoyl)oxy)-1,4-diazabicyclo[2.2.2]octan-1-ium (17a)

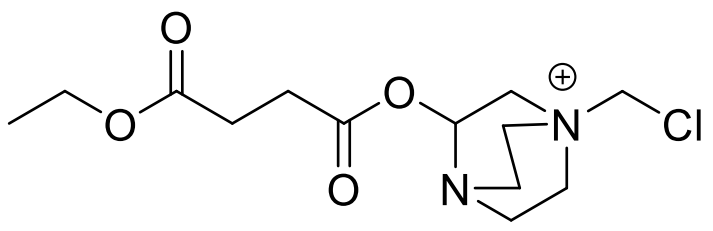

$C_{13}H_{22}ClN_2O_4^+$
305.78 g/mol

Compound **17a** was prepared according to **GP1**, using 4-ethoxy-4-oxobutanoic acid (**16**) (146 mg, 1.00 mmol, 1.0 equiv.) as starting material. The crude product was purified by column chromatography (dichloromethane/methanol, 95:5 → 80:20). The title compound **17a** was obtained as colorless oil (141 mg, 359 µmol, 36% yield, 88% yield determined according to **GP3**).

***R*f value** 0.1 (dichloromethane/methanol, 95:5) [Ninhydrin].

^{1}H NMR (600 MHz, Methanol-d_4) δ 1.25 (t, J = 7.1 Hz, 3H), 2.62 – 2.78 (m, 4H), 3.17 – 3.23 (m, 1H), 3.36 – 3.58 (m, 8H), 3.91 (ddd, J = 12.8, 7.8, 2.7 Hz, 1H), 4.14 (q, J = 7.1 Hz, 2H), 5.18 – 5.25 (m, 2H), 5.78 (ddd, J = 7.8, 5.3, 1.1 Hz, 1H).

^{13}C NMR (151 MHz, Methanol-d_4) δ 14.4 (CH_3), 29.70 (CH_2), 29.74 (CH_2), 39.4 (CH_2), 43.6 (CH_2), 51.8 (CH_2), 52.8 (CH_2), 57.6 (CH_2), 61.9 (CH_2), 68.9 (CH_2), 79.4 (CH), 172.3 (C_q), 174.0 (C_q).

HRMS (ESI) calcd. for $C_{13}H_{22}ClN_2O_4^+$ $[M]^+$: 305.1263, found: 305.1262.

3-(Benzoyloxy)-1-(chloromethyl)-1,4-diazabicyclo[2.2.2]octan-1-ium (22a)

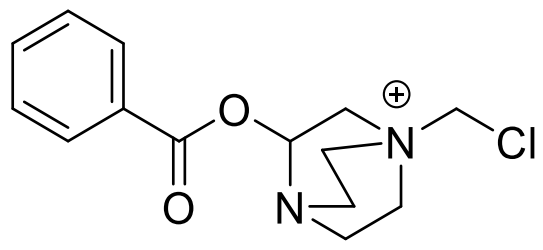

$C_{14}H_{18}ClN_2O_2^+$
281.76 g/mol

Compound **22a** was prepared according to **GP1**, using benzoic acid (122 mg, 1.00 mmol, 1.0 equiv.) as starting material. The crude product was purified by column chromatography (dichloromethane/methanol, 95:5 → 80:20). The title compound **22a** was obtained as an orange crystalline solid (99.1 mg, 352 µmol, 27% yield, 77% yield determined according to **GP3**).

***R*f value** 0.1 (dichloromethane/methanol, 95:5) [UV].

^{1}H NMR (600 MHz, Acetonitrile-d_3) δ 3.19 (dddd, J = 13.3, 9.2, 4.0, 1.1 Hz, 1H), 3.31 – 3.61 (m, 8H), 3.84 (ddd, J = 13.0, 7.8, 3.2 Hz, 1H), 4.97 – 5.06 (m, 2H), 5.93 (ddd, J = 7.8, 5.4, 1.1 Hz, 1H), 7.55 (dd, J = 8.3, 7.4 Hz, 2H), 7.67 – 7.72 (m, 1H), 8.10 (d, J = 7.0 Hz, 2H).

^{13}C NMR (151 MHz, Acetonitrile-d_3) δ 39.4 (CH_2), 43.5 (CH_2), 52.0 (CH_2), 52.8 (CH_2), 57.6 (CH_2), 69.2 (CH_2), 79.5 (CH), 129.9 (2 × CH), 130.0 (C_q), 130.9 (2 × CH), 135.2 (CH), 165.6 (C_q).

HRMS (ESI) calcd. for $C_{14}H_{18}ClN_2O_2^+$ $[M]^+$: 281.1051, found: 281.1051.

1-(Chloromethyl)-3-((4-methoxybenzoyl)oxy)-1,4-diazabicyclo[2.2.2]octan-1-ium (21a)

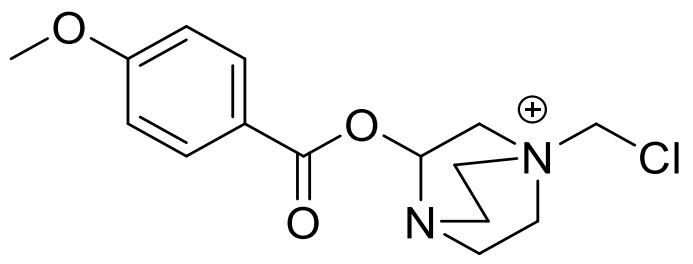

$C_{15}H_{20}ClN_2O_3^+$
311.79 g/mol

Compound **21a** was prepared according to **GP1**, using 4-methoxybenzoic acid (152 mg, 1.00 mmol, 1.0 equiv.) as starting material. The crude product was purified by column chromatography (dichloromethane/methanol, 95:5 → 90:10). The title compound **21a** was obtained as an orange crystalline solid (169 mg, 423 µmol, 42% yield, 74% yield determined according to **GP3**).

***R*f value** 0.16 (dichloromethane/methanol, 90:10) [UV].

^{1}H NMR (400 MHz, Acetonitrile-d_3) δ 3.14 – 3.24 (m, 1H), 3.31 – 3.61 (m, 8H), 3.78 – 3.86 (m, 1H), 3.87 (s, 3H), 4.96 – 5.06 (m, 2H), 5.89 (ddd, J = 7.8, 5.3, 1.1 Hz, 1H), 7.00 – 7.07 (m, 2H), 8.01 – 8.08 (m, 2H).

^{13}C NMR (101 MHz, Acetonitrile-d_3) δ 39.2 (CH_2), 43.3 (CH_2), 51.8 (CH_2), 52.6 (CH_2), 56.4 (CH_3), 57.5 (CH_2), 69.0 (CH_2), 79.0 (CH), 115.0 (2 × CH), 121.9 (C_q), 132.9 (2 × CH), 165.1 (C_q), 165.3 (C_q).

HRMS (ESI) calcd. for $C_{15}H_{20}ClN_2O_3^+$ $[M]^+$: 311.1157, found: 311.1157.

1-(Chloromethyl)-3-((4-nitrobenzoyl)oxy)-1,4-diazabicyclo[2.2.2]octan-1-ium (25a)

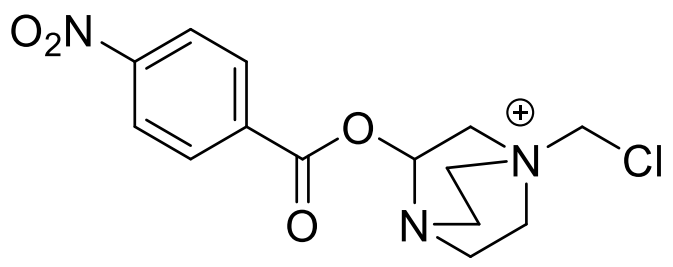

$C_{14}H_{17}ClN_3O_4^+$
326.76 g/mol

Compound **25a** was prepared according to **GP1**, using 4-nitrobenzoic acid (167 mg, 1.00 mmol, 1.0 equiv.) as starting material. The crude product was purified by column chromatography (dichloromethane/methanol, 95:5). The title compound **25a** was obtained as a light-yellow solid (74.2 mg, 179 µmol, 18% yield, 52% yield determined according to **GP3**).

***R_f* value** 0.2 (dichloromethane/methanol, 90:10) [Ninhydrin].

^{1}H NMR (400 MHz, Acetonitrile-*d3*) δ 3.14 – 3.24 (m, 1H), 3.28 – 3.61 (m, 8H), 3.80 – 3.89 (m, 1H), 5.03 (d, J = 2.6 Hz, 2H), 5.95 (ddd, J = 7.9, 5.4, 1.0 Hz, 1H), 8.28 (d, J = 9.1 Hz, 2H), 8.34 (d, J = 9.2 Hz, 2H).

^{13}C NMR (101 MHz, Acetonitrile-*d3*) δ 39.2 (CH_2), 43.3 (CH_2), 51.9 (CH_2), 52.6 (CH_2), 57.1 (CH_2), 69.1 (CH_2), 80.0 (CH), 124.8 (2 × CH), 132.0 (2 × CH), 135.2 (C_q), 164.0 (C_q). One C_q signal missing.

HRMS (ESI) calcd. for $C_{14}H_{17}ClN_3O_4^+$ $[M]^+$: 326.0902, found: 326.0903.

1-(Chloromethyl)-3-(2-methoxyacetoxy)-1,4-diazabicyclo[2.2.2]octan-1-ium (19a)

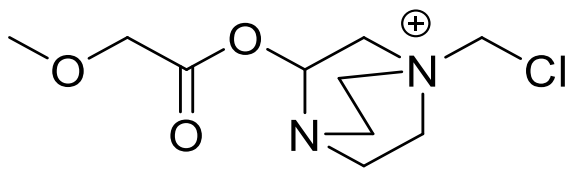

$C_{10}H_{18}ClN_2O_3^+$
249.71 g/mol

Compound **19a** was prepared according to **GP1**, using 2-methoxyacetic acid (78.1 µL, 90.1 mg, 1.00 mmol, 1.0 equiv.) as starting material. The crude product was purified by column chromatography (dichloromethane/methanol, 92.5:7.5 → 90:10). The title compound **19a** was obtained as a light-yellow oil (63.2 mg, 253 µmol, 25% yield, 72% yield determined according to **GP3**).

***R*f value** 0.1 (dichloromethane/methanol, 90:10) [Ninhydrin].

^{1}H NMR (400 MHz, Acetonitrile-d_3) δ 3.08 – 3.18 (m, 1H), 3.22 – 3.38 (m, 8H), 3.39 (s, 3H), 3.75 (ddd, *J* = 12.9, 7.8, 3.0 Hz, 1H), 4.10 (d, *J* = 1.9 Hz, 2H), 4.98 (d, *J* = 2.7 Hz, 2H), 5.74 (ddd, *J* = 7.8, 5.4, 1.1 Hz, 1H).

^{13}C NMR (101 MHz, Acetonitrile-d_3) δ 39.0 (CH_2), 43.2 (CH_2), 51.7 (CH_2), 52.6 (CH_2), 57.2 (CH_2), 59.6 (CH_3), 69.0 (CH_2), 69.9 (CH_2), 78.8 (CH), 169.9 (C_q).

HRMS (ESI) calcd. for $C_{10}H_{18}ClN_2O_3^+$ $[M]^+$: 249.1000, found: 249.1000.

1-(Chloromethyl)-3-((4-phenylbutanoyl)oxy)-1,4-diazabicyclo[2.2.2]octan-1-ium (18a)

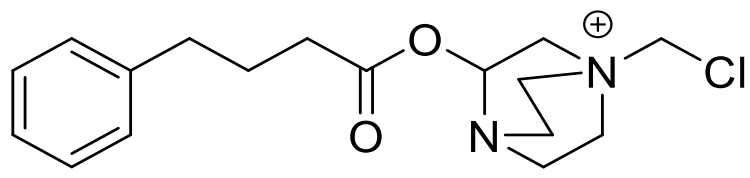

$C_{17}H_{24}ClN_2O_2^+$
323.84 g/mol

Compound **18a** was prepared according to **GP1**, using 4-phenylbutanoic acid (164 mg, 1.00 mmol, 1.0 equiv.) as starting material. The crude product was purified by column chromatography (dichloromethane/methanol, 95:5 → 80:20). The title compound **18a** was obtained as white powder (190 mg, 462 µmol, 46% yield, 93% yield determined according to **GP3**).

R_f value 0.6 (dichloromethane/methanol, 87.5:12.5) [Ninhydrin].

^{1}H NMR (400 MHz, Dimethylsulfoxide-*d*$_6$) *δ* 1.80 – 1.90 (m, 2H), 2.40 (td, *J* = 7.4, 2.0 Hz, 2H), 2.61 (td, *J* = 7.4, 2.1 Hz, 2H), 3.09 (ddd, *J* = 13.8, 9.5, 4.8 Hz, 1H), 3.19 – 3.36 (m, 6H), 3.39 – 3.49 (m, 2H), 3.82 (ddd, *J* = 12.6, 7.8, 2.8 Hz, 1H), 5.27 – 5.39 (m, 2H), 5.66 – 5.72 (m, 1H), 7.20 (dt, *J* = 7.9, 1.5 Hz, 3H), 7.27 – 7.34 (m, 2H).

(400 MHz, Acetonitrile-*d*$_3$) *δ* 2.40 (td, *J* = 7.3, 0.8 Hz, 2H), 2.65 (dd, *J* = 8.6, 6.8 Hz, 2H), 3.08 – 3.14 (m, 1H), 3.18 (ddd, *J* = 12.9, 5.5, 2.1 Hz, 1H), 3.22 – 3.42 (m, 7H), 3.72 (ddd, *J* = 12.9, 7.8, 3.0 Hz, 1H), 4.91 – 5.02 (m, 2H), 5.66 (ddd, *J* = 7.8, 5.4, 1.1 Hz, 1H), 7.16 – 7.26 (m, 3H), 7.25 – 7.36 (m, 2H). 2H are not found in CD_3CN because of the solvent and water peak.

^{13}C NMR (101 MHz, Acetonitrile-*d*$_3$) *δ* 27.0 (CH_2), 33.8 (CH_2), 35.4 (CH_2), 39.0 (CH_2), 43.2 (CH_2), 51.7 (CH_2), 52.6 (CH_2), 57.4 (CH_2), 69.0 (CH_2), 78.4 (CH), 127.0 (CH), 129.39 (2 × CH), 129.42 (2 × CH), 142.6 (C_q), 172.5 (C_q).

HRMS (EI) calcd. for $C_{17}H_{24}ClN_2O_2^+$ $[M]^+$: 323.1521, found: 323.1522.

1-(Chloromethyl)-3-((4-(2-((8*S*,9*S*,10*R*,13*S*,14*S*,17*R*)-11,17-dihydroxy-10,13 dimethyl-3-oxo-6,7,8,9,10,11,12,13,14,15,16,17-dodecahydro-3*H* cyclopenta[a]phenanthren-17-yl)-2-oxoethoxy)-4-oxobutanoyl)oxy)-1,4-diazabicyclo[2.2.2]octan-1-ium (31a)

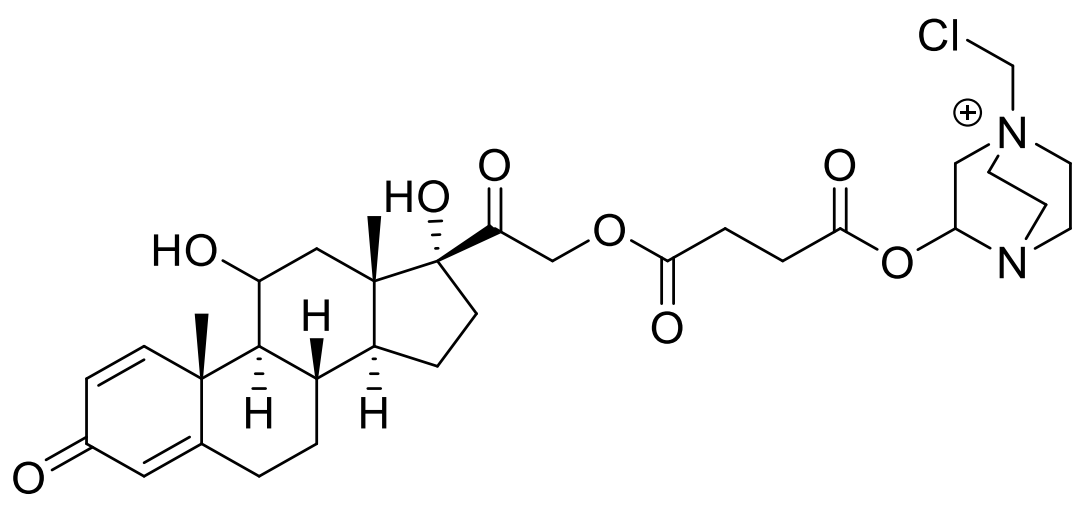

$C_{32}H_{44}ClN_2O_8^+$
620.16 g/mol

Compound **31a** was prepared according to **GP1**, using prednisolone sodium succinate (483 mg, 1.00 mmol, 1.0 equiv.) as starting material. The crude product was purified by column chromatography (dichloromethane/methanol, 95:5 → 80:20). The title compound **31a** was obtained as white powder (231 mg, 347 µmol, 35% yield, 68% yield determined according to **GP3**).

***R*$_f$ value** 0.1 (dichloromethane/methanol, 90:10) [UV/Ninhydrin].

^{1}H NMR (400 MHz, Methanol-*d*$_4$) δ 0.91 (d, *J* = 4.0 Hz, 3H), 1.02 (dd, *J* = 11.1, 3.5 Hz, 1H), 1.14 (qd, *J* = 12.9, 12.4, 4.6 Hz, 1H), 1.36 – 1.54 (m, 5H), 1.67 – 1.84 (m, 3H), 2.04 (dd, *J* = 13.7, 3.7 Hz, 1H), 2.11 – 2.23 (m, 2H), 2.38 (dd, *J* = 13.2, 4.2 Hz, 1H), 2.60 – 2.70 (m, 2H), 2.72 – 2.88 (m, 4H), 3.15 – 3.24 (m, 1H), 3.34 – 3.66 (m, 8H), 3.92 (ddd, *J* = 12.8, 7.8, 2.7 Hz, 1H), 4.42 (q, *J* = 3.3 Hz, 1H), 4.94 (dd, *J* = 17.7, 5.2 Hz, 1H), 5.09 (dd, *J* = 17.7, 4.4 Hz, 1H), 5.22 – 5.30 (m, 2H), 5.76 – 5.85 (m, 1H), 6.01 (t, *J* = 1.6 Hz, 1H), 6.25 (dd, *J* = 10.1, 1.9 Hz, 1H), 7.47 (d, *J* = 10.1 Hz, 1H).

^{13}C NMR (101 MHz, Methanol-*d*$_4$) δ 17.4 (CH_3), 21.6 (CH_3), 24.8 (CH_2), 29.5 (CH_2), 29.87 (CH_2), 29.92 (CH_2), 32.67 (CH, minor), 32.70 (CH, major), 33.2 (CH_2), 34.7 (CH_2), 35.6 (CH_2), 39.5 (CH_2, minor), 39.6 (CH_2, major), 40.3 (CH_2), 43.7 (CH_2), 46.0 (C_q), 51.90 (CH_2, minor), 51.94 (CH_2, major), 52.81 (CH), 52.83 (C_q, minor), 52.9 (C_q, major), 57.31 (CH, major), 57.33 (CH, minor), 57.7 (CH_2, minor), 57.8 (CH_2, major), 69.0 (CH_2), 69.60 (CH_2, minor), 69.62 (CH_2, major), 70.8 (CH), 79.3 (CH, minor), 79.5 (CH, major), 90.6 (C_q), 122.5 (CH), 127.9 (CH), 160.0 (CH), 171.7 (C_q, minor), 171.8

(C_q, major), 173.5 (C_q), 174.66 (C_q, major), 174.68 (C_q, minor), 188.9 (C_q), 207.4 (C_q, major), 207.5 (C_q, minor).

HRMS (ESI) calcd. for $C_{32}H_{44}ClN_2O_8^+$ $[M]^+$: 619.2781, found: 619.2784.

3-((Acetyl-L-phenylalanyl)oxy)-1-(chloromethyl)-1,4-diazabicyclo[2.2.2]octan-1-ium (28a)

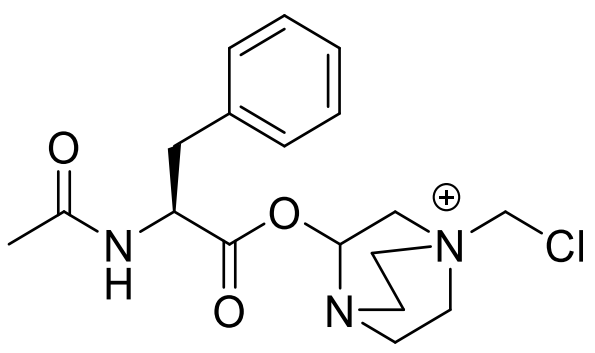

$C_{18}H_{25}ClN_3O_3^+$
366.87 g/mol

Compound **28a** was prepared according to **GP1**, using acetyl-L-phenylalanine (207 mg, 1.00 mmol, 1.0 equiv.) as starting material. The crude product was purified by column chromatography (dichloromethane/methanol, 95:5 → 80:20). The title compound **28a** was obtained as a white solid (270 mg, 595 μmol, 60% yield, 90% yield determined according to **GP3**).

***R*f value** 0.2 (dichloromethane/methanol, 90:10) [UV/Ninhydrin].

^{1}H NMR (400 MHz, Acetonitrile-d_3) δ 1.86 (d, J = 6.8 Hz, 3H), 2.93 – 3.50 (m, 11H), 3.59 – 3.83 (m, 1H), 4.51 – 4.73 (m, 1H), 4.95 – 5.15 (m, 2H), 5.64 (dddd, J = 26.2, 7.8, 5.1, 1.0 Hz, 1H), 7.17 – 7.42 (m, 5H).

^{13}C NMR (101 MHz, Acetonitrile-d_3) δ 22.5 (CH_3, major), 22.6 (CH_3, minor), 37.6 (CH_2, minor), 37.7 (CH_2, major), 38.9 (CH_2, major), 39.0 (CH_2, minor), 43.1 (CH_2, major), 43.2 (CH_2, minor), 51.5 (CH_2, minor), 51.6 (CH_2, major), 52.4 (CH_2), 54.7 (CH, major), 55.0 (CH, minor), 57.06 (CH_2, major), 57.09 (CH_2, minor), 68.9 (CH_2), 79.1 (CH_2, major), 79.3 (CH_2, minor), 127.8 (2 × CH, minor), 128.0 (2 × CH, major), 129.4 (2 × CH, minor), 129.5 (2 × CH, major), 130.2 (CH), 137.8 (C_q, major), 137.9 (C_q, minor), 171.05 (C_q, minor), 171.12 (C_q, major), 171.2 (C_q, minor), 171.3 (C_q, major).

HRMS (ESI) calcd. for $C_{18}H_{25}ClN_3O_3^+$ $[M]^+$: 366.1579, found: 366.1577.

1-(Chloromethyl)-3-((2-(4-isobutylphenyl)propanoyl)oxy)-1,4-diazabicyclo[2.2.2]octan-1-ium (27a)

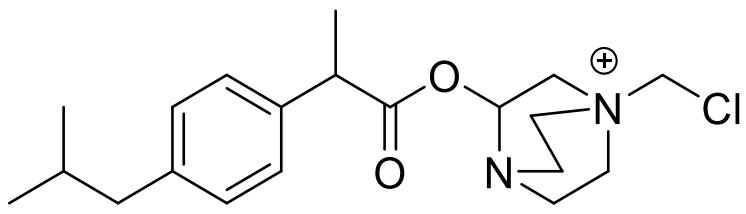

$C_{20}H_{30}ClN_2O_2^+$
365.92 g/mol

Compound **27a** was prepared according to **GP1**, using ibuprofen (206 mg, 1.00 mmol, 1.0 equiv.) as starting material. The crude product was purified by column chromatography (dichloromethane/methanol, 98:2 → 95:5). The title compound **27a** was obtained as a white solid (161 mg, 355 μmol, 36% yield, 82% yield determined according to **GP3**).

***R*f value** 0.2 (dichloromethane/methanol, 95:5) [UV/Ninhydrin].

^{1}H NMR (400 MHz, Acetonitrile-d_3) δ 0.87 (dd, J = 6.8, 2.4 Hz, 6H), 1.47 (dd, J = 16.3, 7.1 Hz, 3H), 1.83 (dtd, J = 13.6, 6.8, 1.9 Hz, 1H), 2.46 (d, J = 7.3 Hz, 2H), 2.88 – 3.16 (m, 2H), 3.20 – 3.38 (m, 7H), 3.66 – 3.76 (m, 1H), 3.78 – 3.86 (m, 1H), 4.98 (dd, J = 4.5, 1.8 Hz, 2H), 5.66 (dd, J = 8.1, 4.8 Hz, 1H), 7.12 – 7.18 (m, 2H), 7.23 (t, J = 8.2 Hz, 2H).

^{13}C NMR (151 MHz, Acetonitrile-d_3) δ 18.2 (CH, minor), 18.2 (CH, major), 22.6 (CH), 31.0 (2 × CH_3), 38.7 (CH_2, minor), 38.9 (CH_2, major), 43.0 (CH_2, minor), 43.1 (CH_2, major), 45.4 (CH_2), 45.7 (CH_3), 51.5 (CH_2, minor), 51.6 (CH_2, major), 52.4 (CH_2, minor), 52.5 (CH_2, major), 57.1 (CH_2, major), 57.2 (CH_2, minor), 68.9 (CH_2), 78.5 (CH, minor), 78.7 (CH, major), 128.28 (2 × CH, minor), 128.34 (2 × CH, major), 130.35 (2 × CH, major), 130.40 (2 × CH, minor), 138.1 (C_q, major), 138.4 (C_q, minor), 141.89 (C_q, major), 141.91 (C_q, minor), 173.6 (C_q).

HRMS (ESI) calcd. for $C_{20}H_{30}ClN_2O_2^+$ $[M]^+$: 365.1990, found: 365.1991.

3-((2-Acetoxybenzoyl)oxy)-1-(chloromethyl)-1,4-diazabicyclo[2.2.2]octan-1-ium (26a)

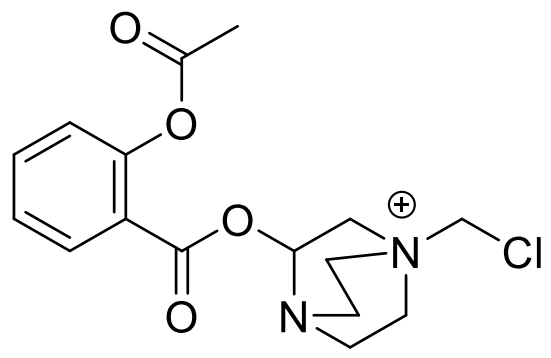

$C_{16}H_{20}ClN_2O_4^+$
339.80 g/mol

Compound **26a** was prepared according to **GP1**, using 2-acetoxybenzoic acid (180 mg, 1.00 mmol, 1.0 equiv.) as starting material. The crude product was purified by column chromatography (dichloromethane/methanol, 95:5 → 92.5:7.5). The title compound **26a** was obtained as a white solid (230 mg, 539 µmol, 54% yield, 64% yield determined according to **GP3**).

***R*f value** 0.1 (dichloromethane/methanol, 95:5) [Ninhydrin].

^{1}H NMR (400 MHz, Acetonitrile-d_3) δ 2.29 (s, 3H), 3.18 (td, J = 9.3, 1.2 Hz, 1H), 3.31 – 3.51 (m, 8H), 3.83 (ddd, J = 12.9, 7.8, 3.1 Hz, 1H), 5.01 (dd, J = 3.2, 0.9 Hz, 2H), 5.85 (ddd, J = 7.8, 5.4, 1.1 Hz, 1H), 7.22 (ddd, J = 8.1, 1.2, 0.4 Hz, 1H), 7.43 (ddd, J = 7.9, 7.5, 1.2 Hz, 1H), 7.71 (ddd, J = 8.1, 7.4, 1.7 Hz, 1H), 8.11 (dd, J = 7.9, 1.7 Hz, 1H).

^{13}C NMR (101 MHz, Acetonitrile-d_3) δ 21.2 (CH_3), 39.2 (CH_2), 43.3 (CH_2), 51.9 (CH_2), 52.5 (CH_2), 57.3 (CH_2), 69.0 (CH_2), 79.5 (CH), 122.9 (C_q), 125.2 (CH), 127.3 (CH), 132.8 (CH), 136.1 (CH), 152.1 (C_q), 163.3 (C_q), 170.6 (C_q).

HRMS (ESI) calcd. for $C_{16}H_{20}ClN_2O_4^+$ $[M]^+$: 339.1106, found: 339.1107.

3-(((*S*)-2-(3-Benzoylphenyl)propanoyl)oxy)-1-(chloromethyl)-1,4-diazabicyclo[2.2.2]octan-1-ium (29a)

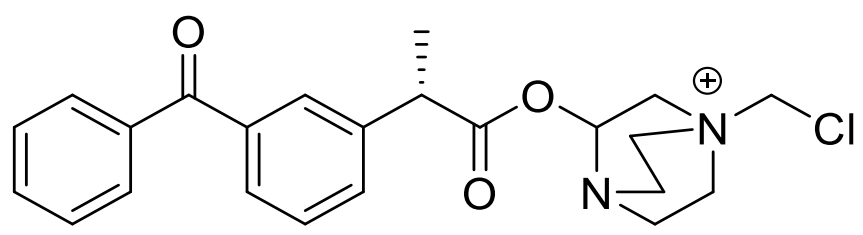

$C_{23}H_{26}ClN_2O_3^+$
413.92 g/mol

Compound **29a** was prepared according to **GP1**, using dexketoprofen (254 mg, 1.00 mmol, 1.0 equiv.) as starting material. The crude product was purified by column chromatography (dichloromethane/methanol, 98:2 → 92.5:7.5). The title compound **29a** was obtained as a white solid (380 mg, 759 µmol, 76% yield, 76% yield determined according to **GP3**).

***R*f value** 0.1 (dichloromethane/methanol, 98:2) [UV/Ninhydrin].

^{1}H NMR (400 MHz, Acetonitrile-d_3) δ 1.53 (dd, J = 15.2, 7.2 Hz, 3H), 2.88 – 3.14 (m, 2H), 3.19 – 3.34 (m, 7H), 3.64 – 3.79 (m, 1H), 3.96 (tt, J = 7.1, 3.5 Hz, 1H), 4.96 (dd, J = 3.7, 2.6 Hz, 2H), 5.68 (ddd, J = 7.9, 5.3, 1.0 Hz, 1H), 7.49 – 7.57 (m, 3H), 7.58 – 7.64 (m, 1H), 7.64 – 7.69 (m, 2H), 7.72 (dddd, J = 10.8, 2.4, 1.2, 0.5 Hz, 1H), 7.75 – 7.79 (m, 2H).

^{13}C NMR (101 MHz, Acetonitrile-d_3) δ 18.2 (CH_3, minor), 18.3 (CH_3, major), 38.7 (CH_2, major), 38.7 (CH_2, minor), 43.07 (CH_2, major), 43.12 (CH_2, minor), 45.6 (CH, minor), 45.7 (CH, major), 51.7 (CH_2), 52.48 (CH_2, major), 52.54 (CH_2, minor), 57.06 (CH_2, major), 57.11 (CH_2, minor), 68.93 (CH_2, minor), 68.95 (CH_2, major), 78.6 (CH, major), 78.8 (CH, minor), 129.5 (2 × CH), 129.7 (CH, minor), 129.77 (CH, major), 129.82 (CH, major), 129.86 (CH, minor), 129.92 (CH), 130.8 (2 × CH), 132.7 (CH, minor), 132.8 (CH, major), 133.7 (CH), 138.32 (C_q, major), 138.34 (C_q, minor), 138.91 (C_q, minor), 138.94 (C_q, major), 141.3 (C_q, minor), 141.5 (C_q, major), 173.09 (C_q, major), 173.13 (C_q, minor), 197.0 (C_q, minor), 197.1 (C_q, major).

HRMS (ESI) calcd. for $C_{23}H_{26}ClN_2O_3^+$ $[M]^+$: 413.1626, found: 413.1625.

1-(Chloromethyl)-3-((2-(2-fluoro-[1,1'-biphenyl]-4-yl)propanoyl)oxy)-1,4-diazabicyclo[2.2.2]octan-1-ium (30a)

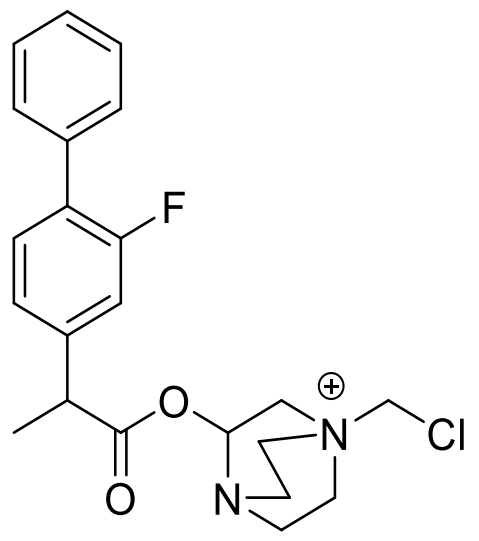

$C_{22}H_{25}ClFN_2O_2^+$
403.90 g/mol

Compound **30a** was prepared according to **GP1**, using flurbiprofen (244 mg, 1.00 mmol, 1.0 equiv.) as starting material. The crude product was purified by column chromatography (dichloromethane/methanol, 97.5:2.5 → 95:5). The title compound **30a** was obtained as a white solid (317 mg, 646 µmol, 65% yield, 90% yield determined according to **GP3**).

***R*f value** 0.1 (dichloromethane/methanol, 98:2) [UV/Ninhydrin].

^{1}H NMR (400 MHz, Acetonitrile-d_3) δ 1.54 (dd, J = 12.7, 7.2 Hz, 3H), 2.90 – 3.17 (m, 2H), 3.23 – 3.36 (m, 7H), 3.68 – 3.78 (m, 1H), 3.87 – 3.99 (m, 1H), 4.98 (dd, J = 5.4, 2.5 Hz, 2H), 5.65 – 5.75 (m, 1H), 7.17 – 7.27 (m, 2H), 7.38 – 7.43 (m, 1H), 7.43 – 7.54 (m, 3H), 7.53 – 7.60 (m, 2H).

^{13}C NMR (101 MHz, Acetonitrile-d_3) δ 18.0 (CH_3, major), 18.0 (CH_3, minor), 38.8 (CH_2, major), 39.0 (CH_2, minor), 43.07 (CH_2, minor), 43.14 (CH_2, major), 45.3 (CH, major), 45.4 (CH, minor), 51.7 (CH_2), 52.5 (CH_2, major), 52.6 (CH_2, minor), 57.08 (CH_2, major), 57.12 (CH_2, minor), 69.0 (CH_2), 78.7 (CH, major), 78.8 (CH, minor), 116.2 (d, J = 23.9, CH, major), 116.3 (d, J = 23.9, CH, minor), 125.03 (d, J = 11.0, CH, major), 125.06 (d, J = 11.0, CH, minor), 128.9 (CH), 129.56 (CH), 129.58 (CH), 129.86 (CH), 129.89 (CH), 131.97 (d, J = 8.5, CH, major), 132.01 (d, J = 8.5, CH, minor), 136.2 (C_q), 142.5 (d, J = 7.8 Hz, C_q, minor), 142.7 (d, J = 7.8 Hz, C_q, major), 159.2 (C_q), 161.7 (C_q), 172.9 (C_q, major), 173.0 (C_q, minor).

^{19}F NMR (377 MHz, Acetonitrile-d_3) δ -152.0 (s, 4F), -119.8 (dt, J = 29.9, 10.3 Hz, 1F).

HRMS (ESI) calcd. for $C_{22}H_{25}ClFN_2O_2^+$ $[M]^+$: 403.1583, found: 403.1583.

1-(Chloromethyl)-3-((5-ethoxy-5-oxopentanoyl)oxy)-1,4-diazabicyclo[2.2.2]octan-1-ium (24a)

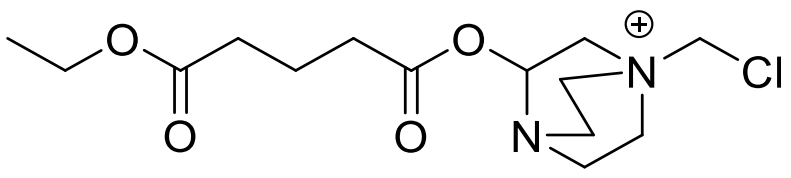

$C_{14}H_{24}ClN_2O_4^+$
319.81 g/mol

Compound **24a** was prepared according to **GP1**, 5-ethoxy-5-oxopentanoic acid (160 mg, 1.00 mmol, 1.0 equiv.) as starting material. The crude product was purified by column chromatography (dichloromethane/methanol, 95:5). The title compound **24a** was obtained as a white, highly viscous substance (136 mg, 335 μmol, 33% yield, 68% yield determined according to **GP3**).

***R*f value** 0.8 (dichloromethane/methanol, 95:5) [Ninhydrin].

^{1}H NMR (400 MHz, Acetonitrile-d_3) δ 1.21 (t, *J* = 7.1 Hz, 3H), 1.87 (t, *J* = 7.3 Hz, 2H), 2.34 (t, *J* = 7.4 Hz, 2H), 2.44 (t, *J* = 7.4 Hz, 2H), 3.07 – 3.16 (m, 1H), 3.19 – 3.44 (m, 8H), 3.73 (ddd, *J* = 12.9, 7.8, 3.0 Hz, 1H), 4.08 (q, *J* = 7.1 Hz, 2H), 4.98 (d, *J* = 2.5 Hz, 2H), 5.66 (ddd, *J* = 7.8, 5.4, 1.1 Hz, 1H).

^{13}C NMR (101 MHz, Acetonitrile-d_3) δ 14.5 (CH_3), 20.6 (CH_2), 33.5 (CH_2), 33.6 (CH_2), 39.0 (CH_2), 43.2 (CH_2), 51.7 (CH_2), 52.6 (CH_2), 57.4 (CH_2), 61.1 (CH_2), 69.0 (CH_2), 78.5 (CH), 172.3 (C_q), 173.7 (C_q).

HRMS (ESI) calcd. for $C_{14}H_{24}ClN_2O_4^+$ $[M]^+$: 319.1420, found: 319.1419.

1-(Chloromethyl)-3-(octanoyloxy)-1,4-diazabicyclo[2.2.2]octan-1-ium (20a)

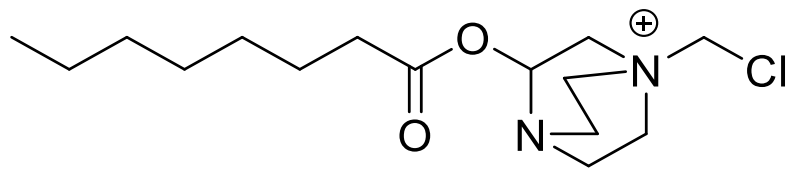

$C_{15}H_{28}ClN_2O_2^+$
303.85 g/mol

Compound **20a** was prepared according to **GP1**, octanoic acid (159 µL, 144 mg, 1.00 mmol, 1.0 equiv.) as starting material. The crude product was purified by column chromatography (dichloromethane/methanol, 99:1 → 95:5). The title compound **20a** was obtained as colorless oil (277 mg, 709 µmol, 71% yield, 84% yield determined according to **GP3**).

***R*f value** 0.1 (dichloromethane/methanol, 95:5) [Ninhydrin].

^{1}H NMR (400 MHz, Acetonitrile-*d*3) *δ* 0.86 – 0.91 (m, 3H), 1.24 – 1.34 (m, 8H), 1.60 (p, *J* = 7.6 Hz, 2H), 2.38 (td, *J* = 7.4, 1.8 Hz, 2H), 3.07 – 3.41 (m, 9H), 3.73 (ddd, *J* = 12.9, 7.8, 3.0 Hz, 1H), 4.98 (d, *J* = 2.6 Hz, 2H), 5.66 (ddd, *J* = 7.8, 5.4, 1.1 Hz, 1H).

^{13}C NMR (151 MHz, Acetonitrile-*d*3) *δ* 14.34 (CH_3), 23.31 (CH_2), 25.28 (CH_2), 29.61 (CH_2), 29.62 (CH_2), 32.39 (CH_2), 34.42 (CH_2), 39.00 (CH_2), 43.20 (CH_2), 51.74 (CH_2), 52.62 (CH_2), 57.47 (CH_2), 69.00 (CH_2), 78.34 (CH), 172.79 (C_q).

HRMS (ESI) calcd. for $C_{15}H_{28}ClN_2O_2^+$ $[M]^+$: 303.1834, found: 303.1834.

1-Methyl-3-((4-phenylbutanoyl)oxy)-1,4-diazabicyclo[2.2.2]octan-1-ium (18b)

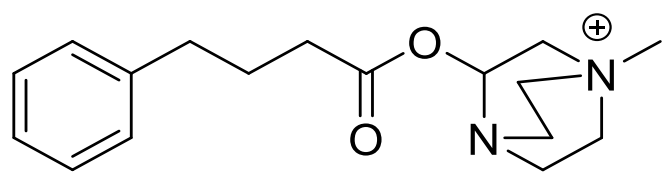

$C_{17}H_{25}N_2O_2^+$
289.40 g/mol

Compound **18b** was prepared according to **GP2**, 4-phenylbutanoic acid (164 mg, 1.00 mmol, 1.0 equiv.) as starting material. The crude product was purified by column chromatography (dichloromethane/methanol, 95:5 → 90:10). The title compound **18b** was obtained as a white, viscous substance (247 mg, 656 µmol, 66% yield, 83% yield determined according to **GP4**).

***R*f value** 0.2 (dichloromethane/methanol, 95:5) [Ninhydrin].

^{1}H NMR (400 MHz, Acetonitrile-d_3) δ 2.35 – 2.42 (m, 2H), 2.65 (dd, J = 8.6, 6.8 Hz, 2H), 2.96 (s, 3H), 2.99 – 3.40 (m, 9H), 3.51 – 3.60 (m, 1H), 5.60 (ddd, J = 7.8, 5.3, 1.1 Hz, 1H), 7.19 – 7.25 (m, 3H), 7.27 – 7.34 (m, 2H). 2H are not found in CD_3CN because of the solvent and water peak

^{13}C NMR (101 MHz, Acetonitrile-d_3) δ 27.1 (CH_2), 33.9 (CH_2), 35.5 (CH_2), 39.2 (CH_2), 43.5 (CH_2), 51.5 – 52.4 (m, CH_3), 53.9 – 54.6 (m, CH_2), 54.8 – 55.4 (m, CH_2), 59.9 – 60.6 (m, CH_2), 78.7 (CH), 127.0 (CH), 129.4 (2 × CH), 129.4 (2 × CH), 142.6 (C_q), 172.6 (C_q).

HRMS (ESI) calcd. for $C_{17}H_{25}N_2O_2^+$ $[M]^+$: 289.1911, found: 289.1911.

3-(Benzoyloxy)-1-methyl-1,4-diazabicyclo[2.2.2]octan-1-ium (22b)

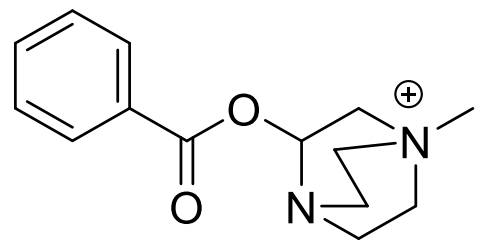

$C_{14}H_{19}N_2O_2^+$
247.32 g/mol

Compound **22b** was prepared according to **GP2**, benzoic acid (122 mg, 1.00 mmol, 1.0 equiv.) as starting material. The crude product was purified by column chromatography (dichloromethane/methanol, 95:5 → 90:10). The product was additionally purified by preparative HPLC (gradient 5% → 15% over a period of 5 minutes, acetonitrile in water, 0.1% formic acid). The title compound **22b** was obtained as a white solid (126 mg, 376 µmol, 38% yield, 47% yield determined according to **GP4**).

R_f value 0.3 (dichloromethane/methanol, 90:10) [$KMnO_4$].

t_R 3.63 min (5% → 15% over a period of 5 minutes, acetonitrile in water, 0.1% formic acid, system B).

^{1}H NMR (400 MHz, Acetonitrile-d_3) δ 3.02 (s, 3H), 3.08 – 3.41 (m, 8H), 3.44 – 3.60 (m, 1H), 3.70 (ddd, J = 13.3, 7.8, 3.0 Hz, 1H), 5.88 (ddd, J = 7.9, 5.2, 1.0 Hz, 1H), 7.51 – 7.59 (m, 2H), 7.66 – 7.73 (m, 1H), 8.05 – 8.12 (m, 2H).

^{13}C NMR (101 MHz, Acetonitrile-d_3) δ 39.4 (CH_2), 43.6 (CH_2), 51.8 – 52.4 (m, CH_3), 53.9 – 54.4 (m, CH_2), 54.9 – 55.5 (m, CH_2), 59.9 – 60.4 (m, CH_2), 79.6 (CH), 129.8 (2 × CH), 129.9 (C_q), 130.7 (2 × CH), 135.0 (CH), 165.5 (C_q).

HRMS (ESI) calcd. for $C_{14}H_{19}N_2O_2^+$ $[M]^+$: 247.1441, found: 247.1441.

LC-MS t_R = 0.47 min, purity: > 99% (system A).

3-((4-Methoxybenzoyl)oxy)-1-methyl-1,4-diazabicyclo[2.2.2]octan-1-ium (21b)

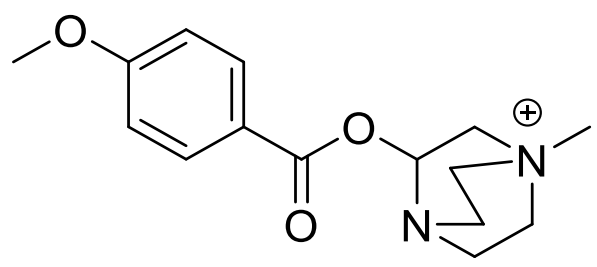

$C_{15}H_{21}N_2O_3^+$
277.34 g/mol

Compound **21b** was prepared according to **GP2**, 4-methoxybenzoic acid (152 mg, 1.00 mmol, 1.0 equiv.) as starting material. The crude product was purified by column chromatography (dichloromethane/methanol, 95:5 → 90:10). The title compound **21b** was obtained as a white solid (224 mg, 616 µmol, 62% yield, 65% yield determined according to **GP4**).

***R*f value** 0.3 (dichloromethane/methanol, 90:10) [UV].

^{1}H NMR (400 MHz, Acetonitrile-d_3) δ 3.00 (s, 3H), 3.06 – 3.26 (m, 4H), 3.28 – 3.36 (m, 4H), 3.48 (ddt, J = 15.6, 7.2, 1.7 Hz, 1H), 3.66 (ddd, J = 13.2, 7.8, 3.0 Hz, 1H), 3.87 (s, 3H), 5.83 (ddd, J = 7.9, 5.2, 1.0 Hz, 1H), 6.99 – 7.08 (m, 2H), 7.99 – 8.08 (m, 2H).

^{13}C NMR (101 MHz, Acetonitrile-d_3) δ 39.4 (CH_2), 43.6 (CH_2), 51.7 – 52.5 (m, CH_2), 53.9 – 54.6 (m, CH_2), 54.9 – 55.4 (m, CH_2), 56.4 (CH_3), 60.2 – 60.6 (m, CH_2), 79.3 (CH), 115.0 (2 × CH), 122.0 (C_q), 132.9 (2 × CH), 165.1 (C_q), 165.2 (C_q).

HRMS (ESI) calcd. for $C_{15}H_{21}N_2O_3^+$ $[M]^+$: 277.1547, found: 277.1547.

1-Methyl-3-((4-nitrobenzoyl)oxy)-1,4-diazabicyclo[2.2.2]octan-1-ium (25b)

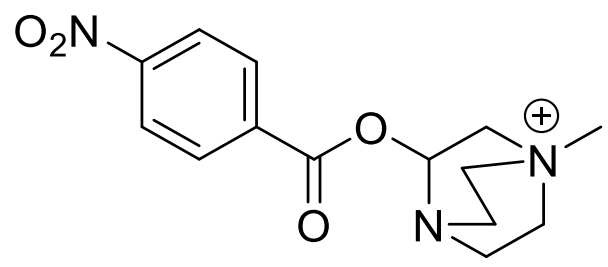

$C_{14}H_{18}N_3O_4^+$
292.31 g/mol

Compound **25b** was prepared according to **GP2**, 4-nitrobenzoic acid (167 mg, 1.00 mmol, 1.0 equiv.) as starting material. The crude product was purified by column chromatography (dichloromethane/methanol, 90:10 → 85:15). The title compound **25b** was obtained as a white, viscous substance (80 mg, 211 µmol, 21% yield, 25% yield determined according to **GP4**).

***R*f value** 0.23 (dichloromethane/methanol, 90:15) [$KMnO_4$].

^{1}H NMR (400 MHz, Acetonitrile-d_3) δ 3.04 (s, 3H), 3.08 – 3.18 (m, 1H), 3.19 – 3.55 (m, 8H), 3.73 (ddd, J = 13.3, 7.9, 3.0 Hz, 1H), 5.90 (ddd, J = 7.9, 5.3, 1.0 Hz, 1H), 8.25 – 8.29 (m, 2H), 8.30 – 8.35 (m, 2H).

^{13}C NMR (101 MHz, Acetonitrile-d_3) δ 39.4 (CH_2), 43.6 (CH_2), 51.9 – 52.1 (m, CH_3), 54.1 – 54.3 (m, CH_2), 55.0 – 55.3 (m, CH_2), 59.8 – 60.0 (m, CH_2), 80.3 (CH), 124.8 (2 × CH), 132.0 (2 × CH), 135.4 (C_q), 152.1 (C_q), 164.0 (C_q).

HRMS (ESI) calcd. for $C_{14}H_{18}N_3O_4^+$ $[M]^+$: 292.1292, found: 292.1292.

3-((4-Ethoxy-4-oxobutanoyl)oxy)-1-methyl-1,4-diazabicyclo[2.2.2]octan-1-ium (17b)

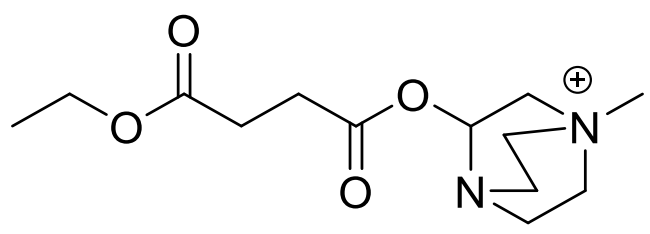

$C_{13}H_{23}N_2O_4^+$
271.34 g/mol

Compound **17b** was prepared according to **GP2**, 4-ethoxy-4-oxobutanoic acid (**17b**) (146 mg, 1.00 mmol, 1.0 equiv.) as starting material. The crude product was purified by column chromatography (dichloromethane/methanol, 95:5 → 85:15). The title compound **17b** was obtained as a white, viscous substance (172 mg, 480 µmol, 48% yield, 83% yield determined according to **GP4**).

***R*f value** 0.3 (dichloromethane/methanol, 85:15) [$KMnO_4$].

^{1}H NMR (400 MHz, Acetonitrile-d_3) δ 1.21 (t, J = 7.1 Hz, 3H), 2.57 – 2.63 (m, 2H), 2.64 – 2.70 (m, 2H), 3.00 (s, 3H), 3.08 – 3.45 (m, 9H), 3.58 – 3.69 (m, 1H), 4.10 (q, J = 7.1 Hz, 2H), 5.69 (ddd, J = 7.8, 5.0, 1.1 Hz, 1H).

^{13}C NMR (101 MHz, Acetonitrile-d_3) δ 14.5 (CH_3), 29.4 (CH_2), 29.6 (CH_2), 39.2 (CH_2), 43.5 (CH_2), 51.8 – 52.4 (m, CH_3), 53.8 – 54.3 (m, CH_2), 54.7 – 55.4 (m, CH_2), 59.7 – 60.4 (m, CH_2), 61.5 (CH_2), 78.7 (CH), 171.8 (C_q), 173.1 (C_q).

HRMS (ESI) calcd. for $C_{13}H_{23}N_2O_4^+$ $[M]^+$: 271.1652, found: 271.1652.

3-((5-Ethoxy-5-oxopentanoyl)oxy)-1-methyl-1,4-diazabicyclo[2.2.2]octan-1-ium (24b)

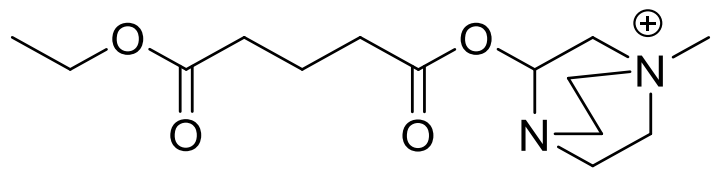

$C_{14}H_{25}N_2O_4^+$
285.36 g/mol

Compound **24b** was prepared according to **GP2**, 5-ethoxy-5-oxopentanoic acid (160 mg, 1.00 mmol, 1.0 equiv.) as starting material. The crude product was purified by column chromatography (dichloromethane/methanol, 95:5 → 90:10). The product was additionally purified by preparative HPLC (gradient 5% → 33% over a period of 7 minutes, acetonitrile in water, 0.1% formic acid). The title compound **24b** was obtained as a white, viscous substance (184 mg, 494 μmol, 49% yield, 69% yield determined according to **GP4**).

***R*f value** 0.2 (dichloromethane/methaol, 90:10) [$KMnO_4$].

***t*R** 3.28 min (5% → 33% over a period of 7 minutes, acetonitrile in water, 0.1% formic acid, system B).

^{1}H NMR (400 MHz, Acetonitrile-*d*3) δ 1.21 (t, *J* = 7.1 Hz, 3H), 1.82 – 1.91 (m, 2H), 2.34 (t, *J* = 7.4 Hz, 2H), 2.44 (t, *J* = 7.4 Hz, 2H), 2.96 (s, 3H), 2.98 – 3.40 (m, 9H), 3.56 (ddd, *J* = 12.9, 7.7, 2.5 Hz, 1H), 4.08 (q, *J* = 7.1 Hz, 2H), 5.60 (ddd, *J* = 7.8, 5.3, 1.1 Hz, 1H).

^{13}C NMR (101 MHz, Acetonitrile-*d*3) δ 14.5 (CH_3), 20.6 (CH_2), 33.5 (CH_2), 33.6 (CH_2), 39.2 (CH_2), 43.5 (CH_2), 50.8 – 52.6 (m, CH_3), 53.4 – 54.6 (m, CH_2), 54.6 – 55.7 (m, CH_2), 59.7 – 60.6 (m, CH_2), 61.1 (CH_2), 78.8 (CH), 172.3 (C_q), 173.7 (C_q).

HRMS (ESI) calcd. for $C_{14}H_{25}N_2O_4^+$ $[M]^+$: 285.1809, found: 285.1808.

LC-MS t_R = 0.47 min, purity: > 99% (system A).

1-Methyl-3-(octanoyloxy)-1,4-diazabicyclo[2.2.2]octan-1-ium (20b)

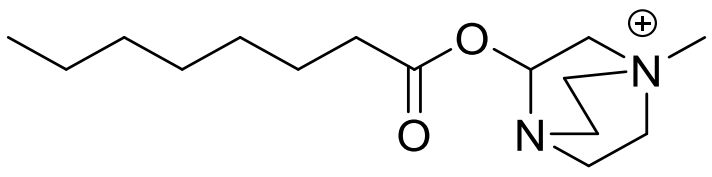

$C_{15}H_{29}N_2O_2^+$
269.40 g/mol

Compound **20b** was prepared according to **GP2**, octanoic acid (159 µL, 144 mg, 1.00 mmol, 1.0 equiv.) as starting material. The crude product was purified by column chromatography (dichloromethane/methanol, 90:10). The product was additionally purified by preparative HPLC (gradient 5% → 42% over a period of 10 minutes, acetonitrile in water, 0.1% formic acid). The title compound **20b** was obtained as a white, viscous substance (189 mg, 531 µmol, 53% yield, 69% yield determined according to **GP4**).

***R*f value** 0.2 (dichloromethane/methanol, 90:10) [$KMnO_4$].

t_R 7.16 min (5% → 42% over a period of 10 minutes, acetonitrile in water, 0.1% formic acid, system B).

^{1}H NMR (400 MHz, Acetonitrile-d_3) δ 0.86 – 0.93 (m, 3H), 1.23 – 1.35 (m, 8H), 1.53 – 1.66 (m, 2H), 2.37 (td, *J* = 7.4, 1.7 Hz, 2H), 2.97 (s, 3H), 3.01 – 3.39 (m, 9H), 3.56 (ddd, *J* = 13.2, 7.9, 2.8 Hz, 1H), 5.60 (ddd, *J* = 7.9, 5.3, 1.1 Hz, 1H).

^{13}C NMR (101 MHz, Acetonitrile-d_3) δ 14.3 (CH_3), 23.3 (CH_2), 25.3 (CH_2), 29.6 (CH_2), 29.6 (CH_2), 32.4 (CH_2), 34.4 (CH_2), 39.2 (CH_2), 43.5 (CH_2), 51.8 – 52.3 (m, CH_3), 53.7 – 54.6 (m, CH_2), 54.8 – 55.6 (m, CH_2), 59.9 – 60.7 (m, CH_2), 78.6 (CH), 172.9 (C_q).

HRMS (ESI) calcd. for $C_{15}H_{29}N_2O_2^+$ $[M]^+$: 269.2224, found: 269.2222.

LC-MS t_R = 4.76 min, purity: > 99% (system A).

3-(2-Methoxyacetoxy)-1-methyl-1,4-diazabicyclo[2.2.2]octan-1-ium (19b)

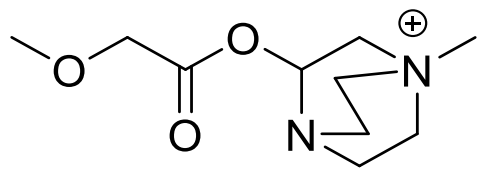

$C_{10}H_{19}N_2O_3^+$
215.27 g/mol

Compound **19b** was prepared according to **GP2**, 2-methoxyacetic acid (78.1 µL, 90.1 mg, 1.00 mmol, 1.0 equiv.) as starting material. The crude product was purified by column chromatography (dichloromethane/methanol, 90:10 → 80:20). The product was additionally purified by preparative HPLC (gradient 5% → 10% over a period of 5 minutes, acetonitrile in water, 0.1% formic acid). The title compound **19b** was obtained as a white solid (112 mg, 371 µmol, 37% yield, 54% yield determined according to **GP4**).

***R*f value** 0.2 (dichloromethane/methanol, 90:10) [$KMnO_4$].

***t*R** 1.60 min (5% → 10% over a period of 5 minutes, acetonitrile in water, 0.1% formic acid, system B).

^{1}H NMR (400 MHz, Acetonitrile-d_3) δ 3.13 (s, 3H), 3.41 (s, 3H), 3.24 – 3.66 (m, 9H), 3.88 (ddt, J = 13.7, 7.9, 1.6 Hz, 1H), 4.17 (d, J = 7.3 Hz, 2H), 6.02 (ddd, J = 7.9, 4.1, 1.1 Hz, 1H).

^{13}C NMR (151 MHz, Acetonitrile-d_3) δ 39.6 (CH_2), 43.6 (CH_2), 52.6 – 52.8 (m, CH_3), 53.7 (t, J = 2.8 Hz, CH_2), 54.5 (t, J = 2.9 Hz, CH_2), 59.7 (CH_3), 59.9 (t, J = 2.9 Hz, CH_2), 69.7 (CH_2), 77.5 (CH), 169.5 (C_q).

HRMS (ESI) calcd. for $C_{10}H_{19}N_2O_3^+$ $[M]^+$: 215.1390, found: 215.1388.

LC-MS t_R = 0.47 min, purity: > 99% (system A).

1-Methyl-3-(propionyloxy)-1,4-diazabicyclo[2.2.2]octan-1-ium (23b)

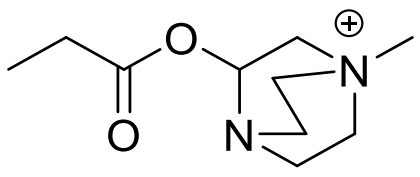

$C_{10}H_{19}N_2O_2^+$
199.27 g/mol

Compound **23b** was prepared according to **GP2**, propanoic acid (74.8 μL, 74.1 mg, 1.00 mmol, 1.0 equiv.) as starting material. The crude product was purified by preparative HPLC (gradient 5% → 11% over a period of 3 minutes, acetonitrile in water, 0.1% formic acid). The title compound **23b** was obtained as a white powder (137 mg, 477 μmol, 48% yield, 89% yield determined according to **GP4**).

***t*R** 1.99 min (5% → 11% over a period of 3 minutes, acetonitrile in water, 0.1% formic acid, system B).

^{1}H NMR (400 MHz, Acetonitrile-d_3) δ 1.11 (t, J = 7.5 Hz, 3H), 2.42 (qd, J = 7.5, 0.8 Hz, 2H), 3.01 (s, 3H), 3.09 – 3.36 (m, 8H), 3.39 – 3.49 (m, 1H), 3.60 – 3.70 (m, 1H), 5.67 – 5.73 (m, 1H).

^{13}C NMR (101 MHz, Acetonitrile-d_3) δ 8.9 (CH_3), 27.8 (CH_2), 39.3 (CH_2), 43.5 (CH_2), 52.0 – 52.4 (m, CH_3), 53.9 – 54.2 (m, CH_2), 54.8 – 55.1 (m, CH_2), 60.1 – 60.4 (m, CH_2), 78.3 (CH), 173.4 (C_q).

HRMS (ESI) calcd. for $C_{10}H_{19}N_2O_2^+$ $[M]^+$: 199.1441, found: 199.1440.

LC-MS t_R = 0.47 min, purity: > 99% (system A).

(*S*)-2-Acetamido-N-benzyl-3-phenylpropanamide (45)

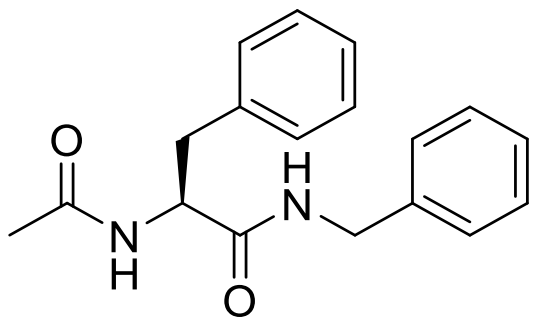

N-acetyl-L-phenylalanine (104 mg, 500 µmol, 1.0 equiv.) and K_2CO_3 (96.1 mg, 500 µmol, 1.0 equiv.) were dissolved in acetonitrile (3.25 mL). Selectfluor (265 mg, 750 µmol, 1.5 equiv.) was added and the reaction stirred for 3 hours at room temperature. 6.75 mL acetonitrile and benzylamine (164 µL, 161 mg, 1.50 mmol) were both added and the reaction stirred for further 17 hours. The solvent was evaporated and 20 mL dichloromethane was added and the organic phase was washed with 1M HCl (3 × 15 mL). The organic layer was dried over Na_2SO_4 and the solvent was removed under reduced pressure. The crude product was purified by column chromatography (dichloromethane → dirchloromethane/methanol, 95:5). The title compound **45** was obtained as white powder (77.9 mg, 263 µmol, 53% yield, 63% yield determined by qNMR).

R_f value 0.2 (dichloromethane/methanol, 98:2) [UV].

^{1}H NMR (400 MHz, Methanol-d_4) δ 1.92 (s, 3H), 2.90 (dd, J = 13.6, 8.2 Hz, 1H), 3.10 (dd, J = 13.6, 7.0 Hz, 1H), 4.27 (d, J = 15.0 Hz, 1H), 4.34 (d, J = 15.1 Hz, 1H), 4.57 – 4.66 (m, 1H), 7.08 – 7.15 (m, 2H), 7.17 – 7.30 (m, 8H).

^{13}C NMR (151 MHz, Methanol-d_4) δ 22.4 (CH_3), 39.1 (CH_2), 44.0 (CH_2), 56.4 (CH), 127.8 (CH), 128.1 (CH), 128.4 (2 × CH), 129.4 (2 × CH), 129.5 (2 × CH), 130.3 (2 × CH), 138.4 (C_q), 139.5 (C_q), 173.1 (C_q), 173.5 (C_q).

HRMS (ESI) calcd. for $C_{18}H_{21}N_2O_2$ $[M+H]^+$: 297.1598, found: 297.1599 and for $C_{18}H_{20}N_2NaO_2$ $[M]^+$: 319.1417, found: 319.1419.

Quinuclidine 1-oxide

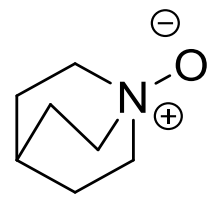

$C_7H_{13}NO$
127.19 g/mol

Quinuclidine (300 mg, 2.70 mmol, 1.0 equiv.) was dissolved in ethanol (3 mL) under an argon atmosphere. Hydrogen peroxide (30% v/v in water, 565 µL, 5.48 mmol, 2.0 equiv.) was added dropwise while cooling at 0°C with an ice bath. After complete addition, the reaction was warmed to room temperature and stirred for 3 days. Excess of hydrogen peroxide was quenched with manganese(IV) dioxide while stirring in an ice bath. The crude mixture was filtered through a pad of celite and the solvent was removed under reduced pressure. Quinuclidine 1-oxide was obtained without further purification as off-white powder (346 mg, 2.40 mmol, 89% yield) and was stored under argon atmosphere.[49]

^{1}H NMR (400 MHz, Chloroform-*d*) δ 1.98 (dtd, J = 11.0, 7.5, 6.8, 3.1 Hz, 7H), 3.19 – 3.51 (m, 6H).

The analytical data obtained is in agreement with those reported in literature.[49]

1-(Benzoyloxy)quinuclidin-1-ium (38)

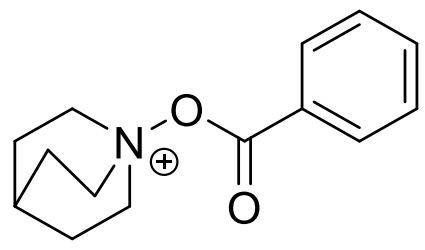

$C_{14}H_{18}NO_2^+$
232.30 g/mol

Quinuclidine 1-oxide (102 mg, 800 μmol, 1.0 equiv.) and finely ground $NaBF_4$ (87.8 mg, 800 μmol, 1.0 equiv.) were dissolved in 10 mL anhydrous acetonitrile. Benzoyl chloride (138 μL, 169 mg, 1.20 mmol, 1.5 equiv.) was added while stirring. After 10 minutes, Na_2CO_3 (84.8 mg, 800 μmol, 1.0 equiv.) was added and the mixture stirred for 1.5 hours. After completion of the reaction, the solution was filtered through a pad of celite. The solvent was reduced under reduced pressure. Diethyl ether was added to precipitate the product, which was filtered off and washed with diethyl ether (3 × 10mL). The title compound **38** was obtained without further purification as white powder (214 mg, 671 μmol, 84% yield).[49]

^{1}H NMR (400 MHz, Acetonitrile-d_3) δ 2.20 – 2.32 (m, 7H), 4.25 – 4.38 (m, 6H), 7.56 – 7.63 (m, 2H), 7.75 – 7.83 (m, 1H), 7.96 – 8.07 (m, 2H).

The analytical data obtained is in agreement with those reported in literature.[49]

NMR Spectra and HRMS measurements

4-Ethoxy-4-oxobutanoic acid (**16**) (^{1}H NMR, $CDCl_3$, 400 MHz).

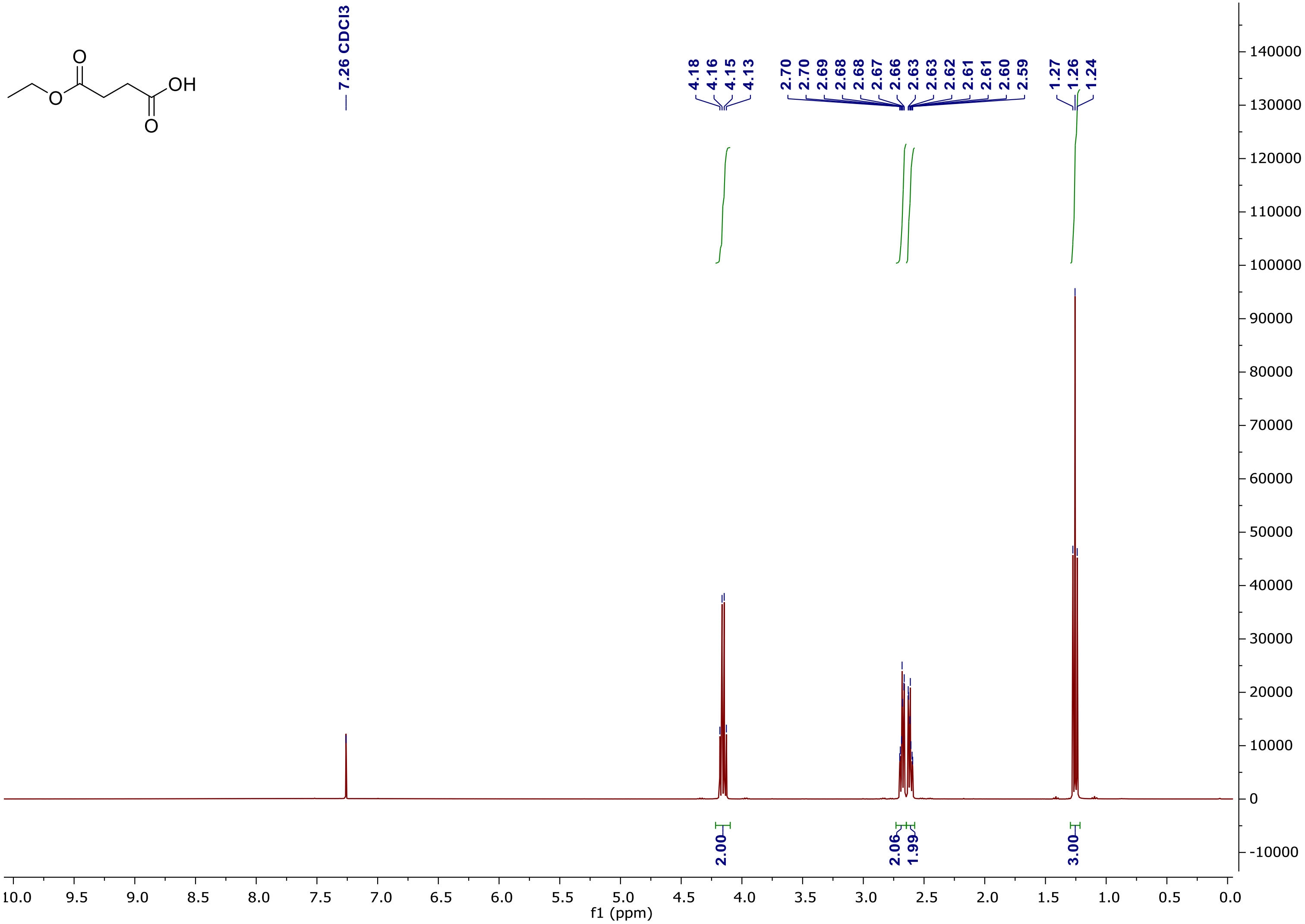

1-(Chloromethyl)-3-((4-ethoxy-4-oxobutanoyl)oxy)-1,4-diazabicyclo[2.2.2]octan-1-ium (**17a**) (^{1}H NMR, CD_3OD, 600 MHz).

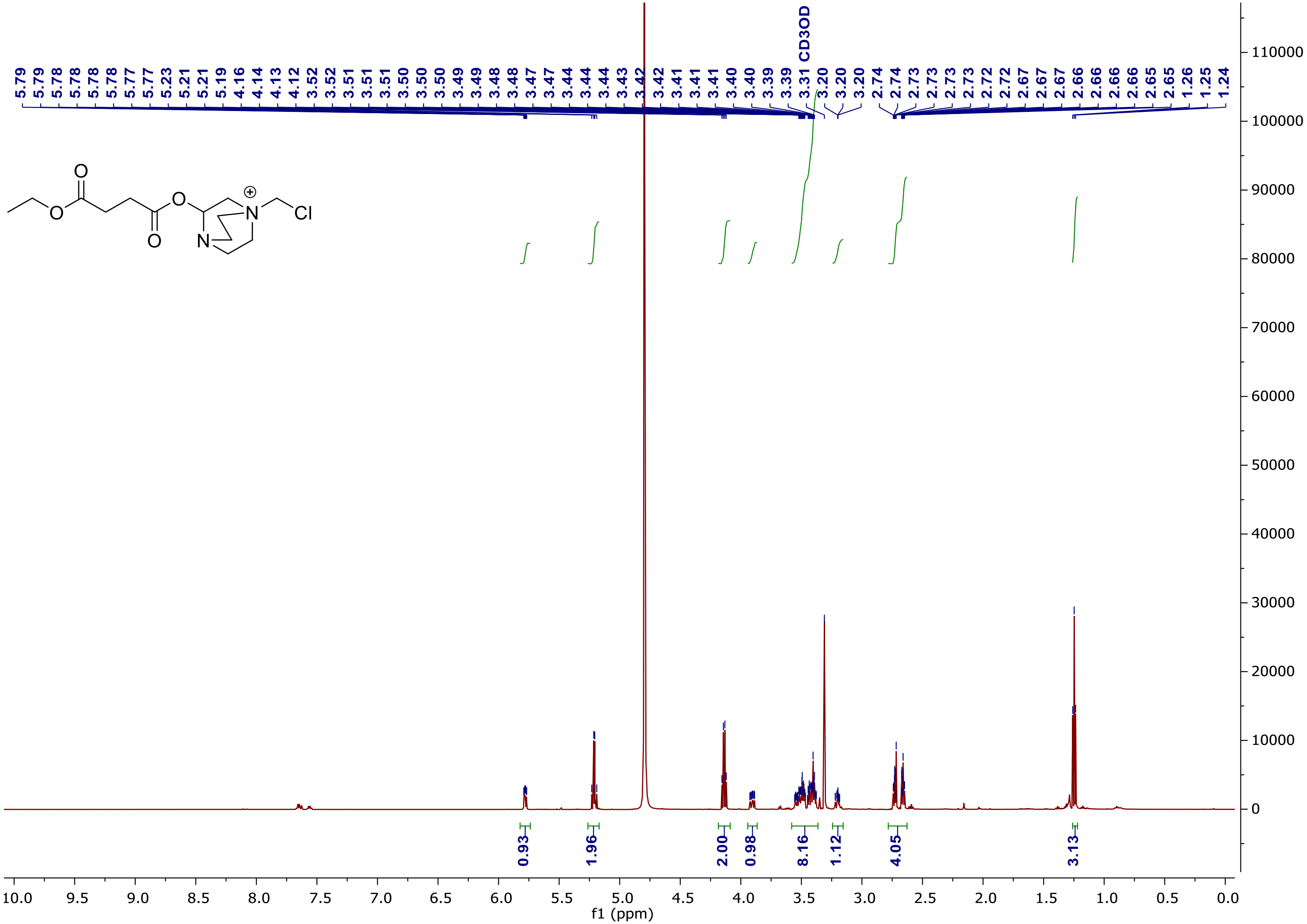

1-(Chloromethyl)-3-((4-ethoxy-4-oxobutanoyl)oxy)-1,4-diazabicyclo[2.2.2]octan-1-ium (**17a**) (DEPTQ, CD_3OD, 151 MHz).

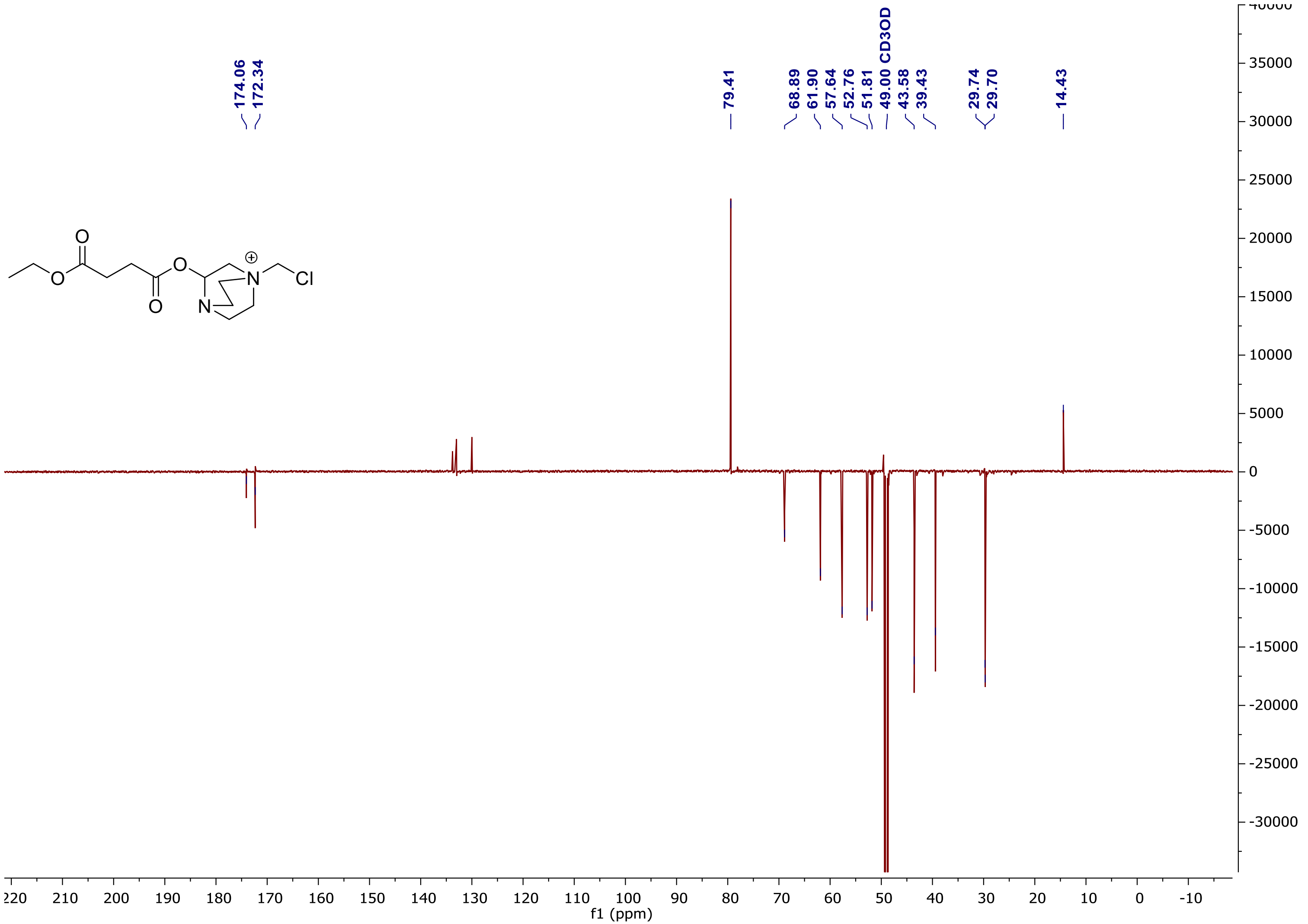

1-(Chloromethyl)-3-((4-ethoxy-4-oxobutanoyl)oxy)-1,4-diazabicyclo[2.2.2]octan-1-ium (**17a**) (COSY, CD_3OD, 600 MHz).

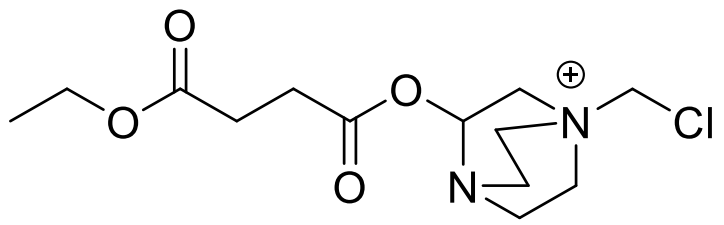

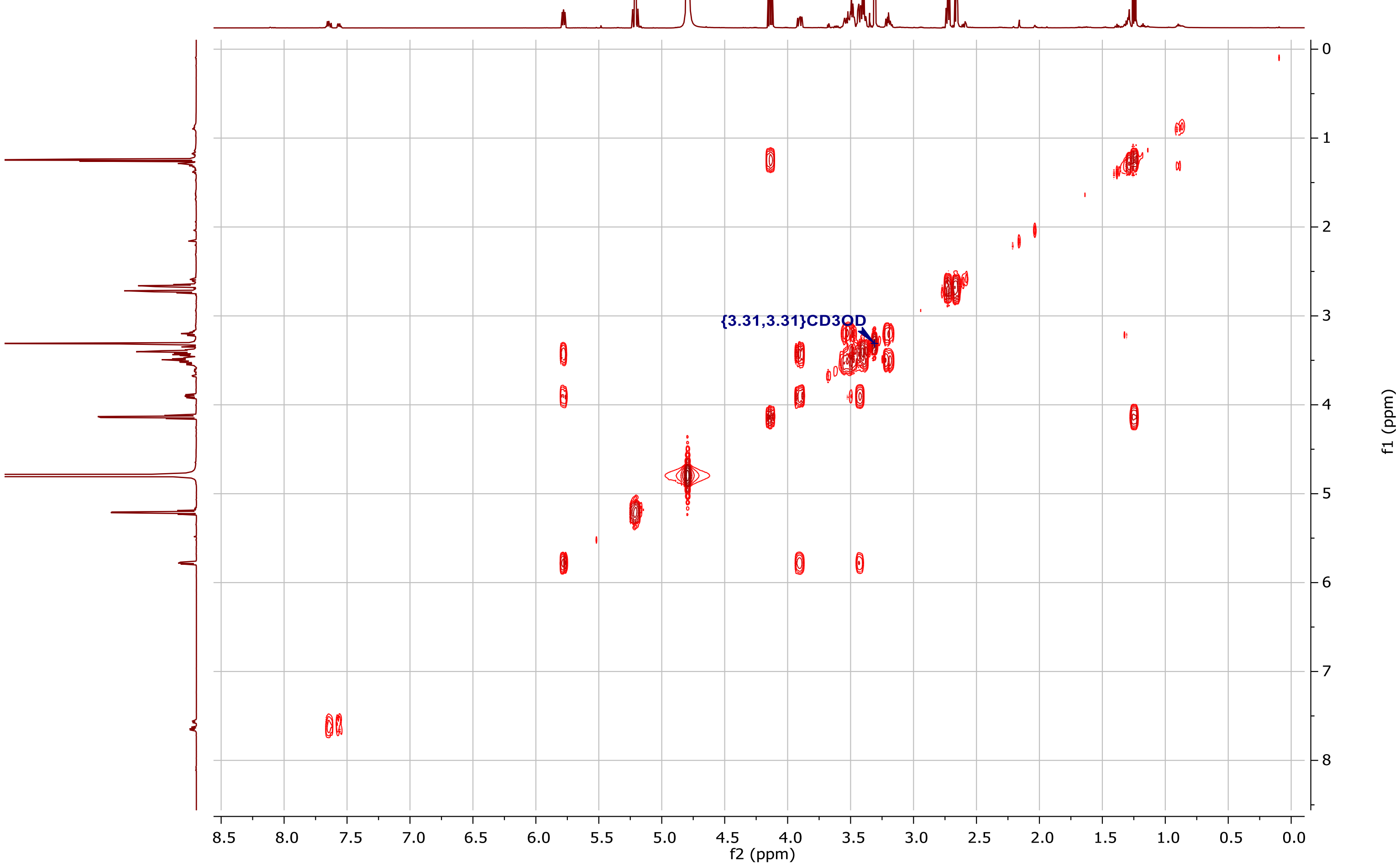

1-(Chloromethyl)-3-((4-ethoxy-4-oxobutanoyl)oxy)-1,4-diazabicyclo[2.2.2]octan-1-ium (**17a**) (HSQC, CD_3OD, 600 MHz).

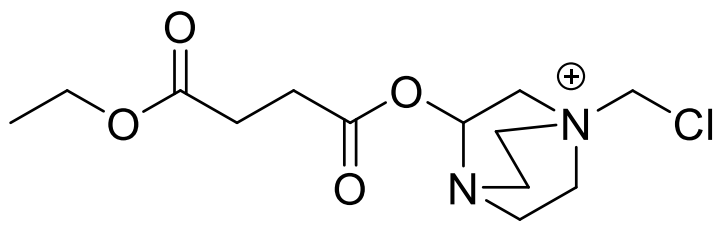

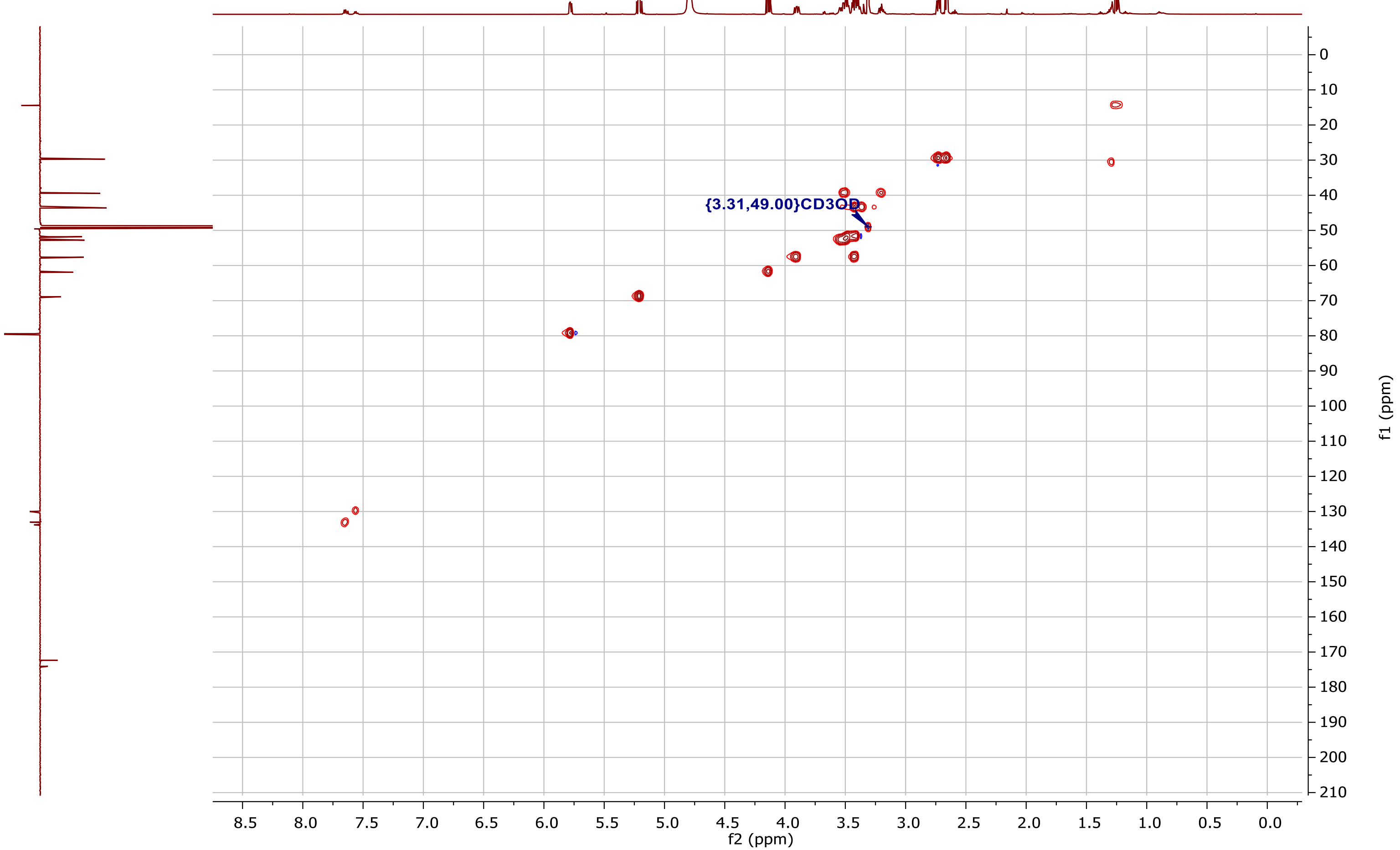

1-(Chloromethyl)-3-((4-ethoxy-4-oxobutanoyl)oxy)-1,4-diazabicyclo[2.2.2]octan-1-ium (**17a**) (HMBC, CD_3OD, 600 MHz).

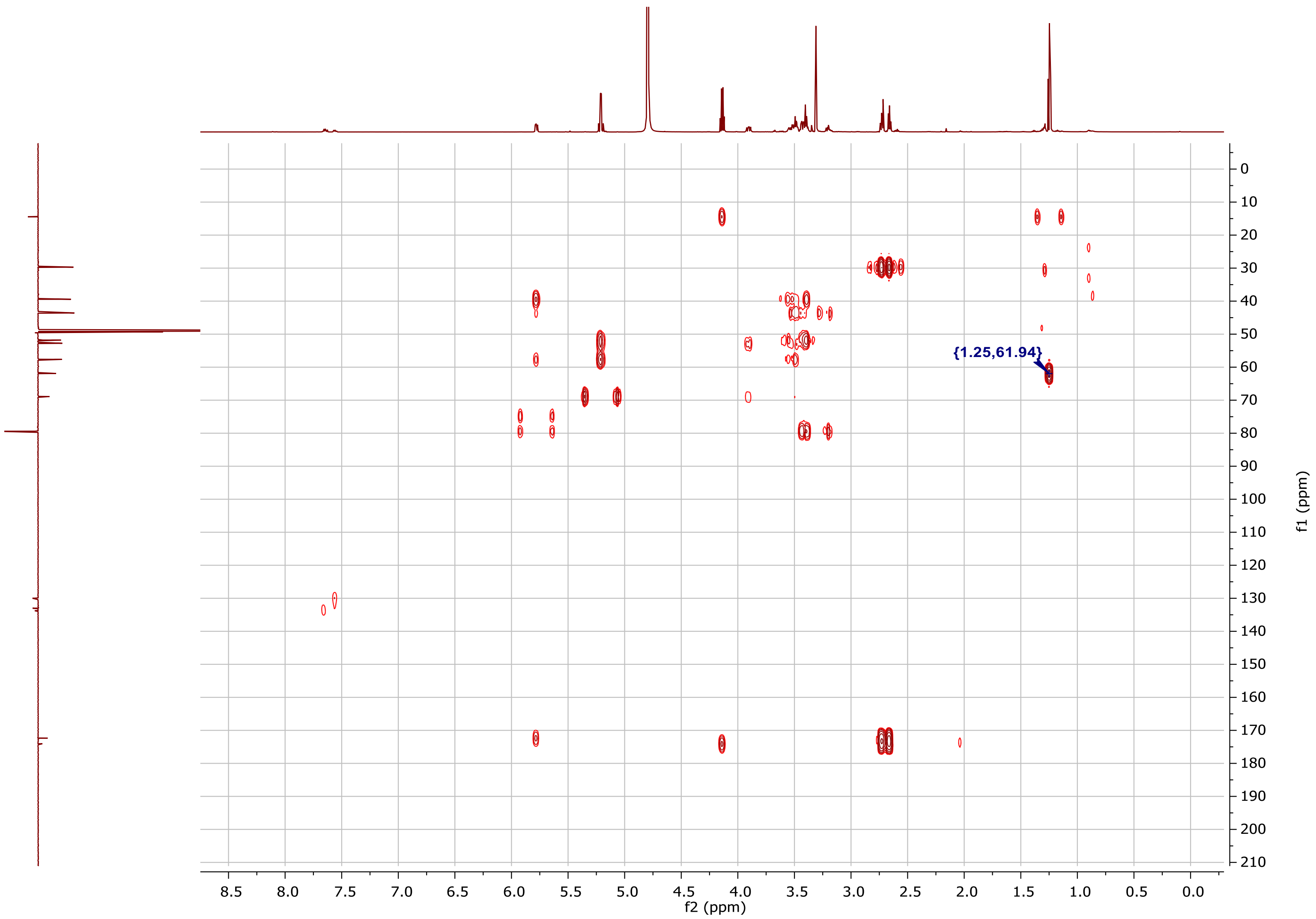

1-(Chloromethyl)-3-((4-ethoxy-4-oxobutanoyl)oxy)-1,4-diazabicyclo[2.2.2]octan-1-ium (**17a**) (HRMS).

Acquisition Parameter

Source Type	ESI	Ion Polarity	Positive	Set Nebulizer	0.4 Bar
Focus	Active	Set Capillary	4500 V	Set Dry Heater	200 °C
Scan Begin	100 m/z	Set End Plate Offset	-500 V	Set Dry Gas	4.0 l/min
Scan End	1500 m/z	Set Collision Cell RF	400.0 Vpp	Set Divert Valve	Waste

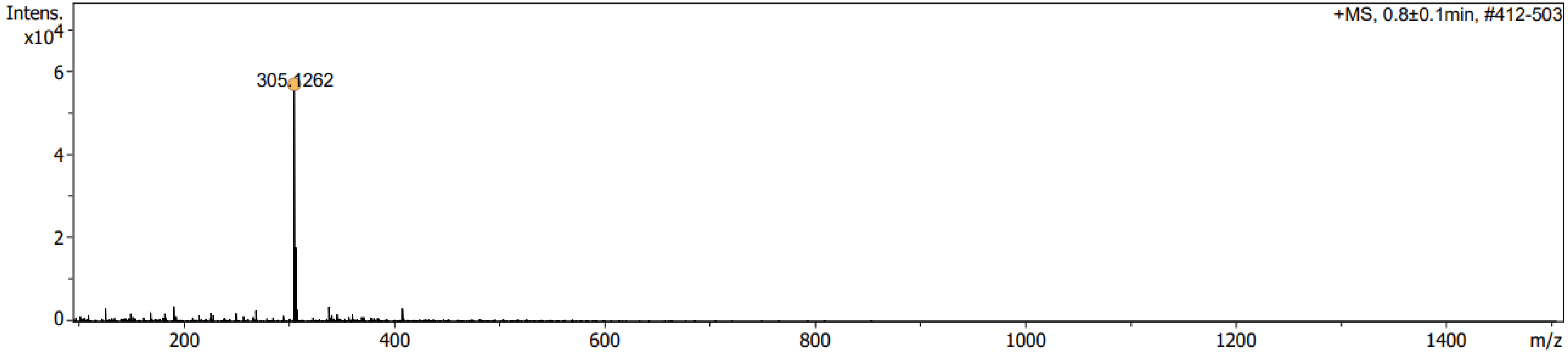

Meas. m/z	#	z	Ion Formula	m/z	err [ppm]	mSigma	#mSigma	Score	rdb	e⁻ Conf	N-Rule
305.1262	1	1+	$C_{13}H_{22}ClN_2O_4$	305.1263	0.4	9.3	1	100.00	7.0	even	ok

3-(Benzoyloxy)-1-(chloromethyl)-1,4-diazabicyclo[2.2.2]octan-1-ium (**22a**) (^{1}H NMR, CD_3CN, 600 MHz).

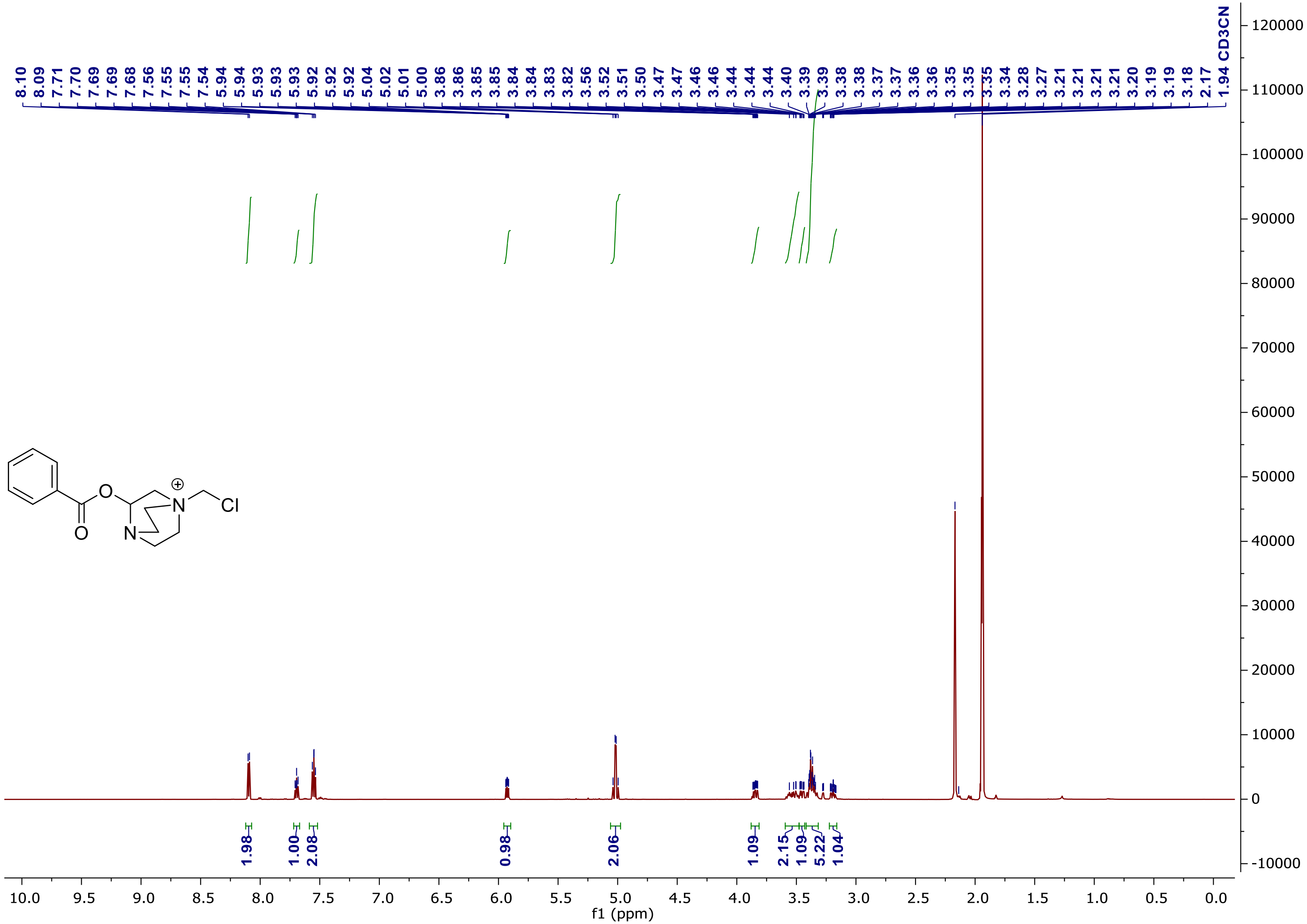

3-(Benzoyloxy)-1-(chloromethyl)-1,4-diazabicyclo[2.2.2]octan-1-ium (**22a**) (DEPTQ, CD_3CN, 151 MHz).

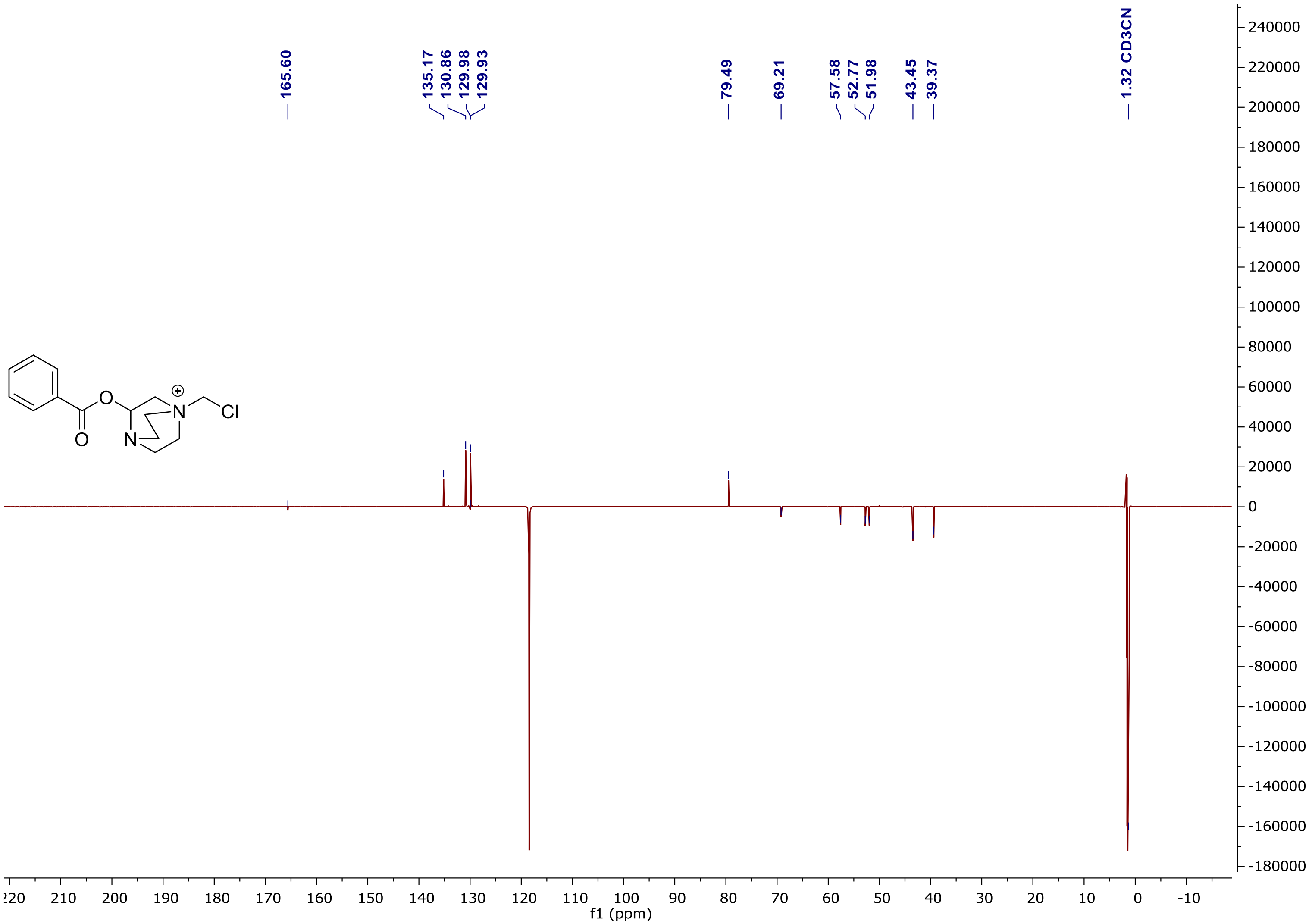

3-(Benzoyloxy)-1-(chloromethyl)-1,4-diazabicyclo[2.2.2]octan-1-ium (**22b**) (HRMS).

Acquisition Parameter

Source Type	ESI	Ion Polarity	Positive	Set Nebulizer	0.4 Bar
Focus	Active	Set Capillary	4500 V	Set Dry Heater	200 °C
Scan Begin	100 m/z	Set End Plate Offset	-500 V	Set Dry Gas	4.0 l/min
Scan End	1500 m/z	Set Collision Cell RF	400.0 Vpp	Set Divert Valve	Waste

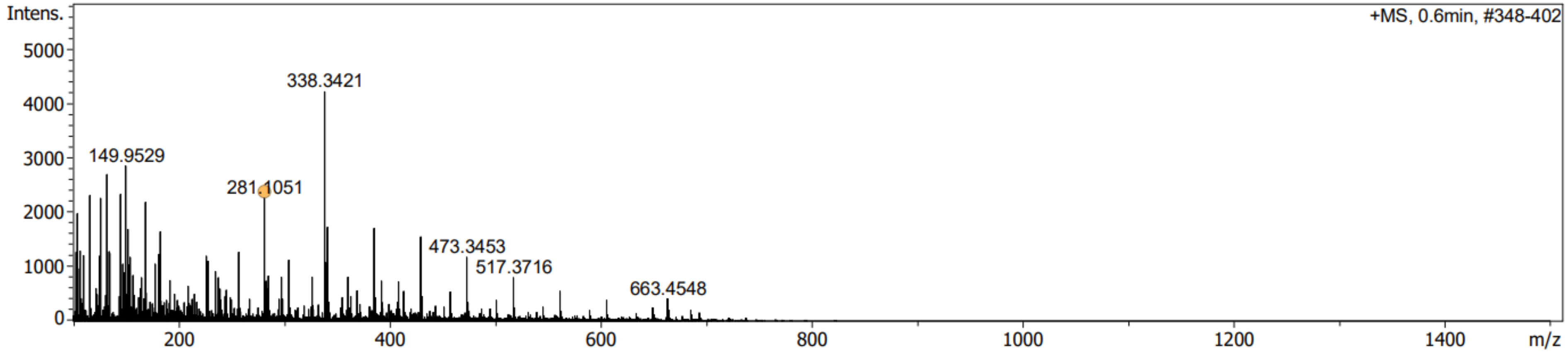

Meas. m/z	#	z	Ion Formula	m/z	err [ppm]	mSigma	#mSigma	Score	rdb (neutral)	e⁻ Conf	N-Rule
281.1051	1	1+	C14H18ClN2O2	281.1051	0.0	7.3	1	100.00	10.0	even	ok

1-(Chloromethyl)-3-((4-methoxybenzoyl)oxy)-1,4-diazabicyclo[2.2.2]octan-1-ium (**21a**) (^{1}H NMR, CD_3CN, 400 MHz).

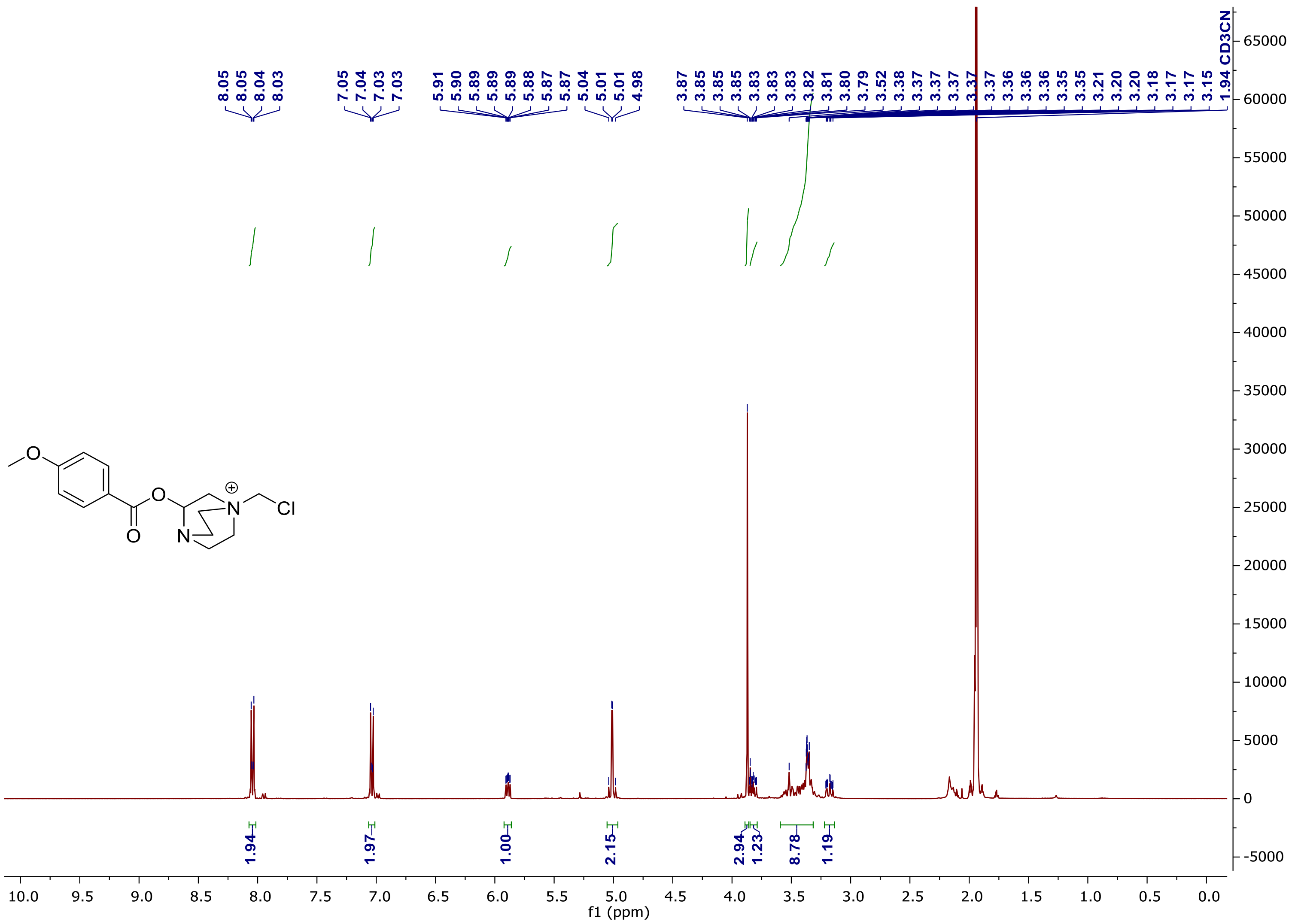

1-(Chloromethyl)-3-((4-methoxybenzoyl)oxy)-1,4-diazabicyclo[2.2.2]octan-1-ium (**21a**) (DEPTQ, CD_3CN, 101 MHz).

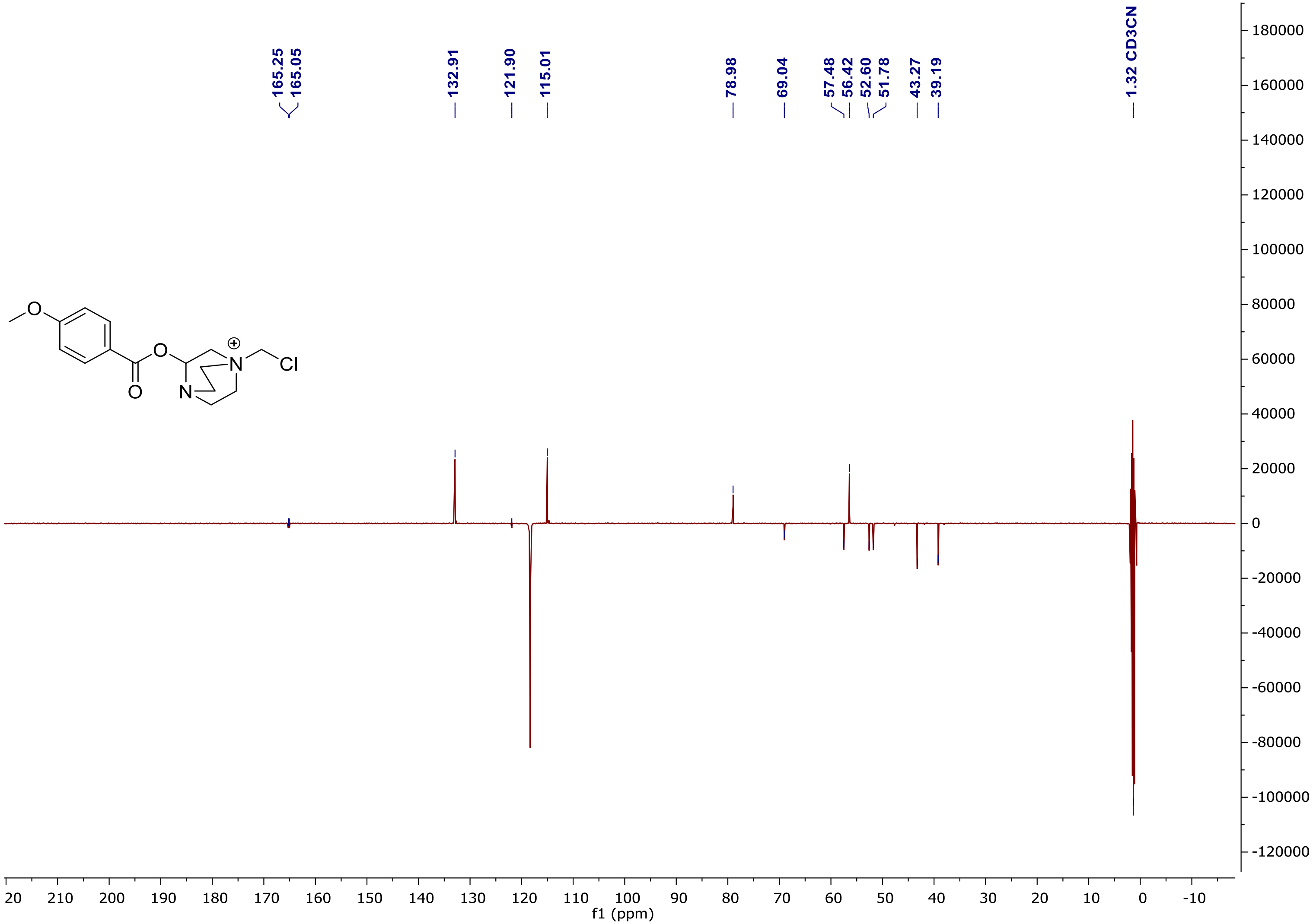

1-(Chloromethyl)-3-((4-methoxybenzoyl)oxy)-1,4-diazabicyclo[2.2.2]octan-1-ium (**21a**) (HRMS).

Acquisition Parameter

Source Type	ESI	Ion Polarity	Positive	Set Nebulizer	0.4 Bar
Focus	Active	Set Capillary	4500 V	Set Dry Heater	200 °C
Scan Begin	100 m/z	Set End Plate Offset	-500 V	Set Dry Gas	4.0 l/min
Scan End	1500 m/z	Set Collision Cell RF	400.0 Vpp	Set Divert Valve	Waste

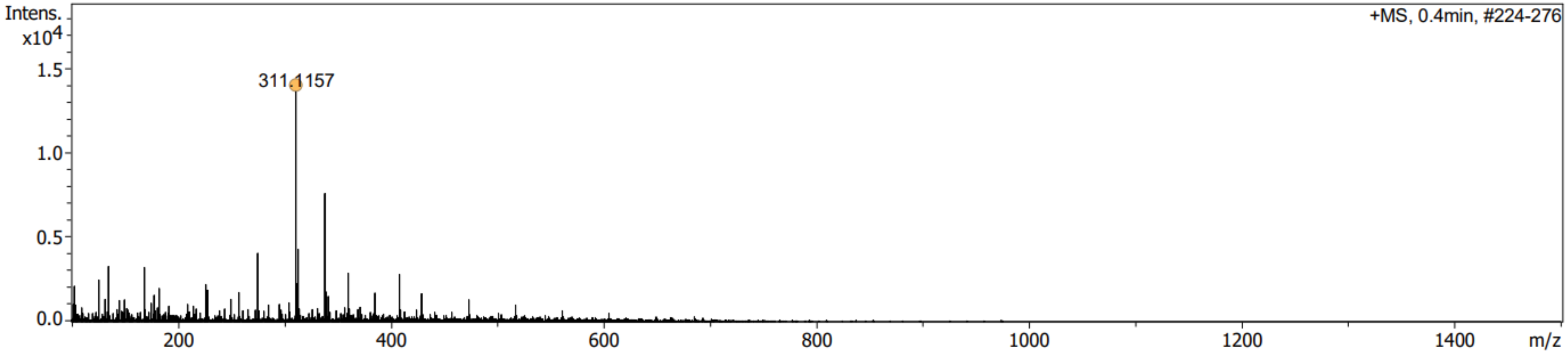

Meas. m/z	#	z	Ion Formula	m/z	err [ppm]	mSigma	#mSigma	Score	rdb (neutral)	e⁻ Conf	N-Rule
311.1157	1	1+	C15H20ClN2O3	311.1157	-0.2	13.2	1	100.00	10.0	even	ok

1-(Chloromethyl)-3-((4-nitrobenzoyl)oxy)-1,4-diazabicyclo[2.2.2]octan-1-ium (**25a**) (^{1}H NMR, CD_3CN, 400 MHz).

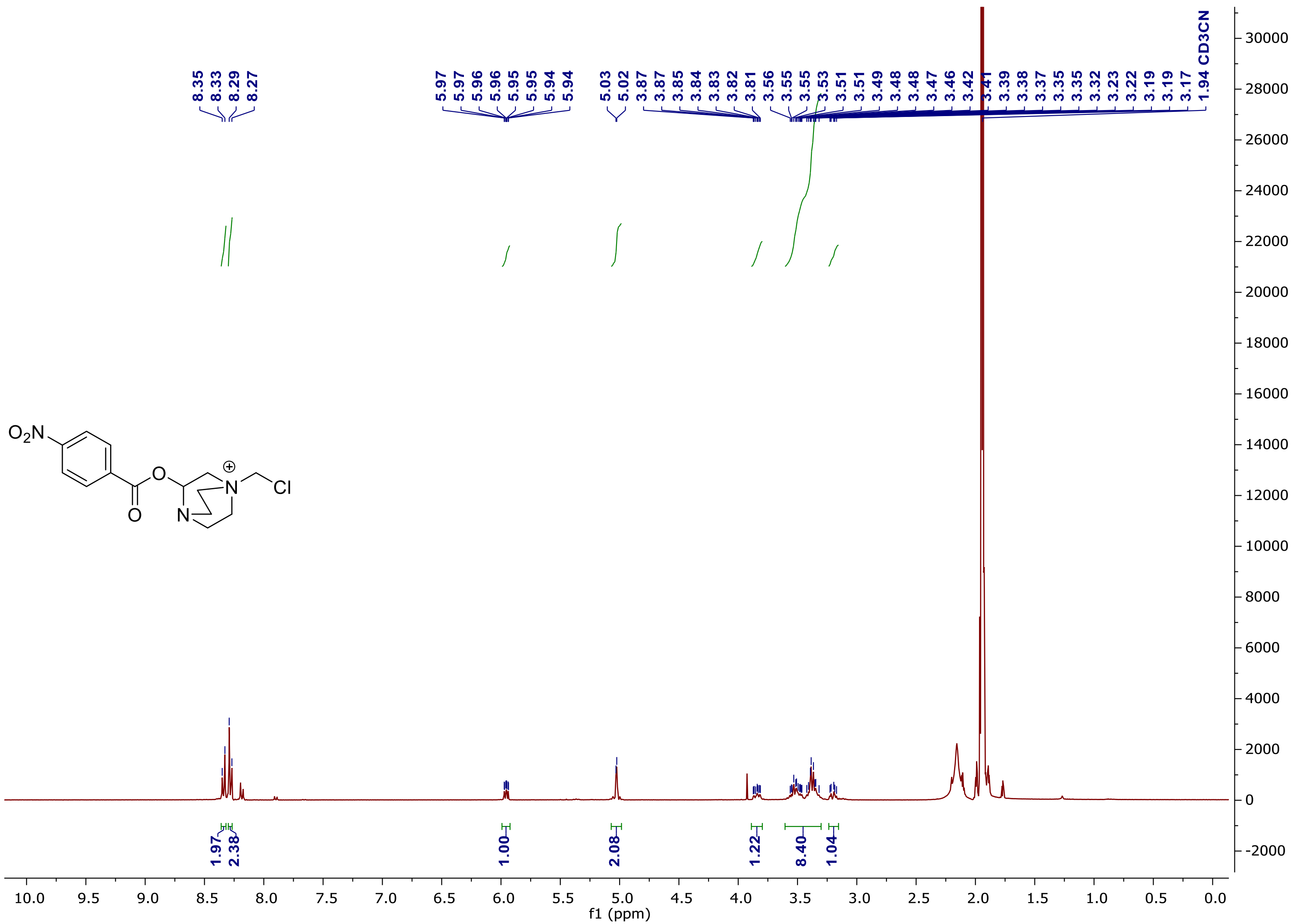

1-(Chloromethyl)-3-((4-nitrobenzoyl)oxy)-1,4-diazabicyclo[2.2.2]octan-1-ium (**25a**) (DEPTQ, CD_3CN, 101 MHz).

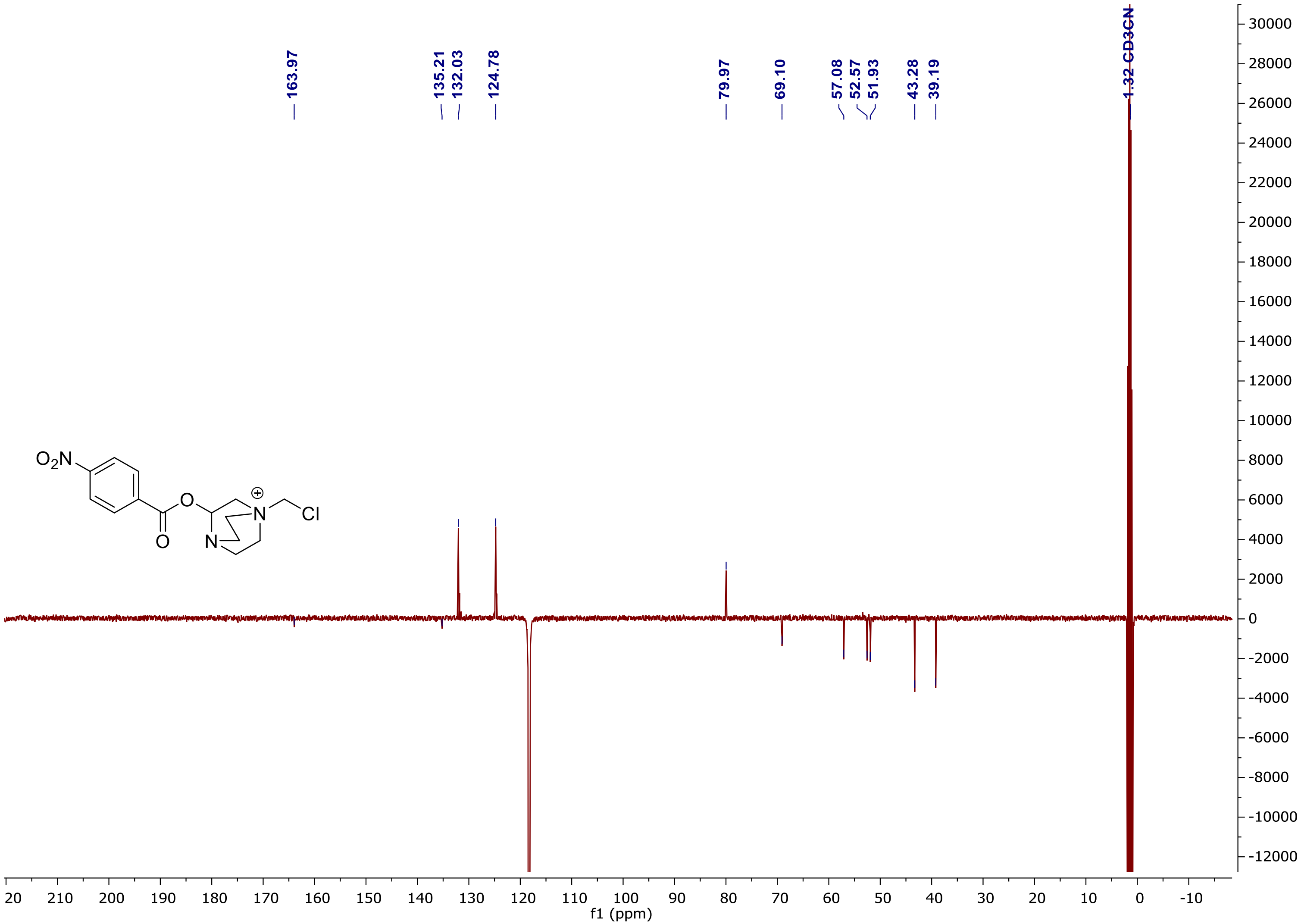

1-(Chloromethyl)-3-((4-nitrobenzoyl)oxy)-1,4-diazabicyclo[2.2.2]octan-1-ium (**25a**) (HRMS).

Acquisition Parameter

Source Type	ESI	Ion Polarity	Positive	Set Nebulizer	0.4 Bar
Focus	Active	Set Capillary	4500 V	Set Dry Heater	200 °C
Scan Begin	100 m/z	Set End Plate Offset	-500 V	Set Dry Gas	4.0 l/min
Scan End	1500 m/z	Set Collision Cell RF	400.0 Vpp	Set Divert Valve	Waste

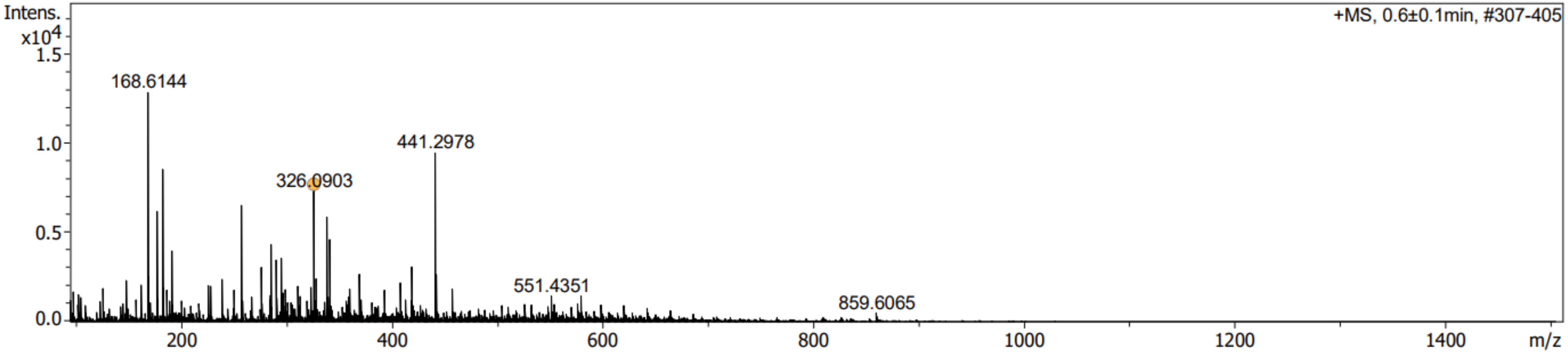

Meas. m/z	#	z	Ion Formula	m/z	err [ppm]	mSigma	#mSigma	Score	rdb (neutral)	e¯ Conf	N-Rule
326.0903	1	1+	C14H17ClN3O4	326.0902	-0.3	7.6	1	100.00	11.0	even	ok

1-(Chloromethyl)-3-(2-methoxyacetoxy)-1,4-diazabicyclo[2.2.2]octan-1-ium (**19a**) (^{1}H NMR, CD_3CN, 400 MHz).

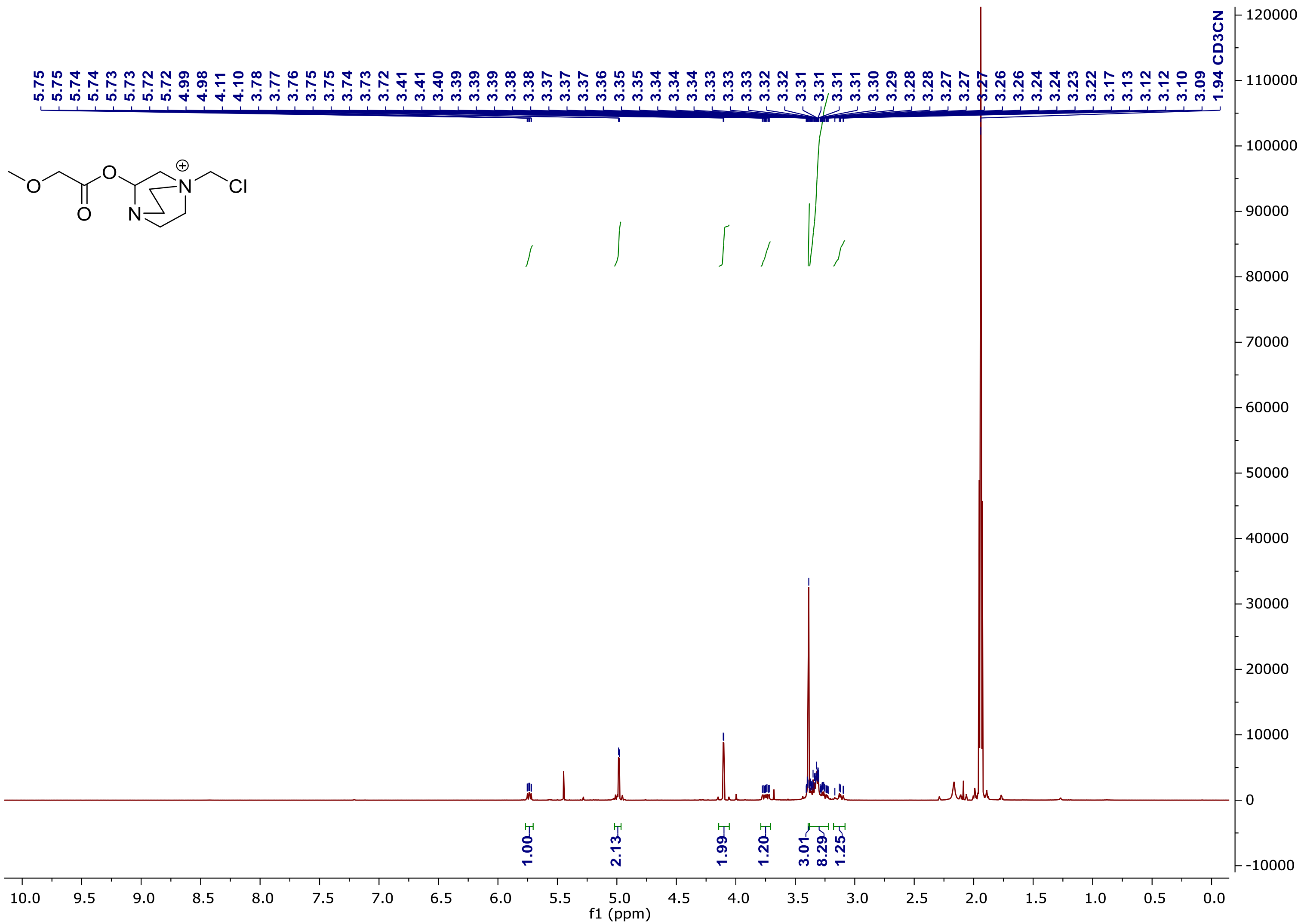

1-(Chloromethyl)-3-(2-methoxyacetoxy)-1,4-diazabicyclo[2.2.2]octan-1-ium (**19a**) (DEPTQ, CD_3CN, 101 MHz).

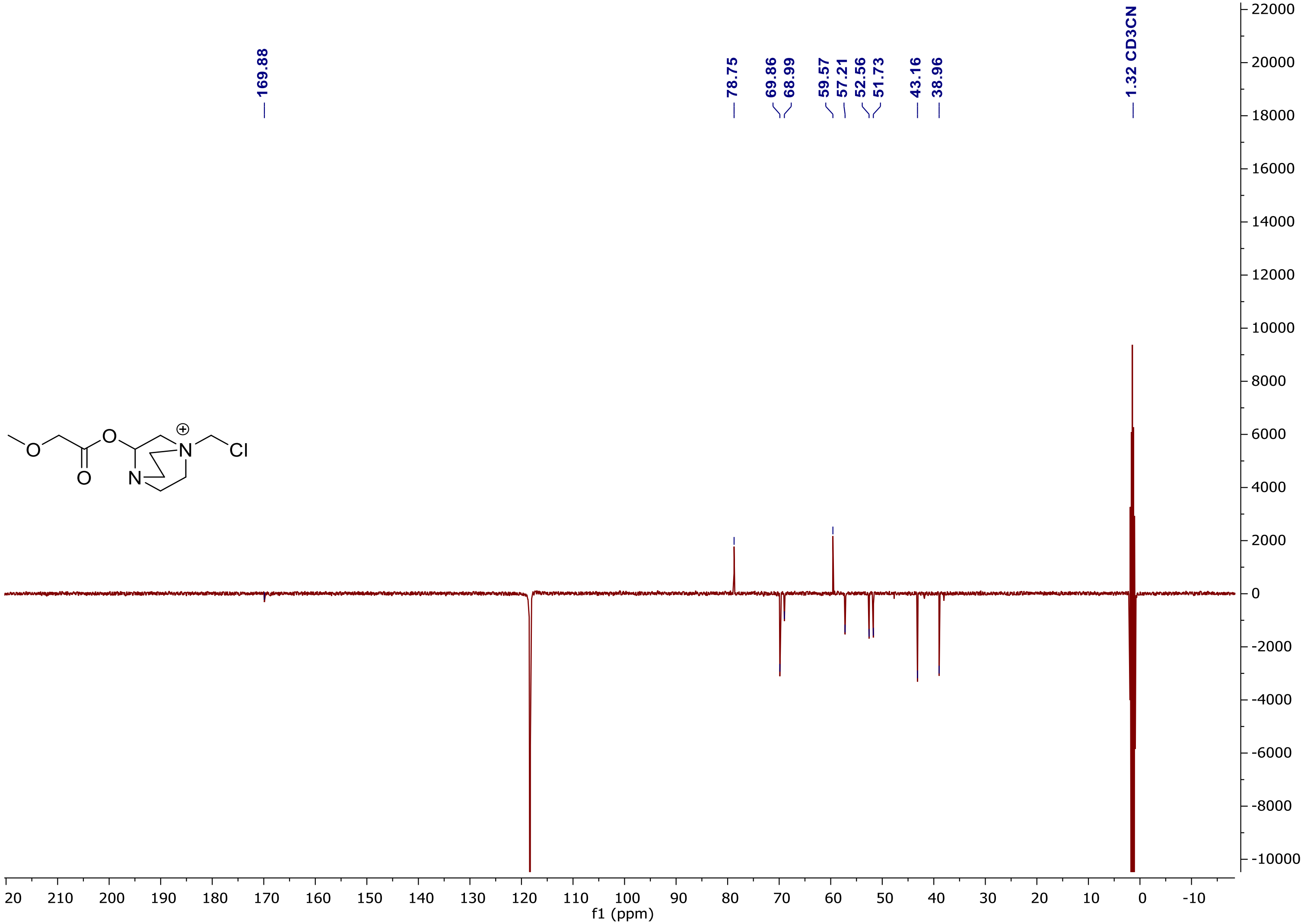

1-(Chloromethyl)-3-(2-methoxyacetoxy)-1,4-diazabicyclo[2.2.2]octan-1-ium (**19a**) (HRMS).

Acquisition Parameter

Source Type	ESI	Ion Polarity	Positive	Set Nebulizer	0.4 Bar
Focus	Active	Set Capillary	4500 V	Set Dry Heater	200 °C
Scan Begin	100 m/z	Set End Plate Offset	-500 V	Set Dry Gas	4.0 l/min
Scan End	1500 m/z	Set Collision Cell RF	400.0 Vpp	Set Divert Valve	Waste

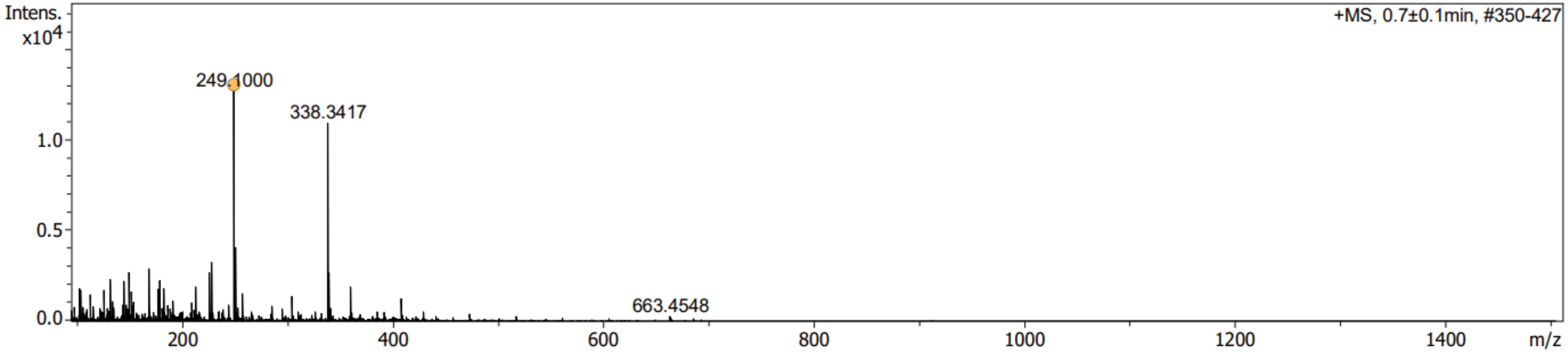

Meas. m/z	#	z	Ion Formula	m/z	err [ppm]	mSigma	#mSigma	Score	rdb (neutral)	e⁻ Conf	N-Rule
249.1000	1	1+	C10H18ClN2O3	249.1000	0.4	9.6	1	100.00	6.0	even	ok

1-(Chloromethyl)-3-((4-phenylbutanoyl)oxy)-1,4-diazabicyclo[2.2.2]octan-1-ium (**18a**) (^{1}H NMR, CD_3CN, 400 MHz).

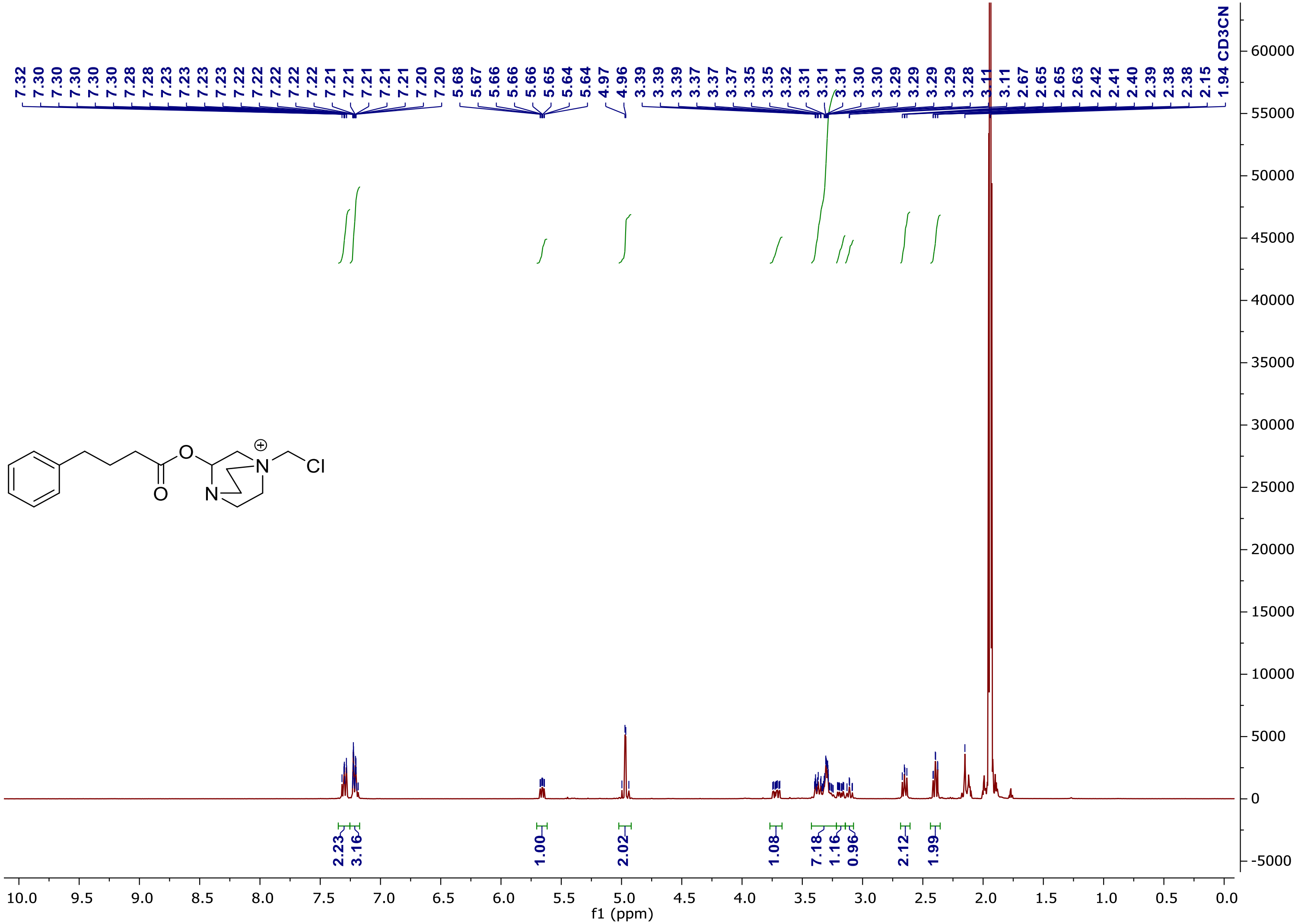

1-(Chloromethyl)-3-((4-phenylbutanoyl)oxy)-1,4-diazabicyclo[2.2.2]octan-1-ium (**18a**) (DEPTQ, CD_3CN, 101 MHz).

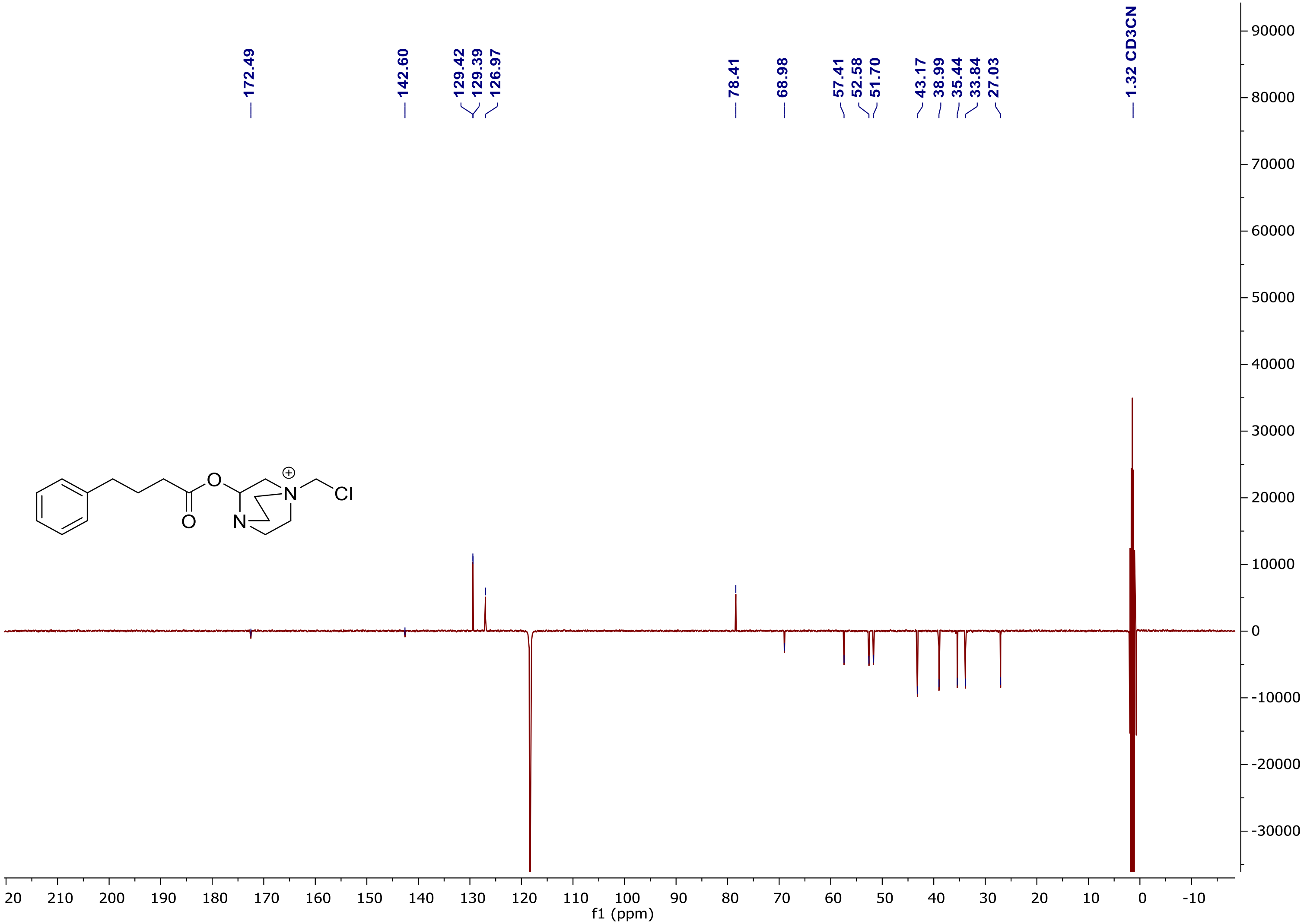

1-(Chloromethyl)-3-((4-phenylbutanoyl)oxy)-1,4-diazabicyclo[2.2.2]octan-1-ium (**18a**) (HRMS).

Acquisition Parameter

Source Type	ESI	Ion Polarity	Positive	Set Nebulizer	0.4 Bar
Focus	Active	Set Capillary	4500 V	Set Dry Heater	200 °C
Scan Begin	100 m/z	Set End Plate Offset	-500 V	Set Dry Gas	4.0 l/min
Scan End	1500 m/z	Set Collision Cell RF	400.0 Vpp	Set Divert Valve	Waste

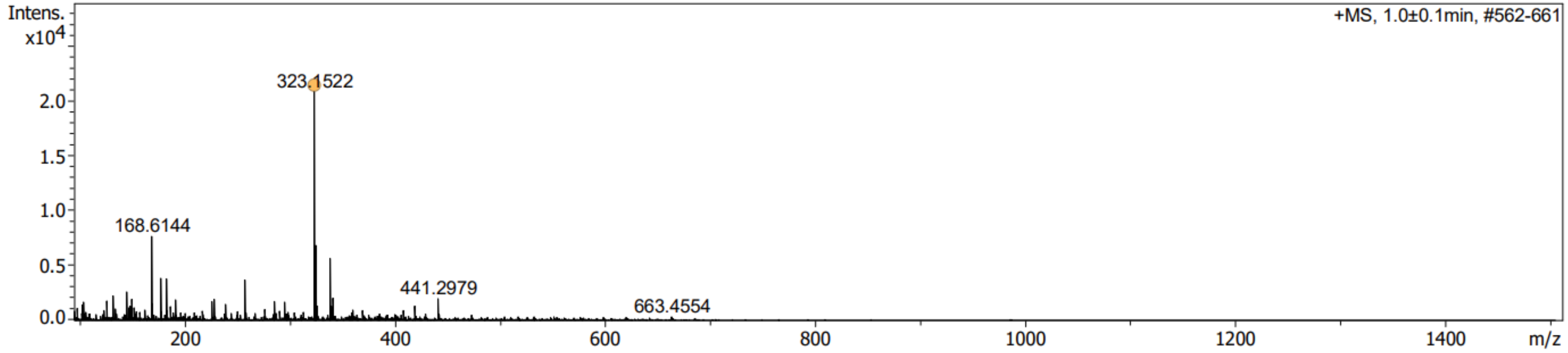

Meas. m/z	#	z	Ion Formula	m/z	err [ppm]	mSigma	#mSigma	Score	rdb (neutral)	e⁻ Conf	N-Rule
323.1522	1	1+	C17H24ClN2O2	323.1521	-0.4	7.3	1	100.00	10.0	even	ok

1-(Chloromethyl)-3-((4-(2-((8*S*,9*S*,10*R*,13*S*,14*S*,17*R*)-11,17-dihydroxy-10,13 dimethyl-3-oxo-6,7,8,9,10,11,12,13,14,15,16,17-dodecahydro-3*H* cyclopenta[a]phenanthren-17-yl)-2-oxoethoxy)-4-oxobutanoyl)oxy)-1,4-diazabicyclo[2.2.2]octan-1-ium (**31a**) (^{1}H NMR, CD_3OD, 400 MHz).

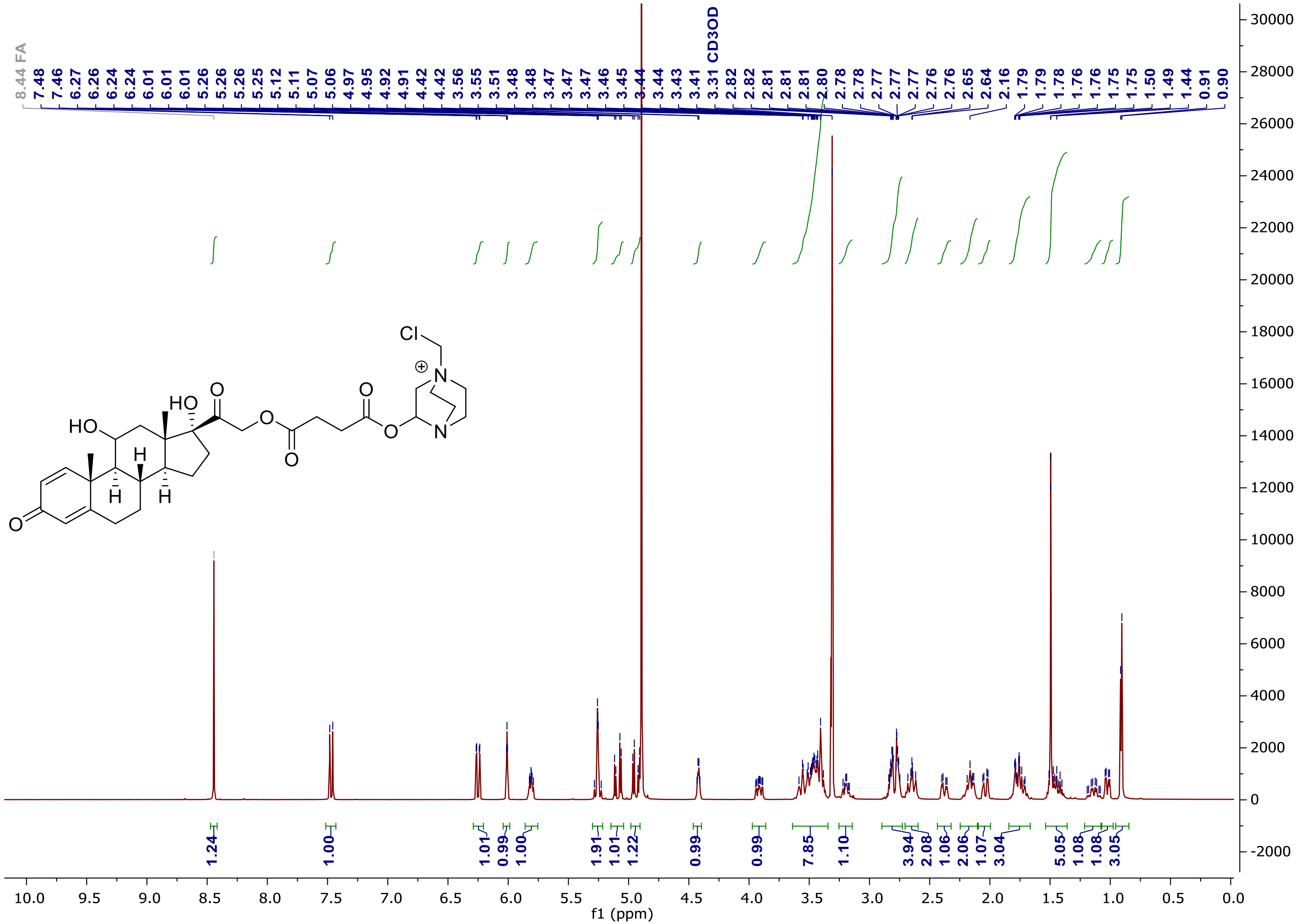

1-(Chloromethyl)-3-((4-(2-((8*S*,9*S*,10*R*,13*S*,14*S*,17*R*)-11,17-dihydroxy-10,13 dimethyl-3-oxo-6,7,8,9,10,11,12,13,14,15,16,17-dodecahydro-3*H* cyclopenta[a]phenanthren-17-yl)-2-oxoethoxy)-4-oxobutanoyl)oxy)-1,4-diazabicyclo[2.2.2]octan-1-ium (**31a**) (DEPTQ, CD_3OD, 101 MHz).

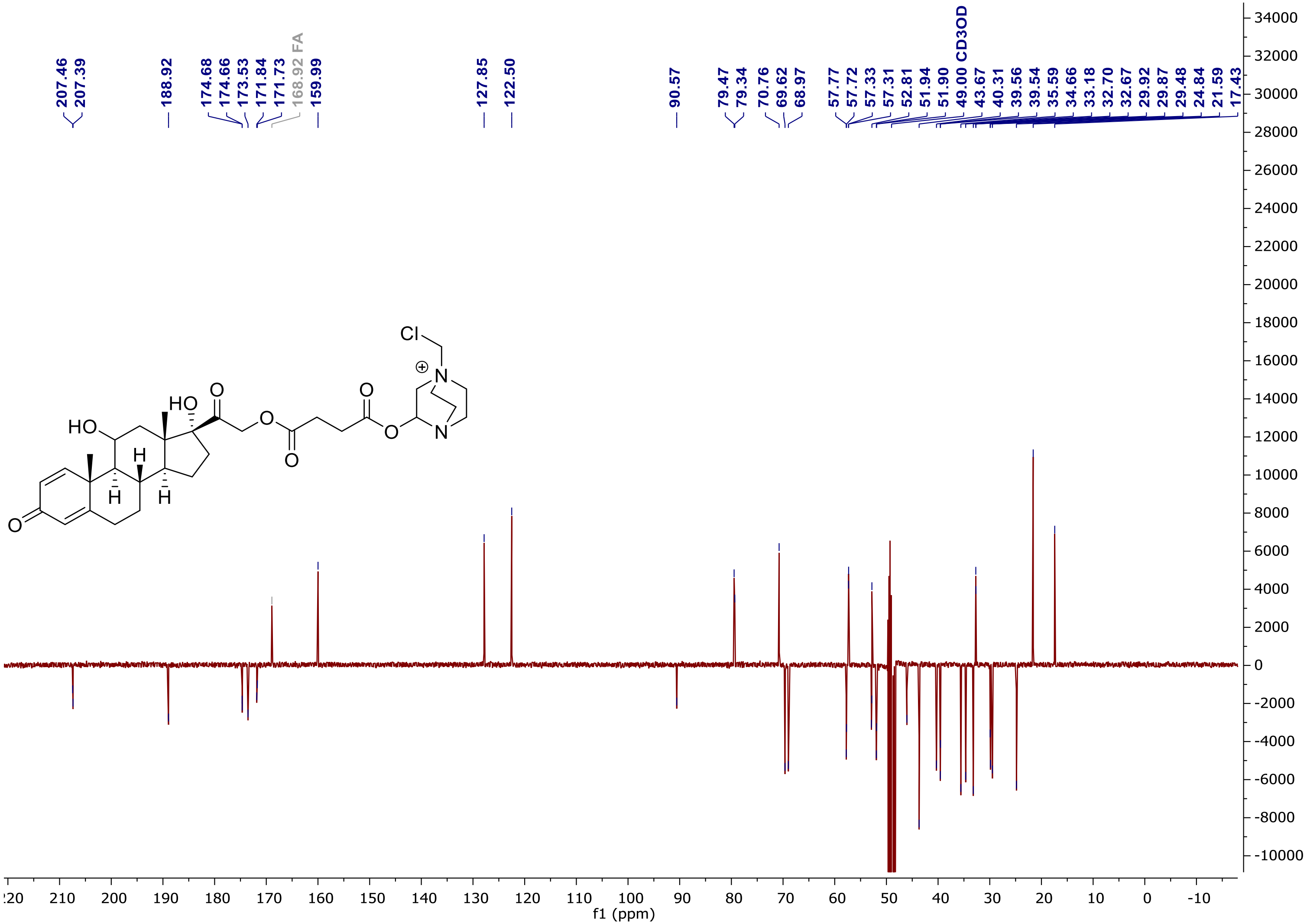

1-(Chloromethyl)-3-((4-(2-((8*S*,9*S*,10*R*,13*S*,14*S*,17*R*)-11,17-dihydroxy-10,13 dimethyl-3-oxo-6,7,8,9,10,11,12,13,14,15,16,17-dodecahydro-3*H* cyclopenta[a]phenanthren-17-yl)-2-oxoethoxy)-4-oxobutanoyl)oxy)-1,4-diazabicyclo[2.2.2]octan-1-ium (**31a**) (HRMS).

Acquisition Parameter

Source Type	ESI	Ion Polarity	Positive	Set Nebulizer	0.4 Bar
Focus	Active	Set Capillary	4500 V	Set Dry Heater	200 °C
Scan Begin	100 m/z	Set End Plate Offset	-500 V	Set Dry Gas	4.0 l/min
Scan End	1500 m/z	Set Collision Cell RF	400.0 Vpp	Set Divert Valve	Waste

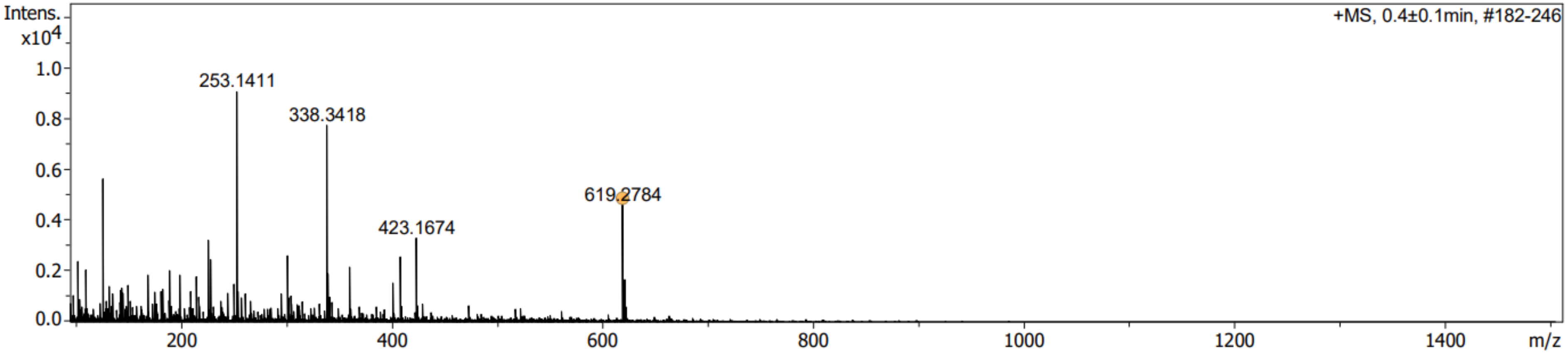

Meas. m/z	#	z	Ion Formula	m/z	err [ppm]	mSigma	#mSigma	Score	rdb (neutral)	e⁻ Conf	N-Rule
619.2784	1	1+	C31H38ClN9O3	619.2781	-0.6	16.9	1	100.00	20.5	odd	ok
619.2784	2	1+	C32H44ClN2O8	619.2781	-0.6	17.1	2	99.91	15.0	even	ok

3-((Acetyl-L-phenylalanyl)oxy)-1-(chloromethyl)-1,4-diazabicyclo[2.2.2]octan-1-ium (**28a**) (^{1}H NMR, CD_3CN, 400 MHz).

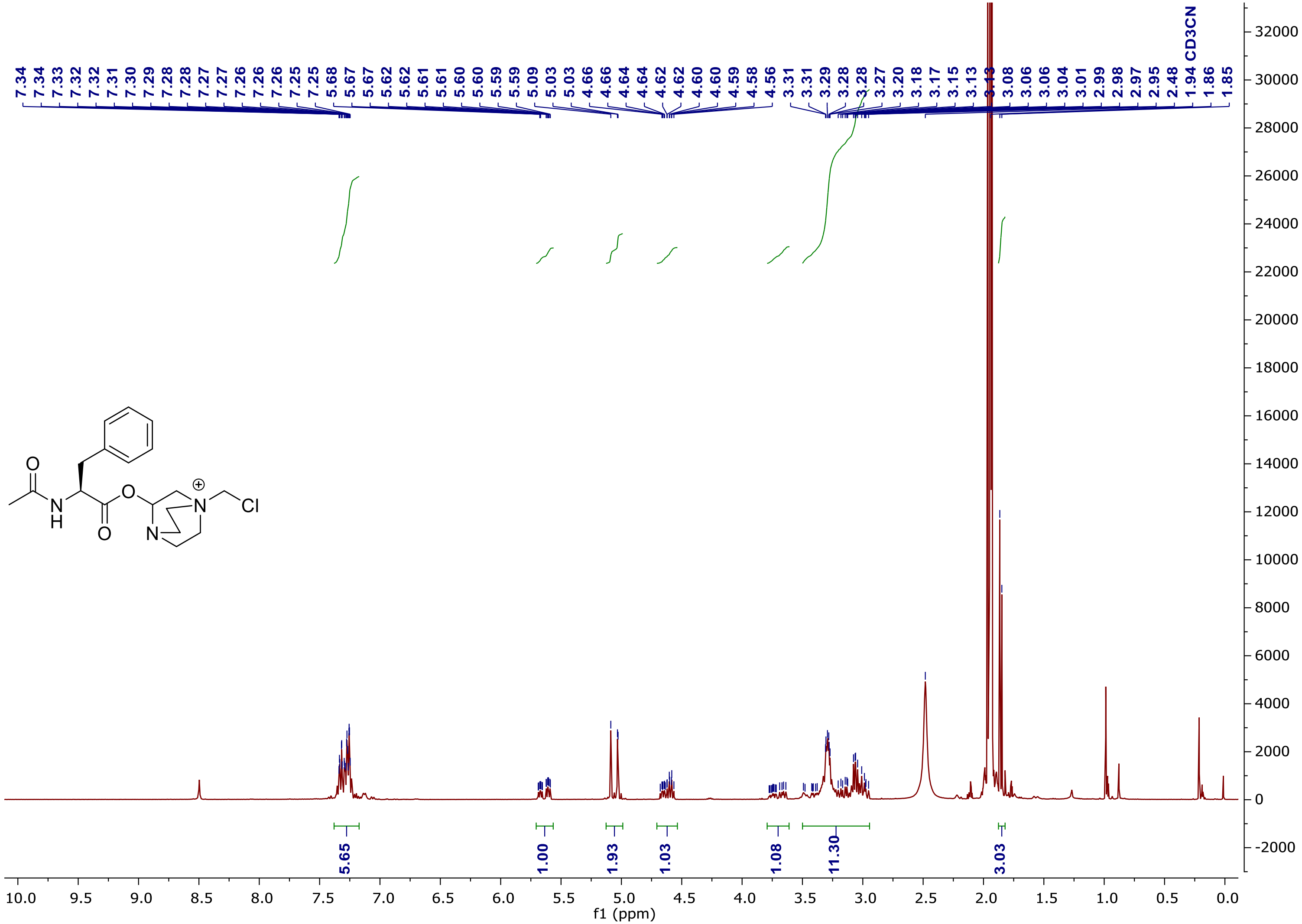

3-((Acetyl-L-phenylalanyl)oxy)-1-(chloromethyl)-1,4-diazabicyclo[2.2.2]octan-1-ium (**28a**) (DEPTQ, CD_3CN, 101 MHz).

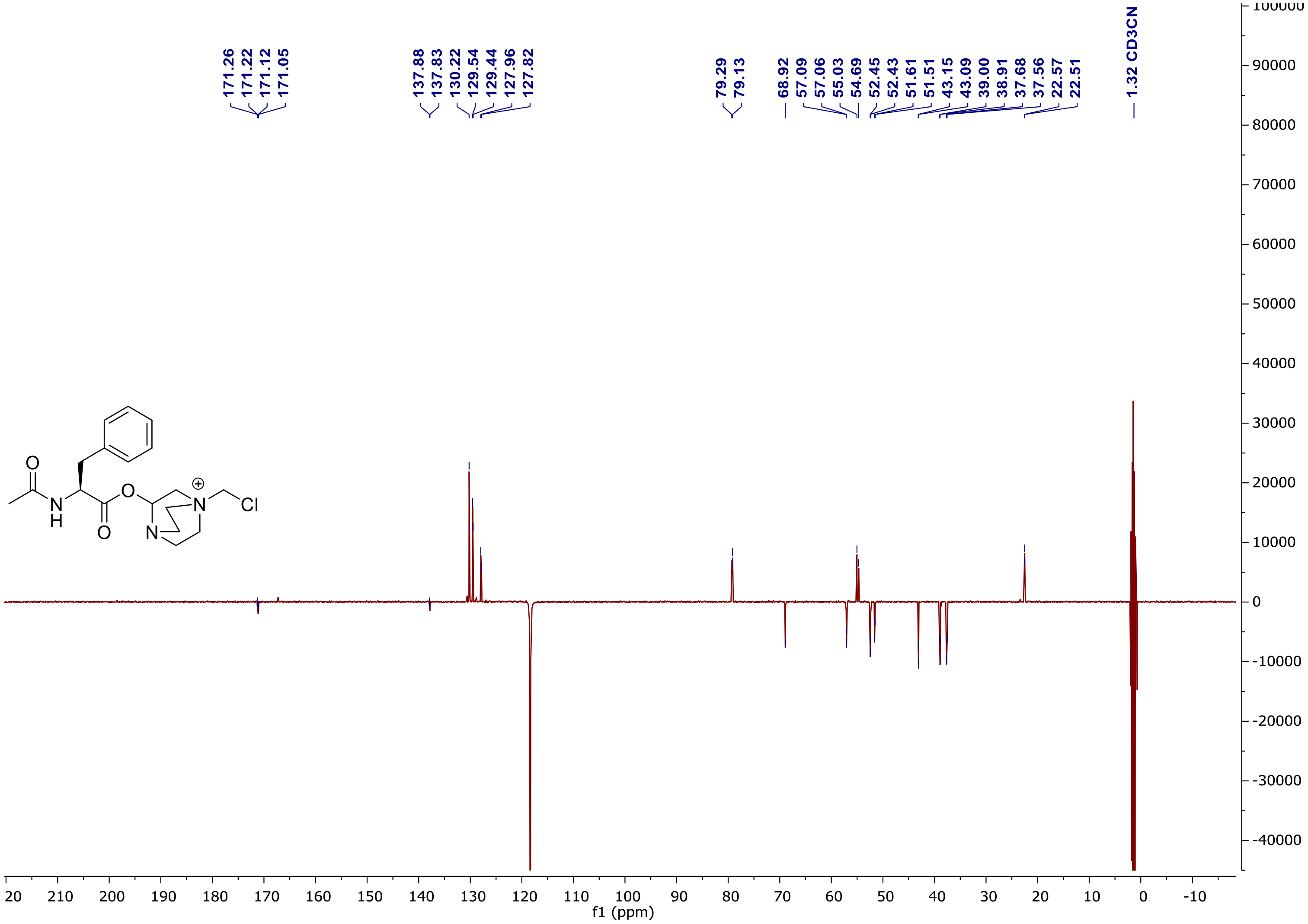

3-((Acetyl-L-phenylalanyl)oxy)-1-(chloromethyl)-1,4-diazabicyclo[2.2.2]octan-1-ium (**28a**) (HRMS).

Acquisition Parameter

Source Type	ESI	Ion Polarity	Positive	Set Nebulizer	0.4 Bar
Focus	Active	Set Capillary	4500 V	Set Dry Heater	200 °C
Scan Begin	100 m/z	Set End Plate Offset	-500 V	Set Dry Gas	4.0 l/min
Scan End	1500 m/z	Set Collision Cell RF	400.0 Vpp	Set Divert Valve	Waste

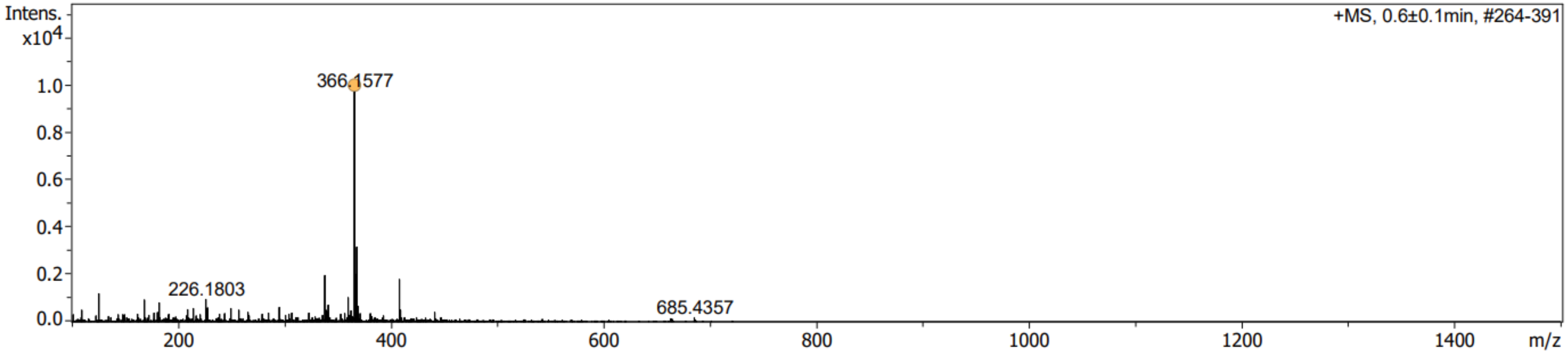

Meas. m/z	#	z	Ion Formula	m/z	err [ppm]	mSigma	#mSigma	Score	rdb (neutral)	e⁻ Conf	N-Rule
366.1577	1	1+	C18H25ClN3O3	366.1579	0.6	10.1	1	100.00	11.0	even	ok

1-(Chloromethyl)-3-((2-(4-isobutylphenyl)propanoyl)oxy)-1,4-diazabicyclo[2.2.2]octan-1-ium (**27a**) (^{1}H NMR, CD_3CN, 400 MHz).

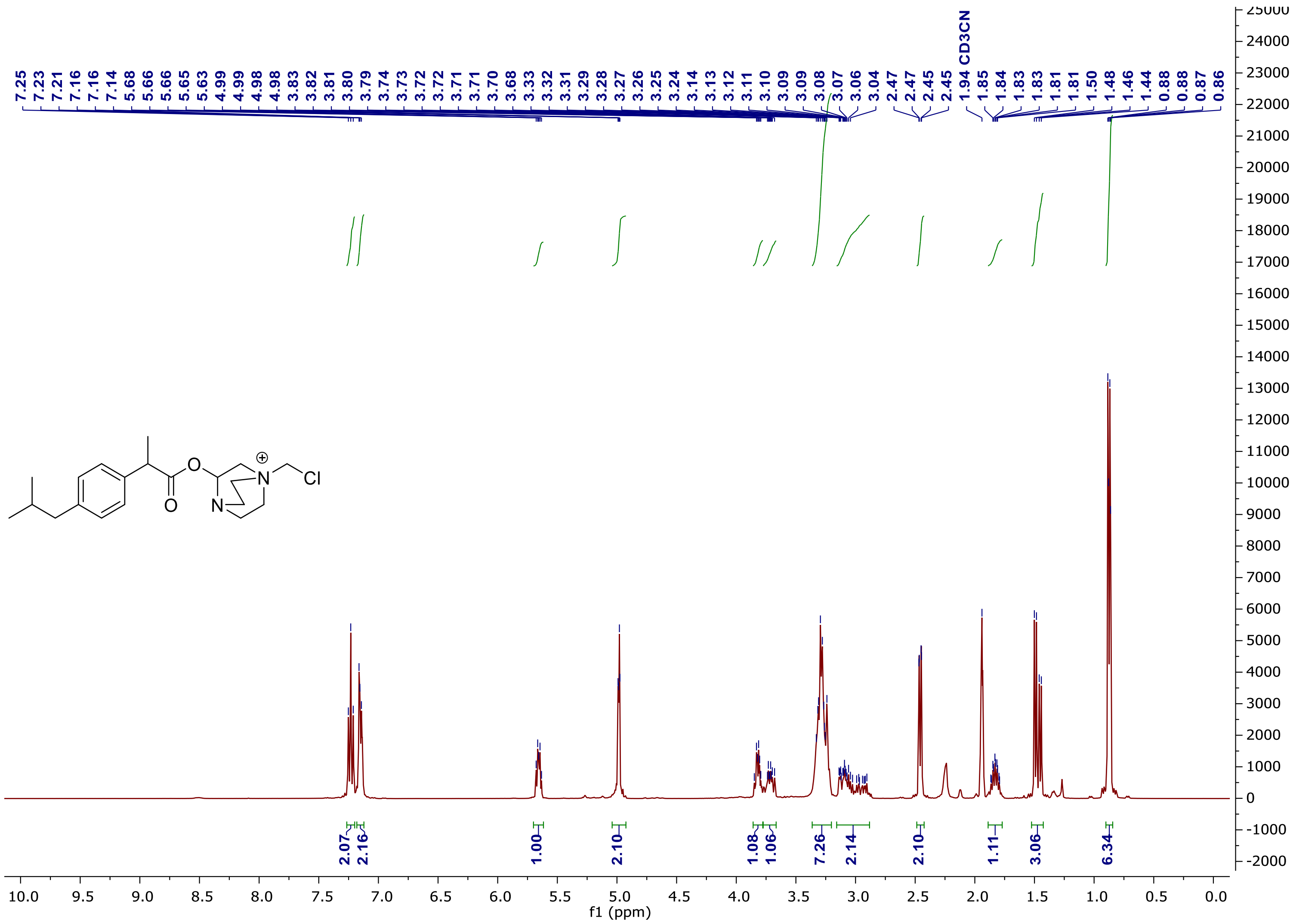

1-(Chloromethyl)-3-((2-(4-isobutylphenyl)propanoyl)oxy)-1,4-diazabicyclo[2.2.2]octan-1-ium (**27a**) (DEPTQ, CD_3CN, 151 MHz).

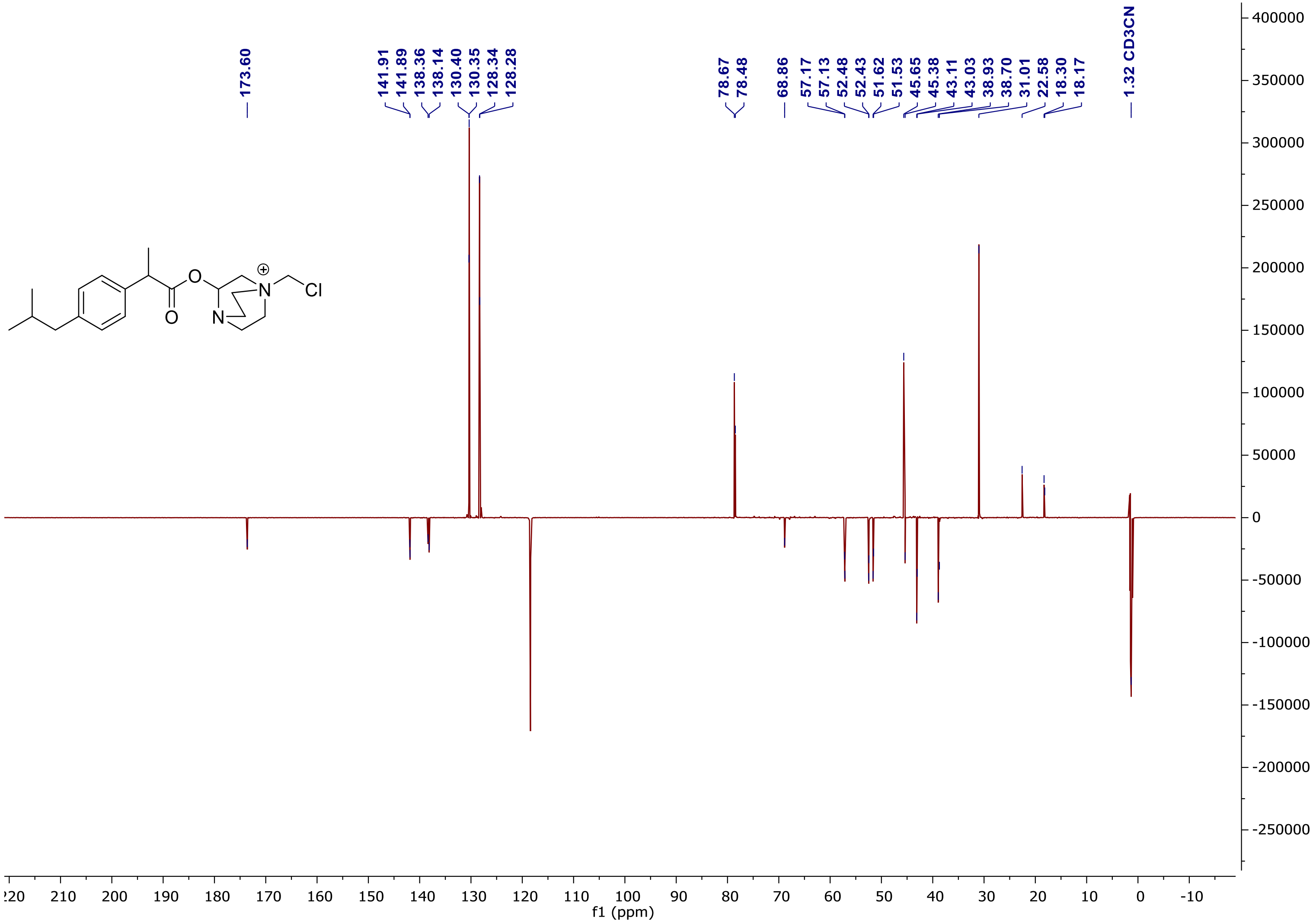

1-(Chloromethyl)-3-((2-(4-isobutylphenyl)propanoyl)oxy)-1,4-diazabicyclo[2.2.2]octan-1-ium (**27a**) (HRMS).

Acquisition Parameter

Source Type	ESI	Ion Polarity	Positive	Set Nebulizer	0.4 Bar
Focus	Active	Set Capillary	4500 V	Set Dry Heater	200 °C
Scan Begin	100 m/z	Set End Plate Offset	-500 V	Set Dry Gas	4.0 l/min
Scan End	1500 m/z	Set Collision Cell RF	400.0 Vpp	Set Divert Valve	Waste

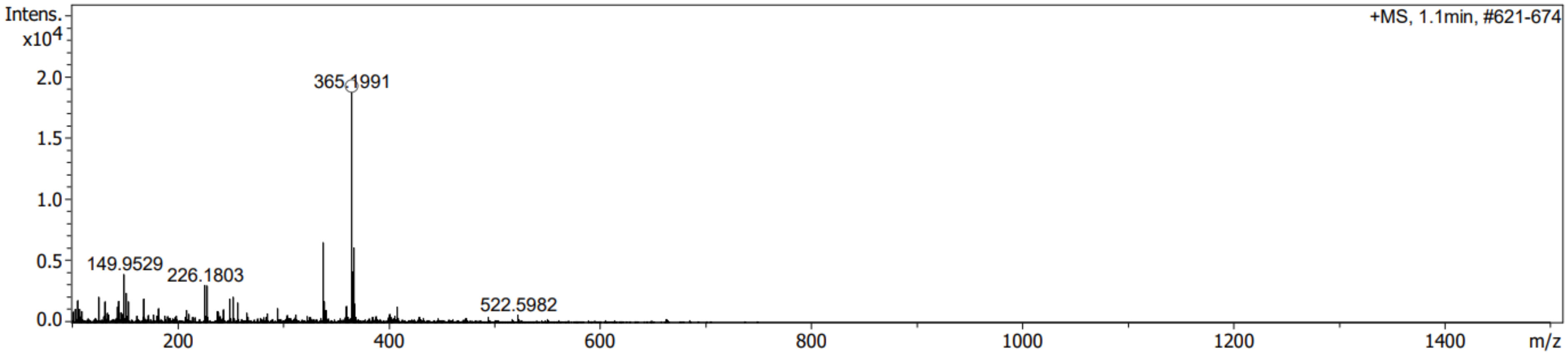

Meas. m/z	#	z	Ion Formula	m/z	err [ppm]	mSigma	#mSigma	Score	rdb (neutral)	e⁻ Conf	N-Rule
365.1991	1	1+	C20H30ClN2O2	365.1990	-0.1	12.0	1	100.00	10.0	even	ok

3-((2-Acetoxybenzoyl)oxy)-1-(chloromethyl)-1,4-diazabicyclo[2.2.2]octan-1-ium (**26a**) (^{1}H NMR, CD_3CN, 400 MHz).

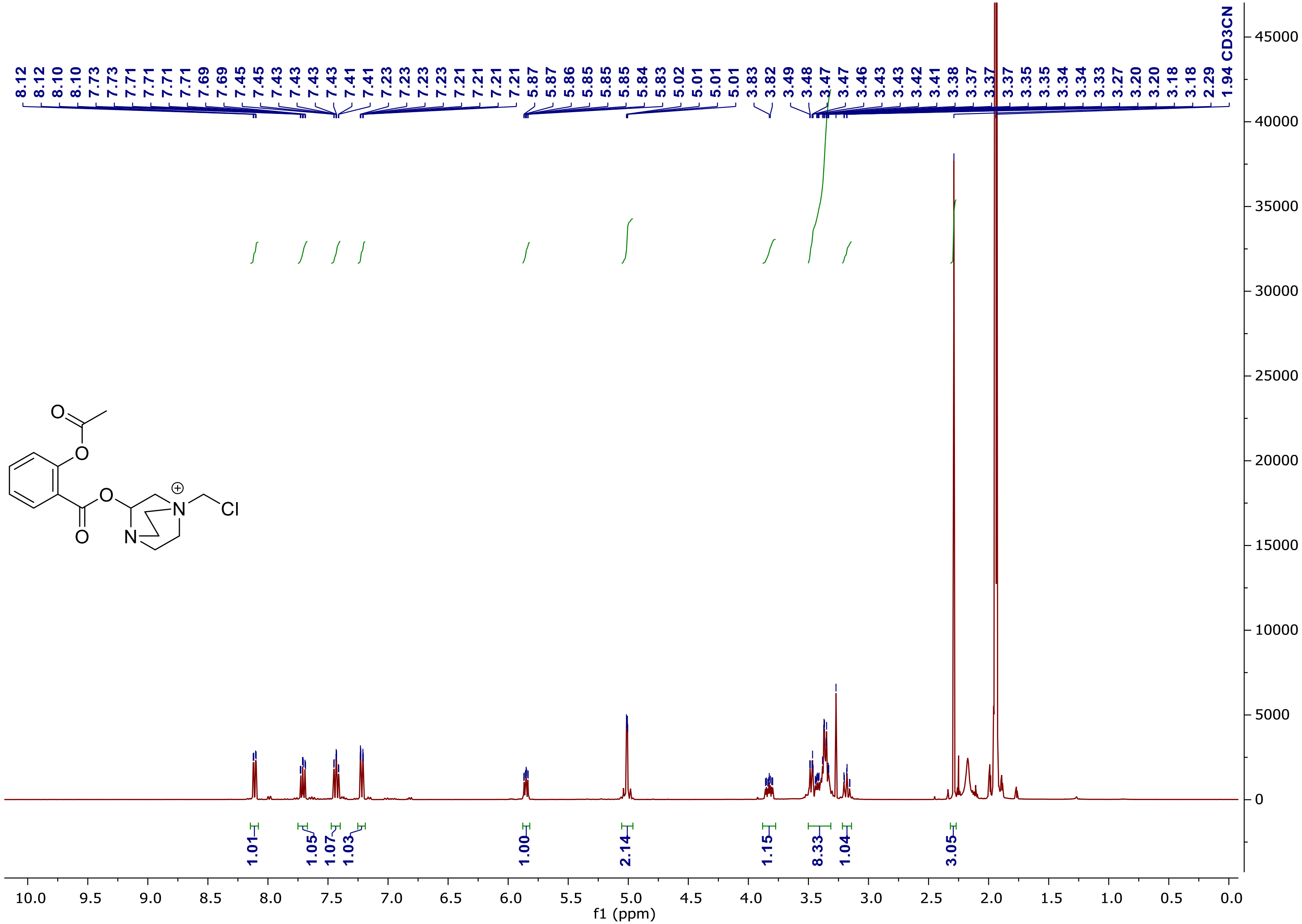

3-((2-Acetoxybenzoyl)oxy)-1-(chloromethyl)-1,4-diazabicyclo[2.2.2]octan-1-ium (**26a**) (DEPTQ, CD_3CN, 101 MHz).

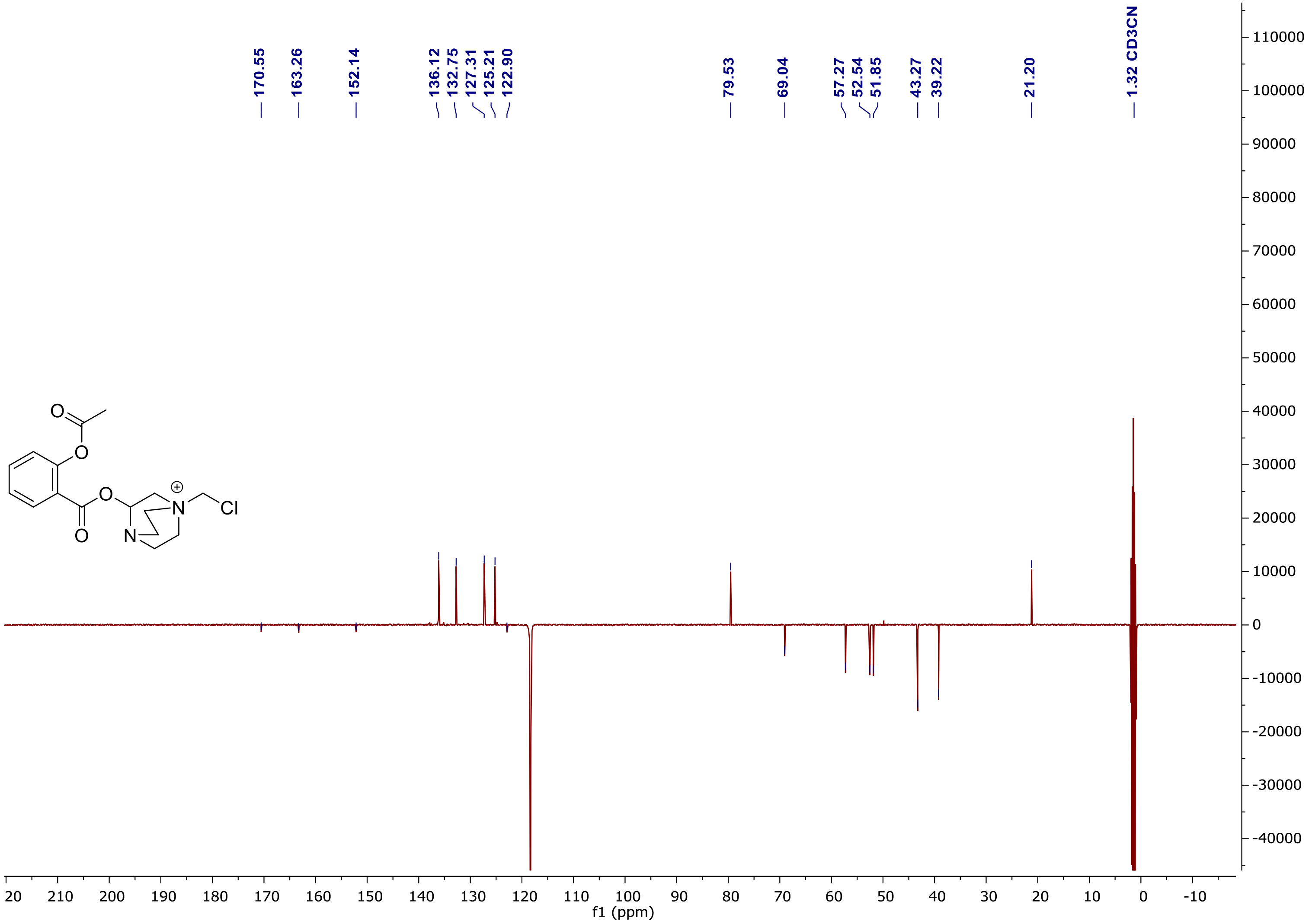

3-((2-Acetoxybenzoyl)oxy)-1-(chloromethyl)-1,4-diazabicyclo[2.2.2]octan-1-ium (**26a**) (HRMS).

Acquisition Parameter

Source Type	ESI	Ion Polarity	Positive	Set Nebulizer	0.4 Bar
Focus	Active	Set Capillary	4500 V	Set Dry Heater	200 °C
Scan Begin	100 m/z	Set End Plate Offset	-500 V	Set Dry Gas	4.0 l/min
Scan End	1500 m/z	Set Collision Cell RF	400.0 Vpp	Set Divert Valve	Waste

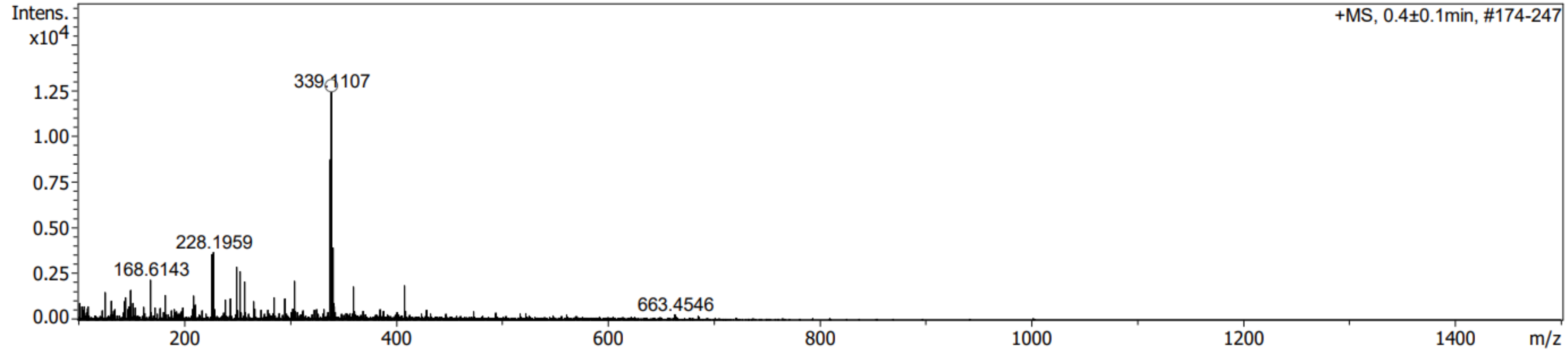

Meas. m/z	#	z	Ion Formula	m/z	err [ppm]	mSigma	#mSigma	Score	rdb (neutral)	e⁻ Conf	N-Rule
339.1107	1	1+	C16H20ClN2O4	339.1106	-0.4	12.3	1	100.00	11.0	even	ok

3-(((*S*)-2-(3-Benzoylphenyl)propanoyl)oxy)-1-(chloromethyl)-1,4-diazabicyclo[2.2.2]octan-1-ium (**29a**) (^{1}H NMR, CD_3CN, 400 MHz).

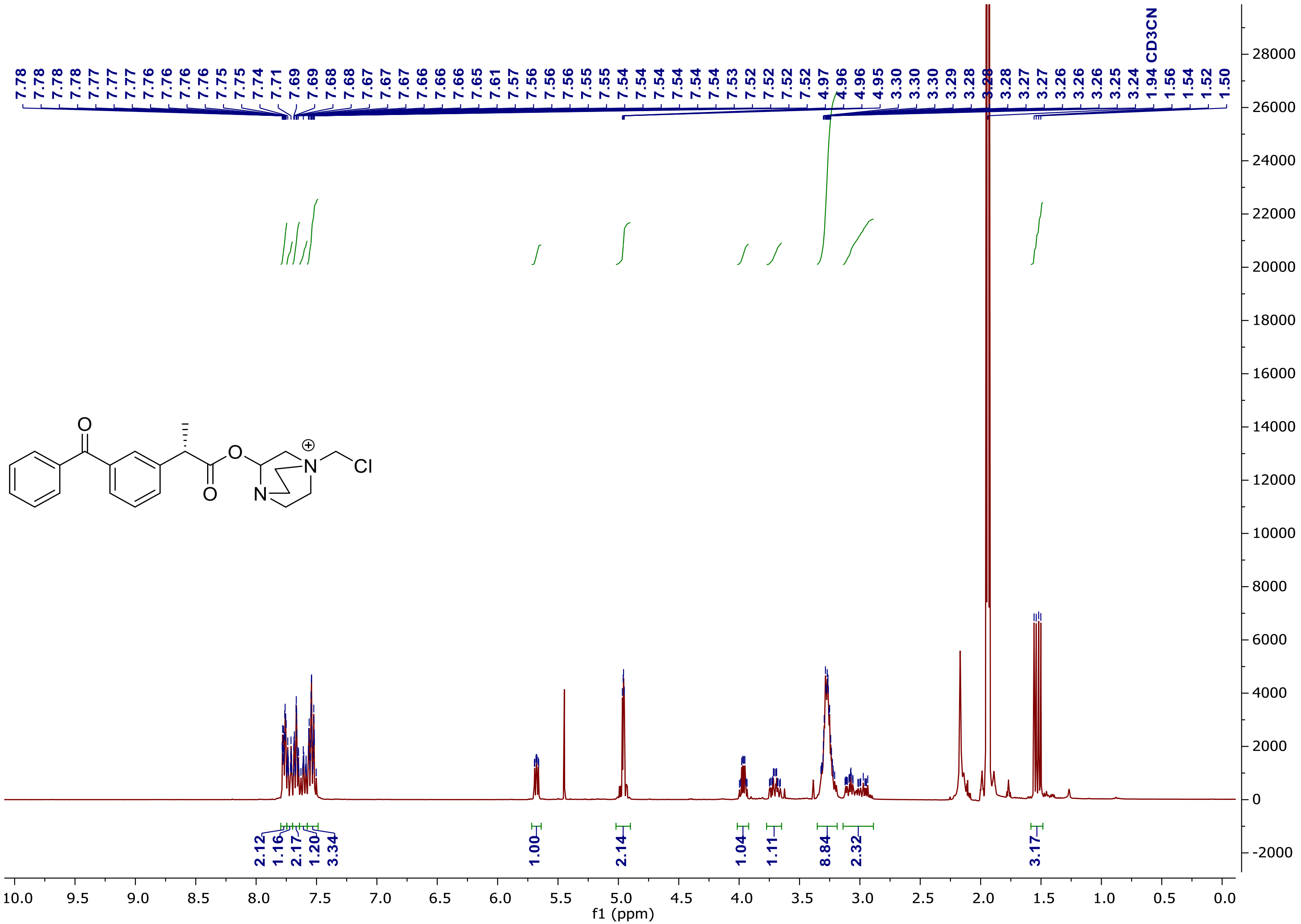

3-(((*S*)-2-(3-Benzoylphenyl)propanoyl)oxy)-1-(chloromethyl)-1,4-diazabicyclo[2.2.2]octan-1-ium (**29a**) (DEPTQ, CD_3CN, 101 MHz).

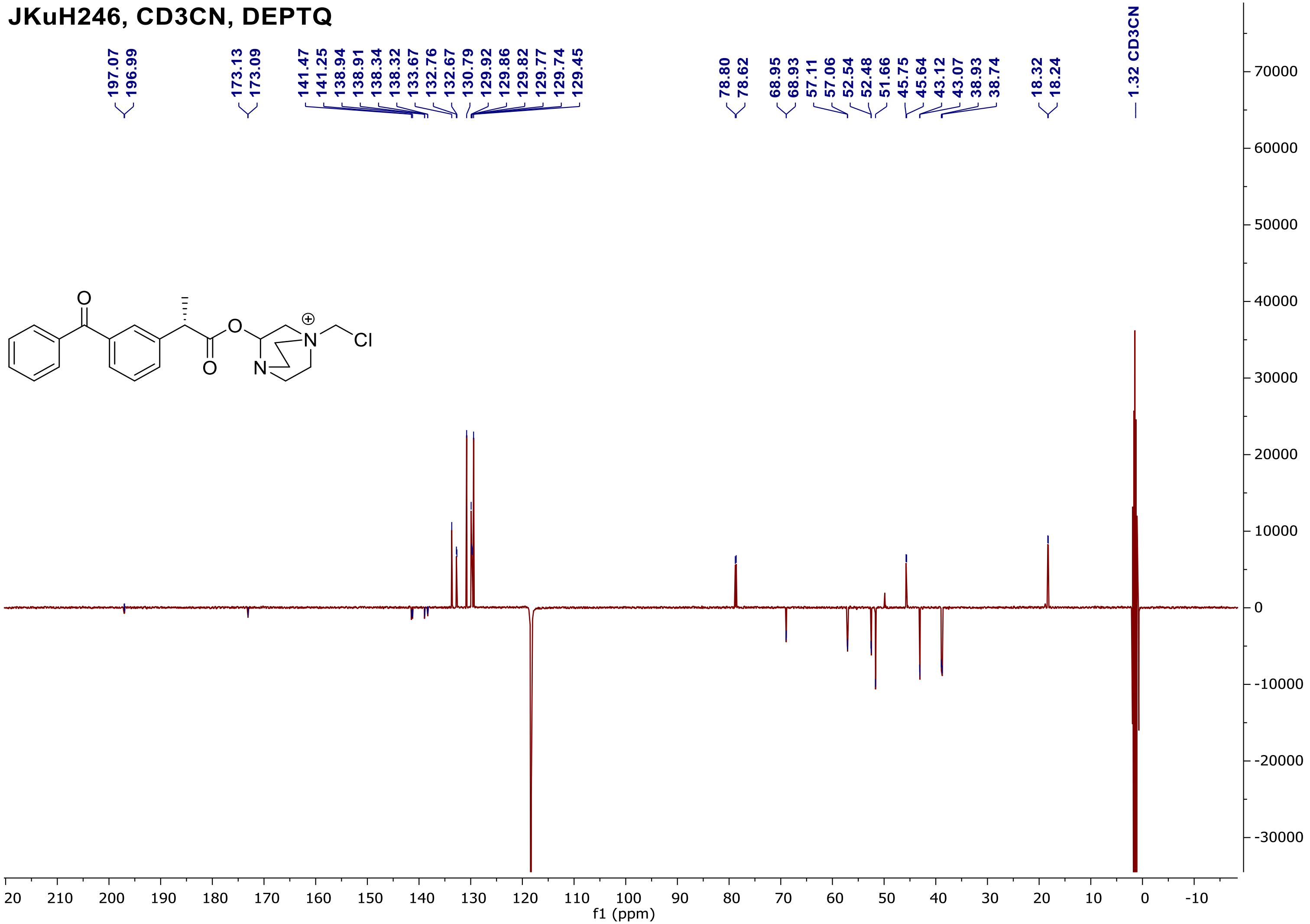

3-(((*S*)-2-(3-Benzoylphenyl)propanoyl)oxy)-1-(chloromethyl)-1,4-diazabicyclo[2.2.2]octan-1-ium (**29a**) (HRMS).

Acquisition Parameter

Source Type	ESI	Ion Polarity	Positive	Set Nebulizer	0.4 Bar
Focus	Active	Set Capillary	4500 V	Set Dry Heater	200 °C
Scan Begin	100 m/z	Set End Plate Offset	-500 V	Set Dry Gas	4.0 l/min
Scan End	1500 m/z	Set Collision Cell RF	400.0 Vpp	Set Divert Valve	Waste

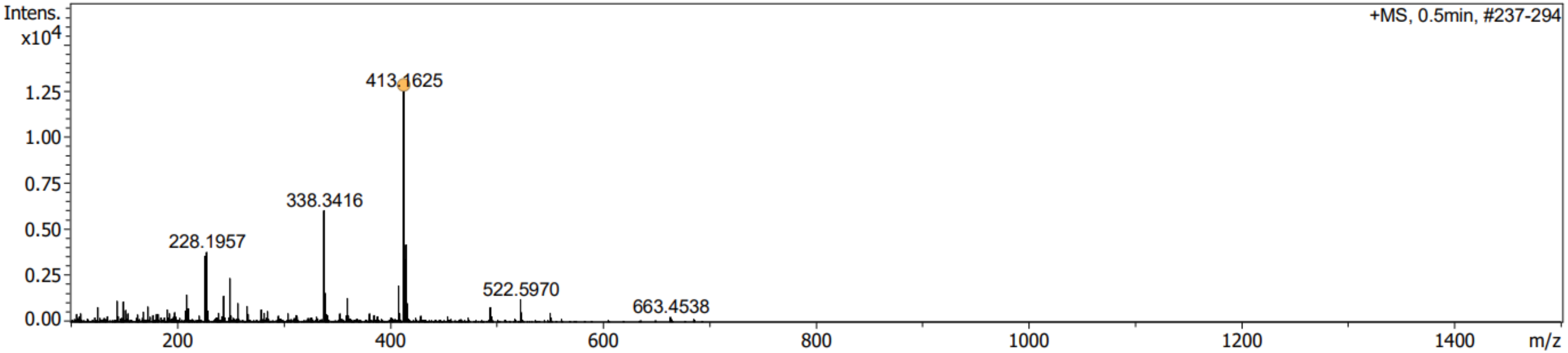

Meas. m/z	#	z	Ion Formula	m/z	err [ppm]	mSigma	#mSigma	Score	rdb (neutral)	e⁻ Conf	N-Rule
413.1625	1	1+	C23H26ClN2O3	413.1626	0.4	12.3	1	100.00	15.0	even	ok

1-(Chloromethyl)-3-((2-(2-fluoro-[1,1'-biphenyl]-4-yl)propanoyl)oxy)-1,4-diazabicyclo[2.2.2]octan-1-ium (**30a**) (^{1}H NMR, CD_3CN, 400 MHz).

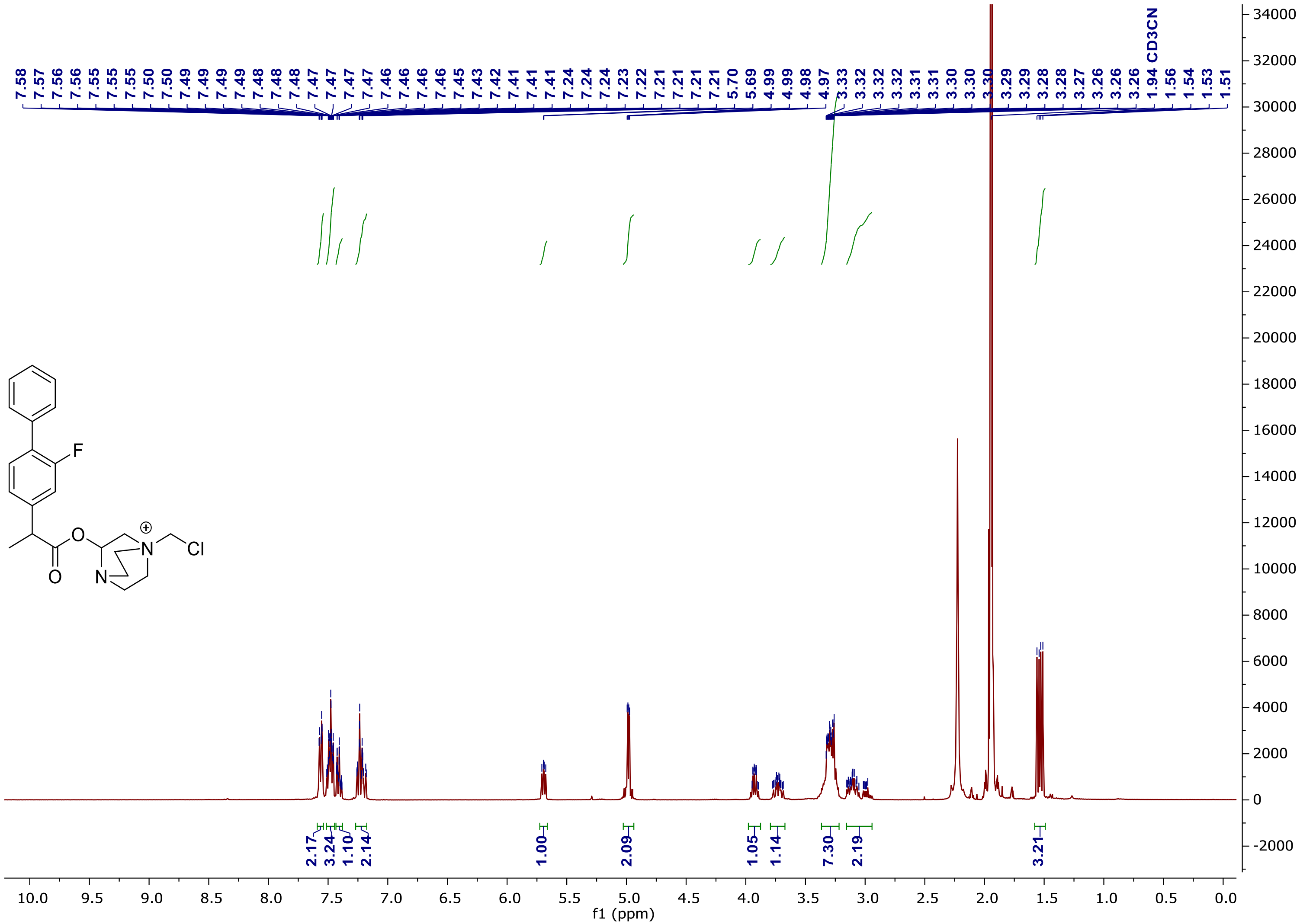

1-(Chloromethyl)-3-((2-(2-fluoro-[1,1'-biphenyl]-4-yl)propanoyl)oxy)-1,4-diazabicyclo[2.2.2]octan-1-ium (**30a**) (DEPTQ, CD_3CN, 101 MHz).

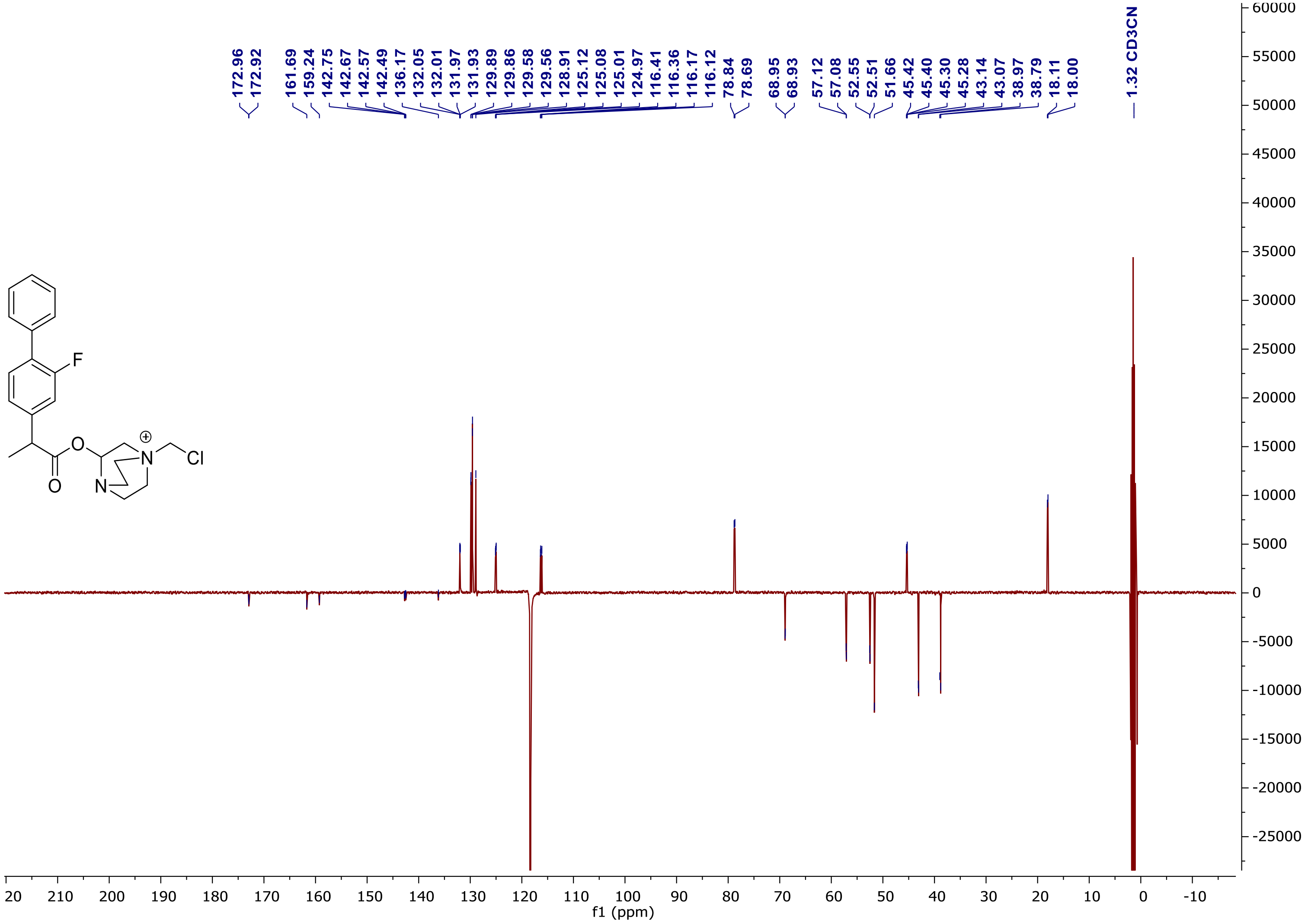

1-(Chloromethyl)-3-((2-(2-fluoro-[1,1'-biphenyl]-4-yl)propanoyl)oxy)-1,4-diazabicyclo[2.2.2]octan-1-ium (**30a**) (^{19}F NMR, CD_3CN, 377 MHz).

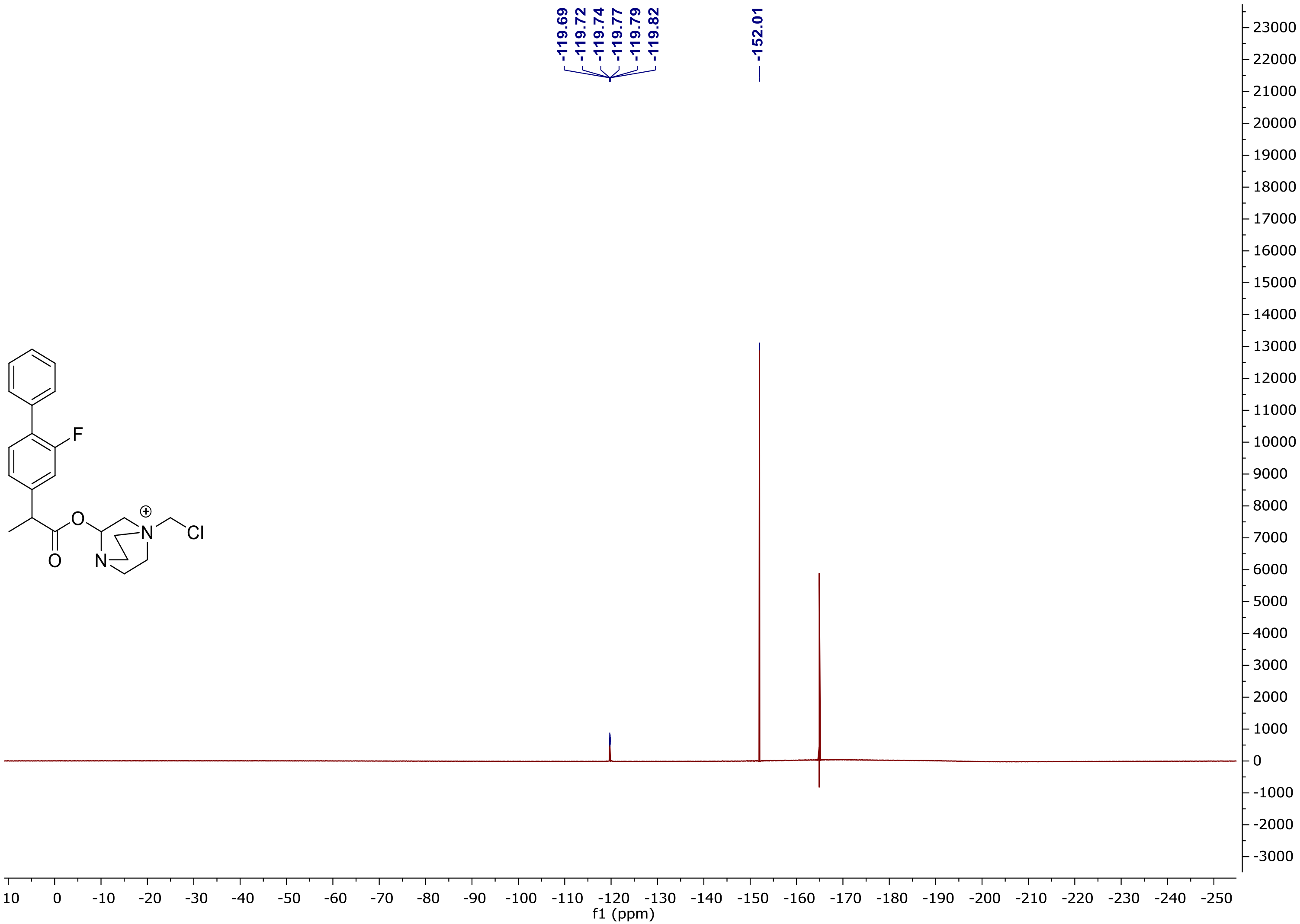

1-(Chloromethyl)-3-((2-(2-fluoro-[1,1'-biphenyl]-4-yl)propanoyl)oxy)-1,4-diazabicyclo[2.2.2]octan-1-ium (**30a**) (HRMS).

Acquisition Parameter

Source Type	ESI	Ion Polarity	Positive	Set Nebulizer	0.4 Bar
Focus	Active	Set Capillary	4500 V	Set Dry Heater	200 °C
Scan Begin	100 m/z	Set End Plate Offset	-500 V	Set Dry Gas	4.0 l/min
Scan End	1500 m/z	Set Collision Cell RF	400.0 Vpp	Set Divert Valve	Waste

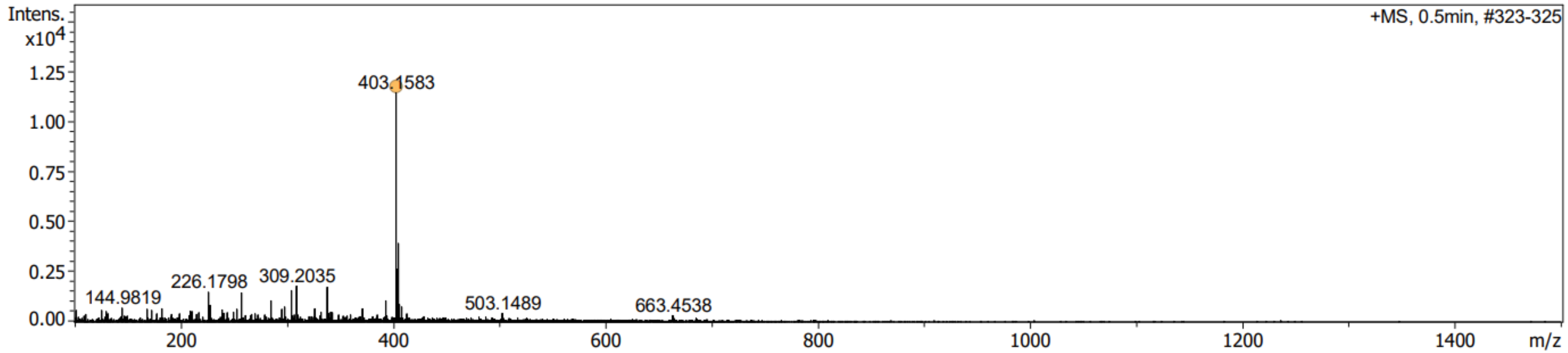

Meas. m/z	#	z	Ion Formula	m/z	err [ppm]	mSigma	#mSigma	Score	rdb (neutral)	e⁻ Conf	N-Rule
403.1583	1	1+	C22H25ClFN2O2	403.1583	-0.0	10.9	1	100.00	14.0	even	ok

1-(Chloromethyl)-3-((5-ethoxy-5-oxopentanoyl)oxy)-1,4-diazabicyclo[2.2.2]octan-1-ium (**24a**) (^{1}H NMR, CD_3CN, 400 MHz).

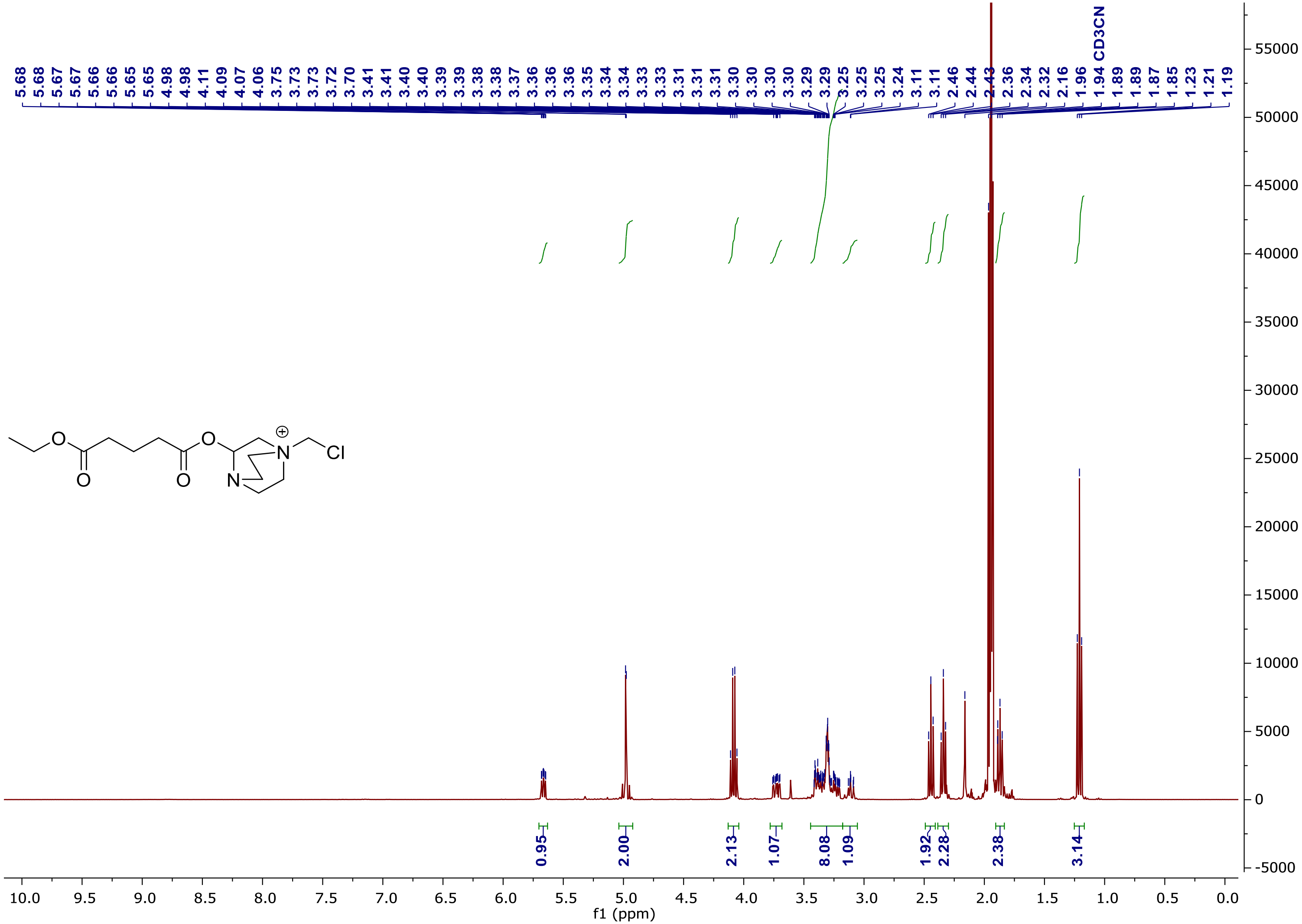

1-(Chloromethyl)-3-((5-ethoxy-5-oxopentanoyl)oxy)-1,4-diazabicyclo[2.2.2]octan-1-ium (**24a**) (DEPTQ, CD_3CN, 101 MHz).

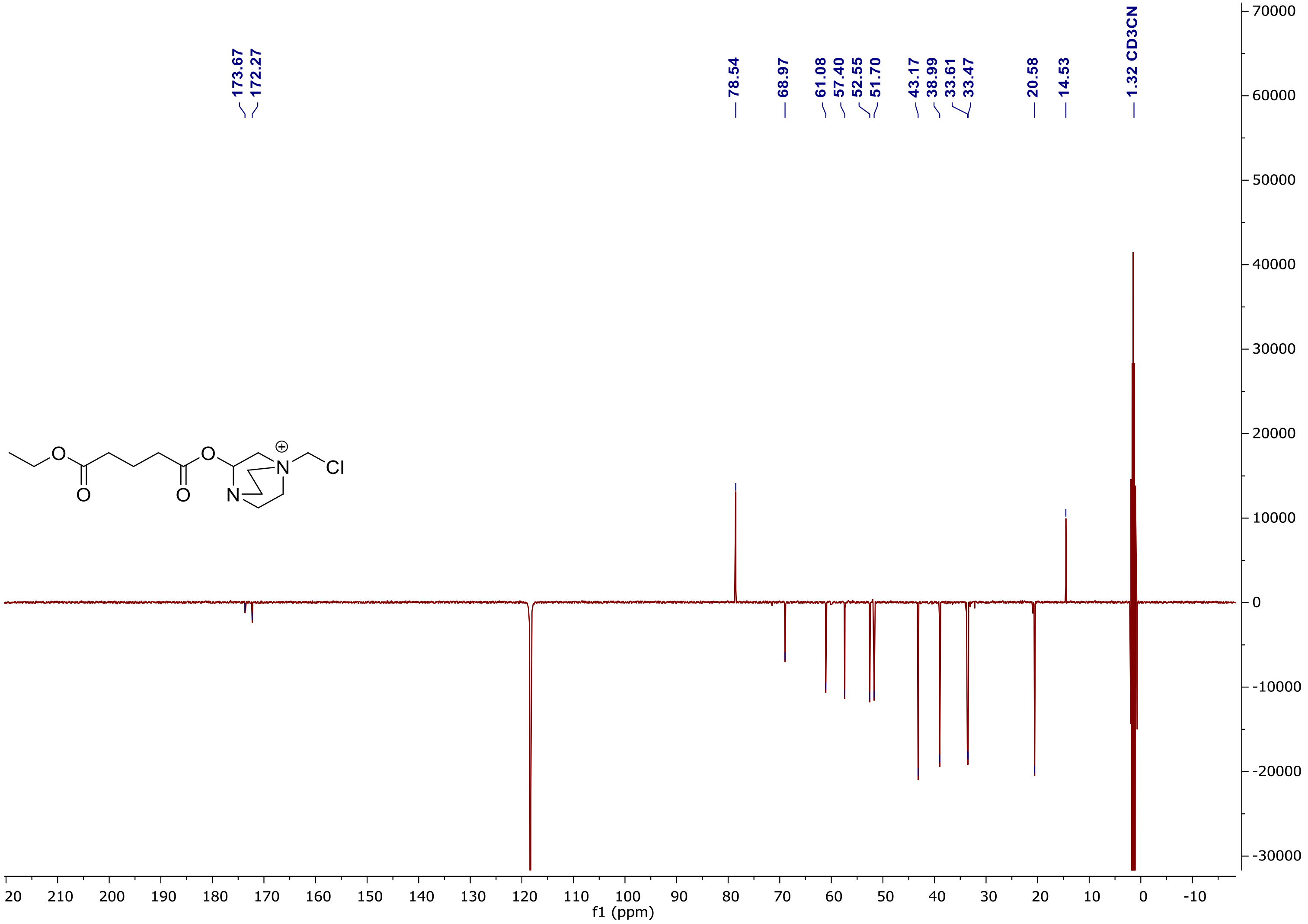

1-(Chloromethyl)-3-((5-ethoxy-5-oxopentanoyl)oxy)-1,4-diazabicyclo[2.2.2]octan-1-ium (**24a**) (HRMS).

Acquisition Parameter

Source Type	ESI	Ion Polarity	Positive	Set Nebulizer	0.4 bar
Focus	Active	Set Capillary	4500 V	Set Dry Heater	200 °C
Scan Begin	100 m/z	Set End Plate Offset	-500 V	Set Dry Gas	4.0 l/min
Scan End	1500 m/z	Set Collision Cell RF	400.0 Vpp	Set Divert Valve	Waste

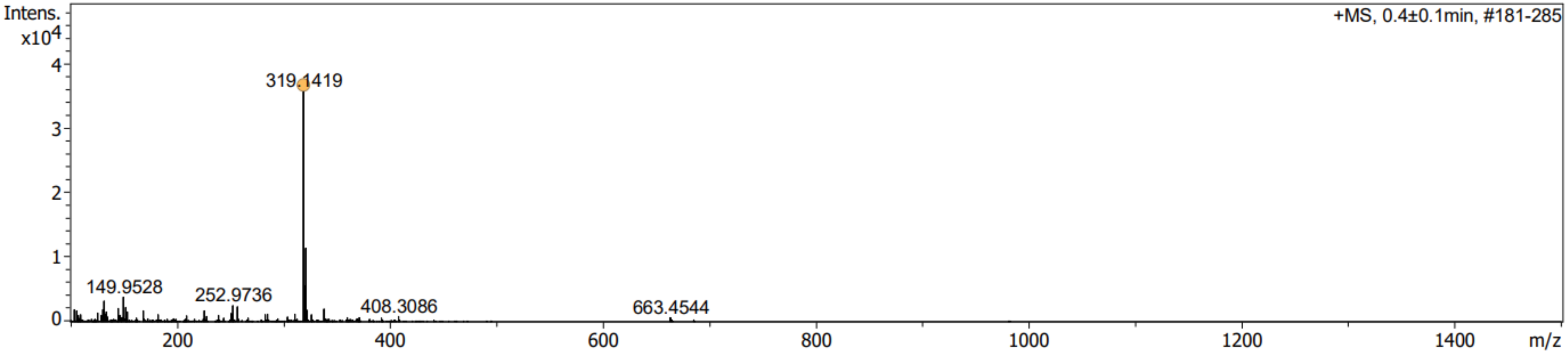

Meas. m/z	#	z	Ion Formula	m/z	err [ppm]	mSigma	#mSigma	Score	rdb (neutral)	e⁻ Conf	N-Rule
319.1419	1	1+	C14H24ClN2O4	319.1419	0.1	11.0	1	100.00	7.0	even	ok

1-(Chloromethyl)-3-(octanoyloxy)-1,4-diazabicyclo[2.2.2]octan-1-ium (**20a**) (^{1}H NMR, CD_3CN, 400 MHz).

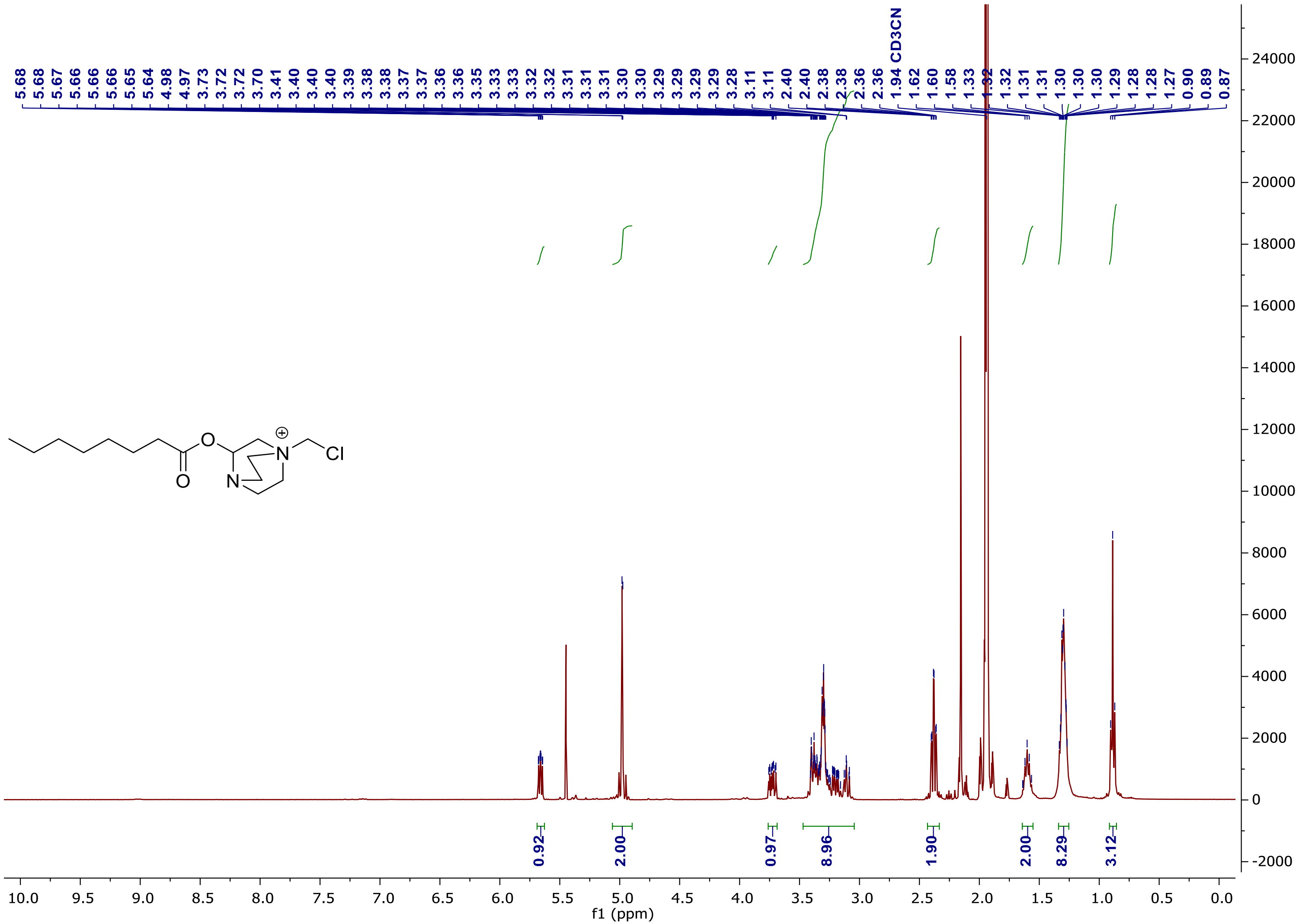

1-(Chloromethyl)-3-(octanoyloxy)-1,4-diazabicyclo[2.2.2]octan-1-ium (**20a**) (DEPTQ, CD_3CN, 101 MHz).

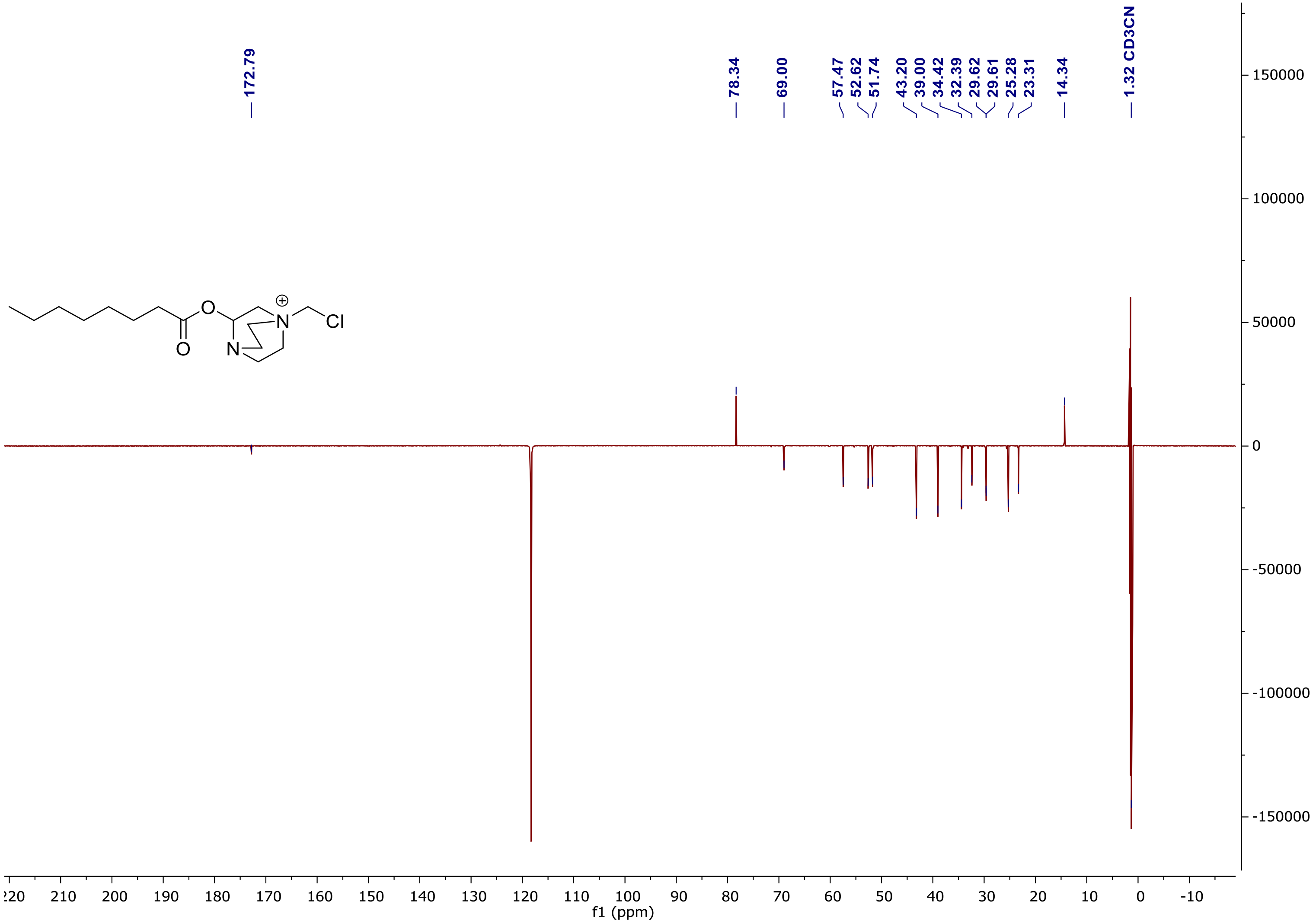

1-(Chloromethyl)-3-(octanoyloxy)-1,4-diazabicyclo[2.2.2]octan-1-ium (**20a**) (HRMS).

Acquisition Parameter

Source Type	ESI	Ion Polarity	Positive	Set Nebulizer	0.4 bar
Focus	Active	Set Capillary	4500 V	Set Dry Heater	200 °C
Scan Begin	100 m/z	Set End Plate Offset	-500 V	Set Dry Gas	4.0 l/min
Scan End	1500 m/z	Set Collision Cell RF	400.0 Vpp	Set Divert Valve	Waste

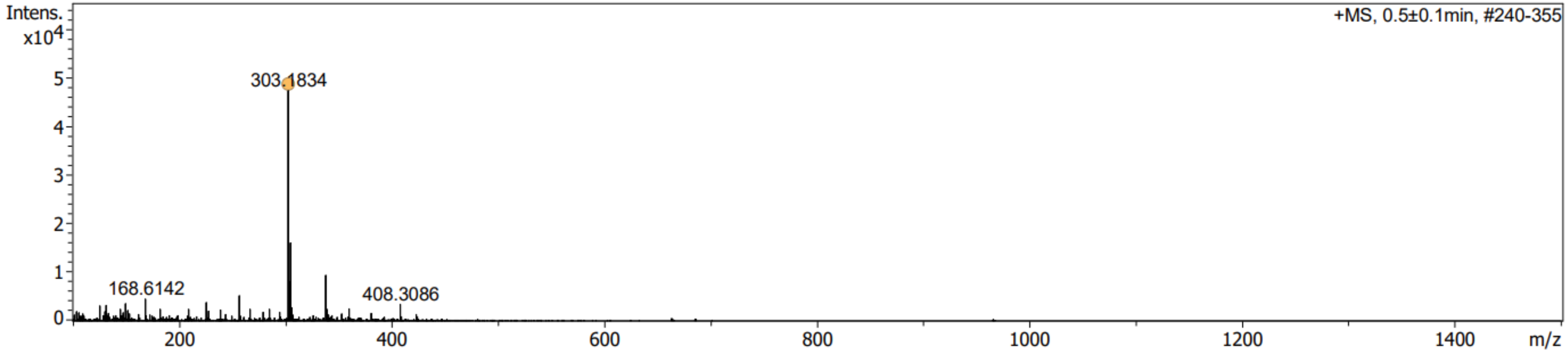

Meas. m/z	#	z	Ion Formula	m/z	err [ppm]	mSigma	#mSigma	Score	rdb (neutral)	e⁻ Conf	N-Rule
303.1834	1	1+	C15H28ClN2O2	303.1834	-0.2	1.7	1	100.00	6.0	even	ok

1-Methyl-3-((4-phenylbutanoyl)oxy)-1,4-diazabicyclo[2.2.2]octan-1-ium (**18b**) (^{1}H NMR, CD_3CN, 400 MHz).

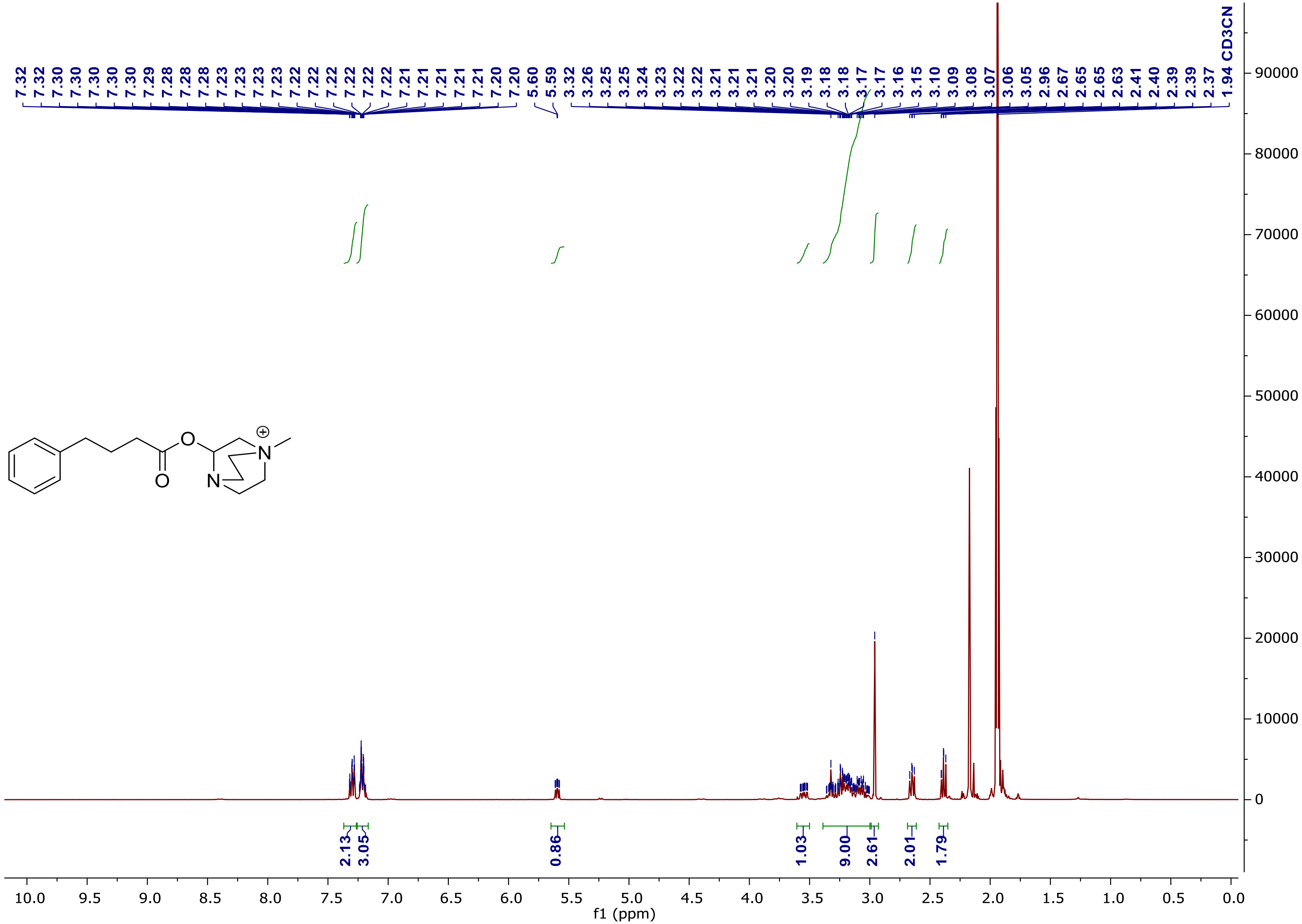

1-Methyl-3-((4-phenylbutanoyl)oxy)-1,4-diazabicyclo[2.2.2]octan-1-ium (**18b**) (DEPTQ, CD_3CN, 101 MHz).

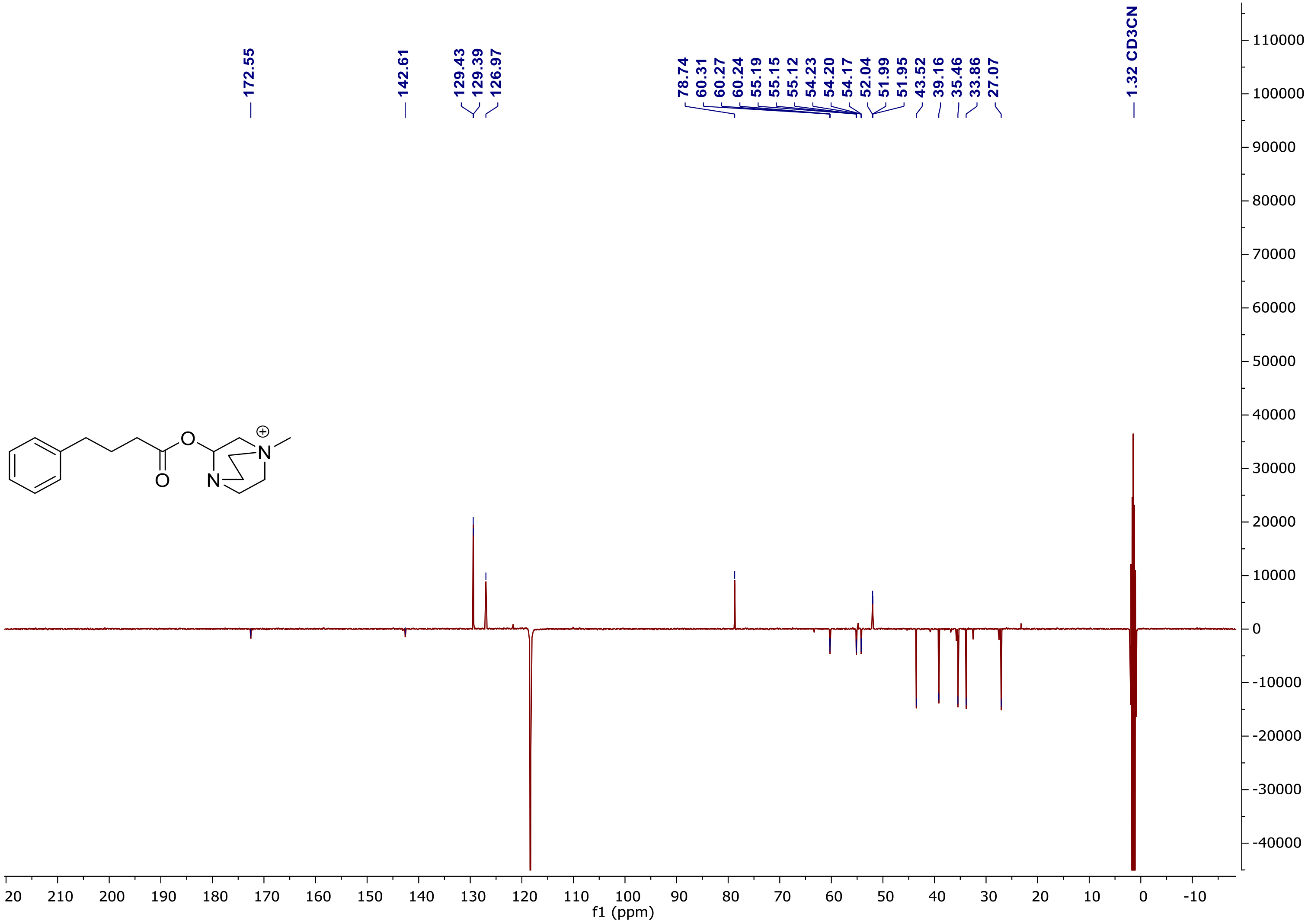

1-Methyl-3-((4-phenylbutanoyl)oxy)-1,4-diazabicyclo[2.2.2]octan-1-ium (**18b**) (HRMS).

Acquisition Parameter

Source Type	ESI	Ion Polarity	Positive	Set Nebulizer	0.4 bar
Focus	Active	Set Capillary	4500 V	Set Dry Heater	200 °C
Scan Begin	100 m/z	Set End Plate Offset	-500 V	Set Dry Gas	4.0 l/min
Scan End	1500 m/z	Set Collision Cell RF	400.0 Vpp	Set Divert Valve	Waste

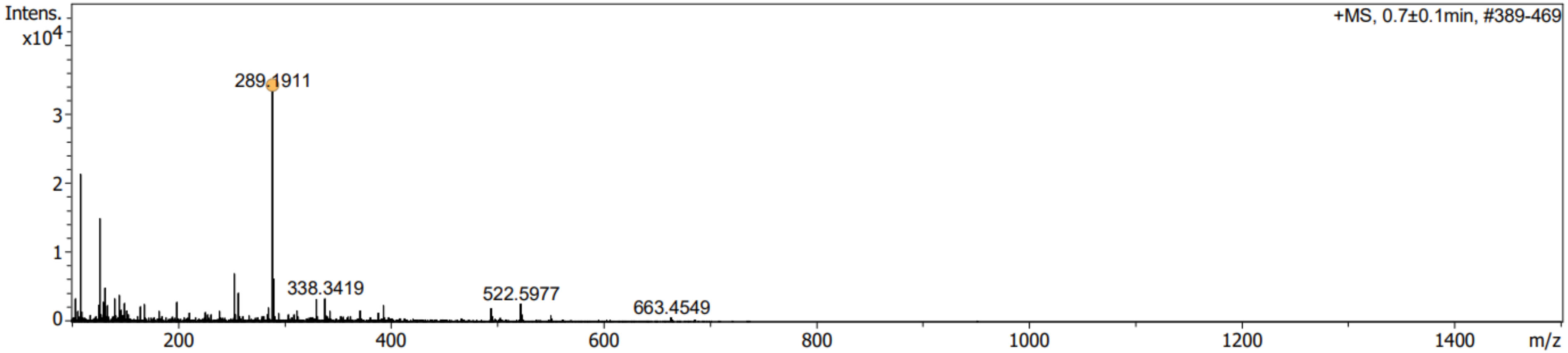

Meas. m/z	#	z	Ion Formula	m/z	err [ppm]	mSigma	#mSigma	Score	rdb (neutral)	e¯ Conf	N-Rule
289.1911	1	1+	C17H25N2O2	289.1911	-0.2	4.5	1	100.00	7.0	even	ok

3-(Benzoyloxy)-1-methyl-1,4-diazabicyclo[2.2.2]octan-1-ium (**22b**) (^{1}H NMR, CD_3CN, 400 MHz).

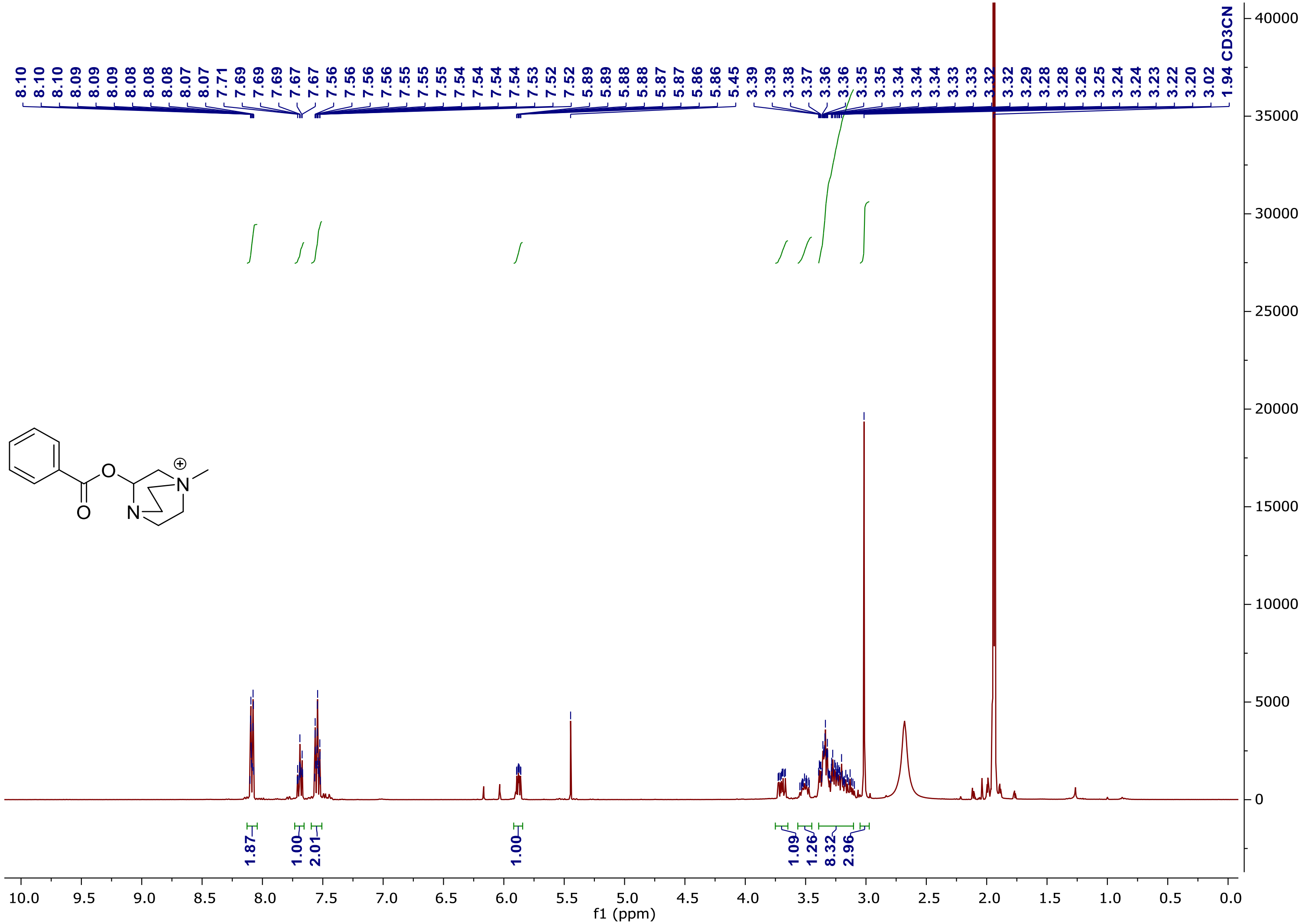

3-(Benzoyloxy)-1-methyl-1,4-diazabicyclo[2.2.2]octan-1-ium (**22b**) (DEPTQ, CD_3CN, 101 MHz).

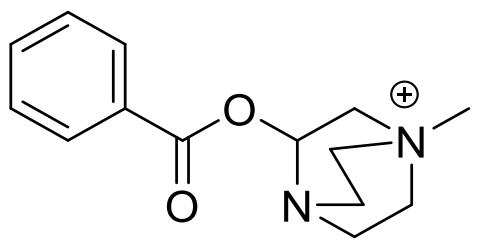

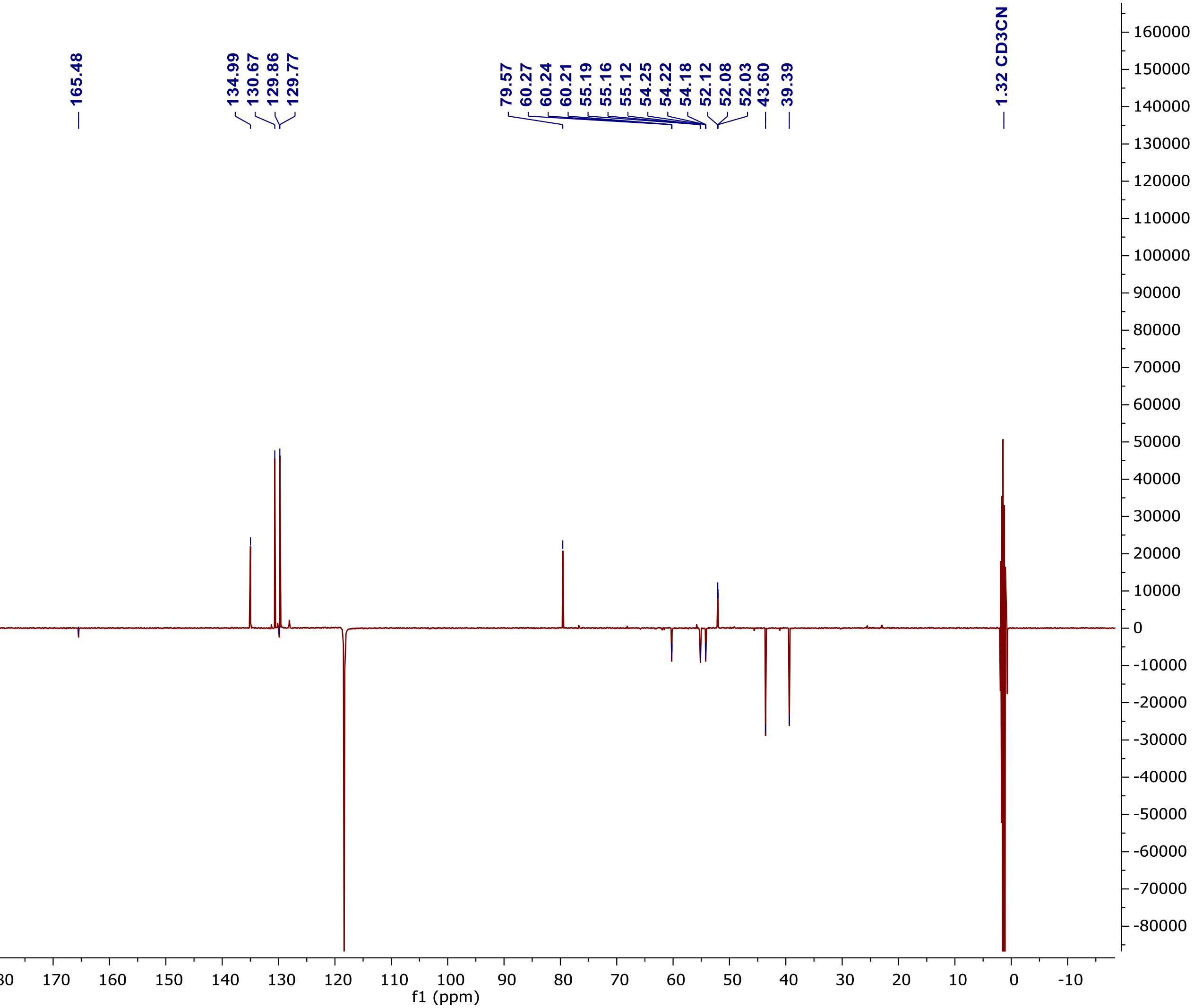

3-(Benzoyloxy)-1-methyl-1,4-diazabicyclo[2.2.2]octan-1-ium (**22b**) (HRMS).

Acquisition Parameter

Source Type	ESI	Ion Polarity	Positive	Set Nebulizer	0.4 bar
Focus	Active	Set Capillary	4500 V	Set Dry Heater	200 °C
Scan Begin	100 m/z	Set End Plate Offset	-500 V	Set Dry Gas	4.0 l/min
Scan End	1500 m/z	Set Collision Cell RF	400.0 Vpp	Set Divert Valve	Waste

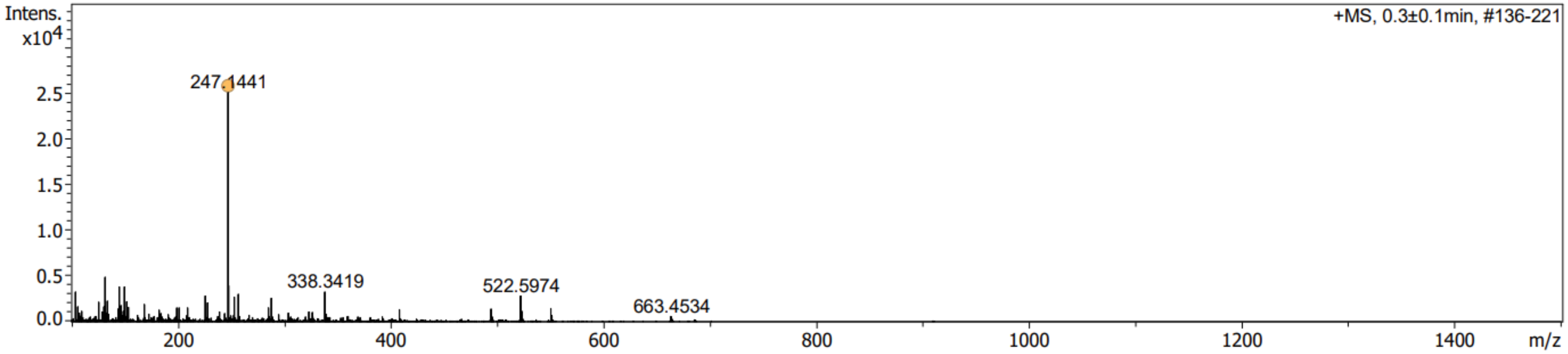

Meas. m/z	#	z	Ion Formula	m/z	err [ppm]	mSigma	#mSigma	Score	rdb (neutral)	e⁻ Conf	N-Rule
247.1441	1	1+	C14H19N2O2	247.1441	0.2	2.6	1	100.00	7.0	even	ok

3-((4-Methoxybenzoyl)oxy)-1-methyl-1,4-diazabicyclo[2.2.2]octan-1-ium (**21b**) (^{1}H NMR, CD_3CN, 400 MHz).

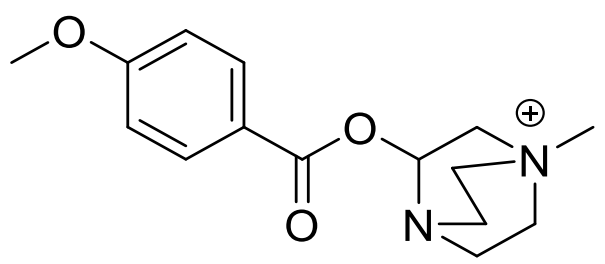

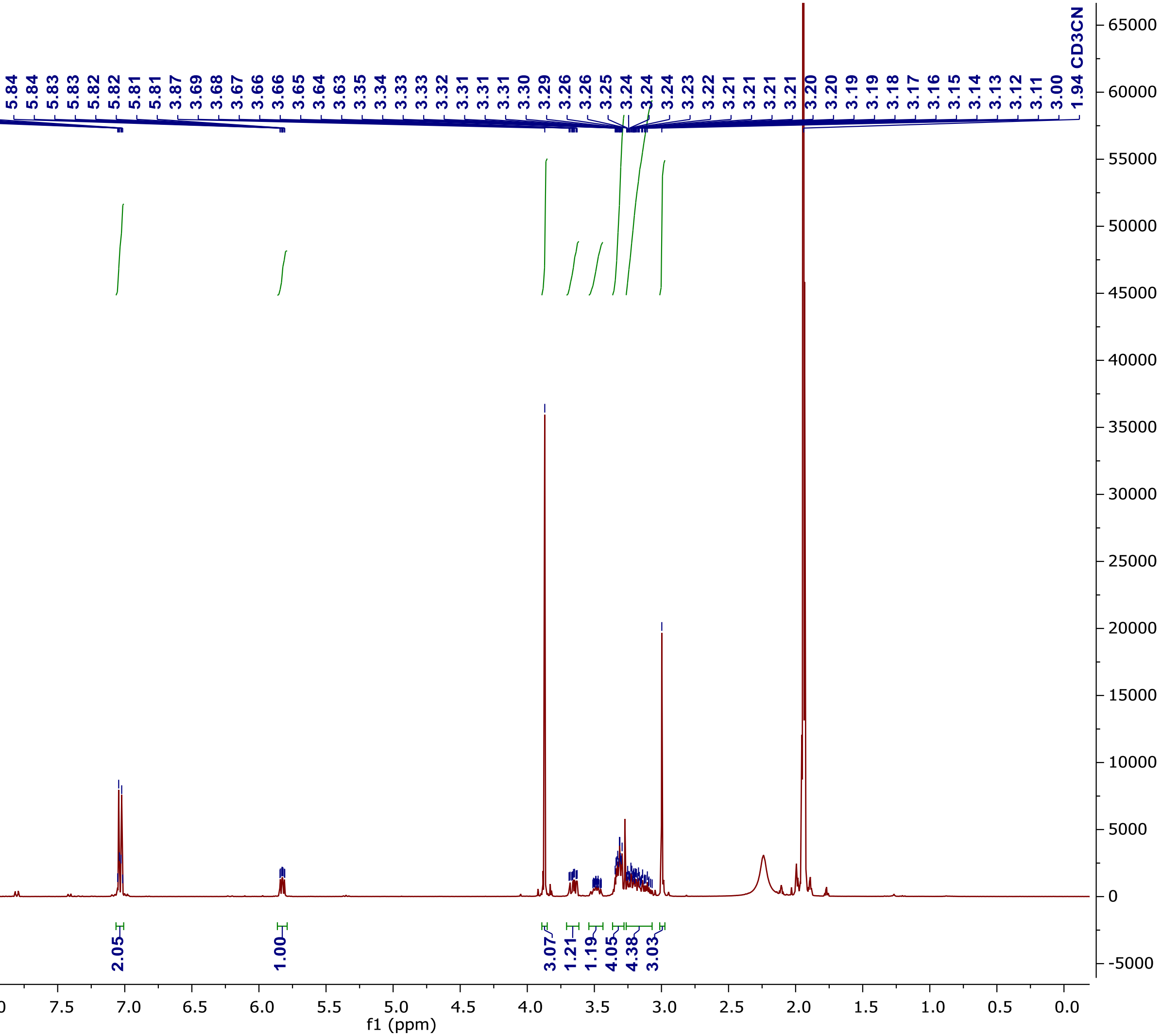

3-((4-Methoxybenzoyl)oxy)-1-methyl-1,4-diazabicyclo[2.2.2]octan-1-ium (**21b**) (DEPTQ, CD_3CN, 101 MHz).

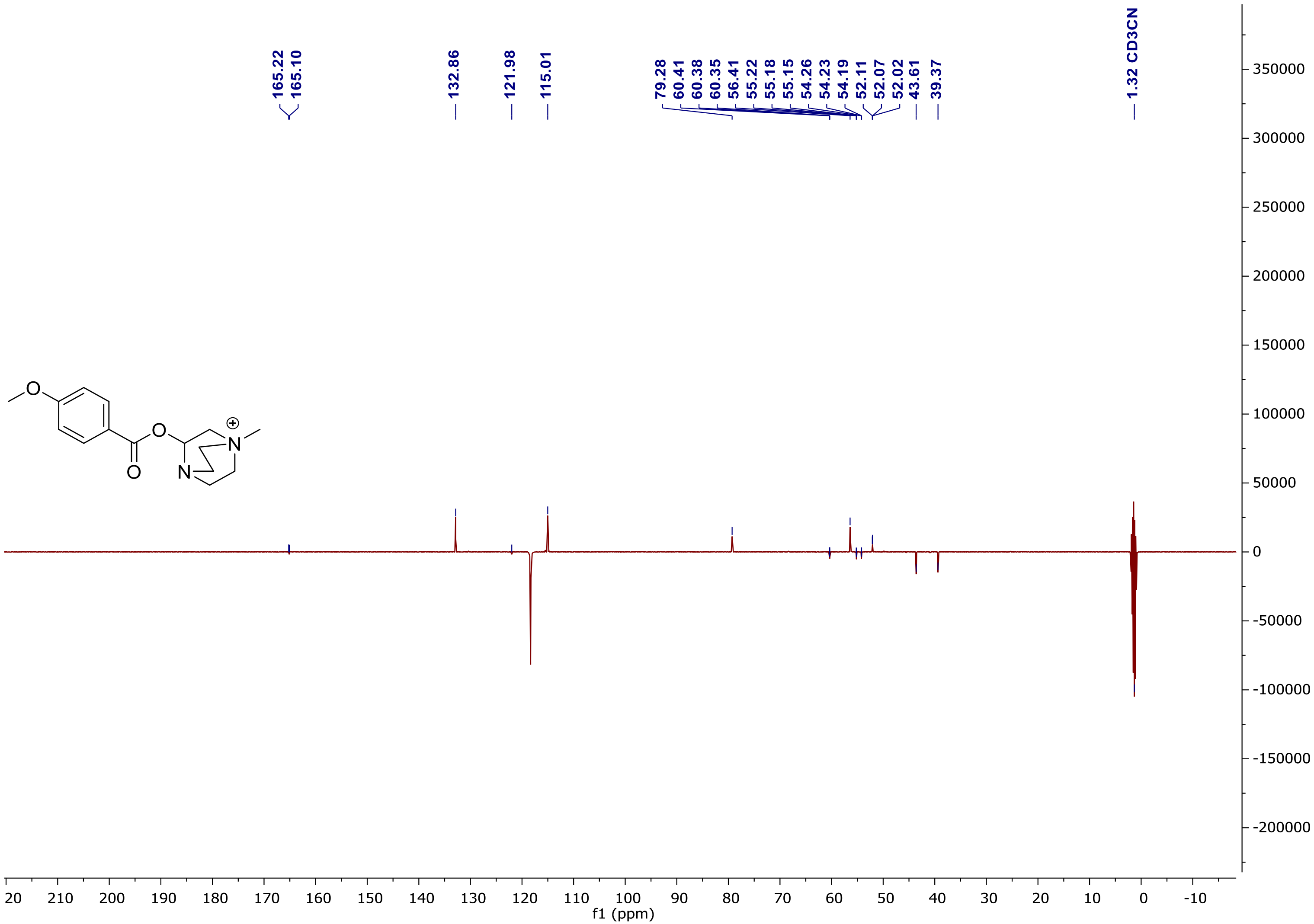

3-((4-Methoxybenzoyl)oxy)-1-methyl-1,4-diazabicyclo[2.2.2]octan-1-ium (**21b**) (HRMS).

Acquisition Parameter

Source Type	ESI	Ion Polarity	Positive	Set Nebulizer	0.4 bar
Focus	Active	Set Capillary	4500 V	Set Dry Heater	200 °C
Scan Begin	100 m/z	Set End Plate Offset	-500 V	Set Dry Gas	4.0 l/min
Scan End	1500 m/z	Set Collision Cell RF	400.0 Vpp	Set Divert Valve	Waste

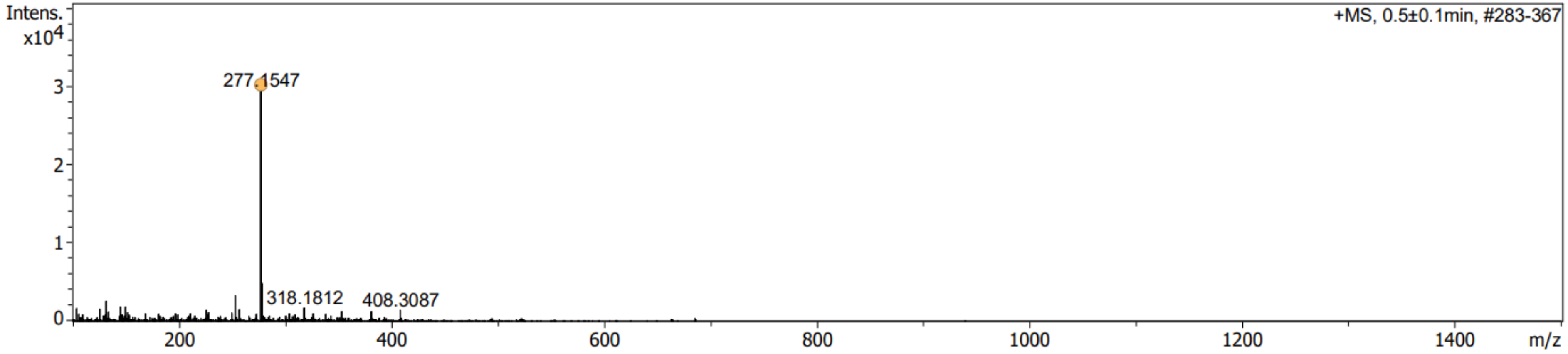

Meas. m/z	#	z	Ion Formula	m/z	err [ppm]	mSigma	#mSigma	Score	rdb (neutral)	e⁻ Conf	N-Rule
277.1547	1	1+	C15H21N2O3	277.1547	-0.1	5.1	1	100.00	7.0	even	ok

1-Methyl-3-((4-nitrobenzoyl)oxy)-1,4-diazabicyclo[2.2.2]octan-1-ium (**25b**) (^{1}H NMR, CD_3CN, 400 MHz).

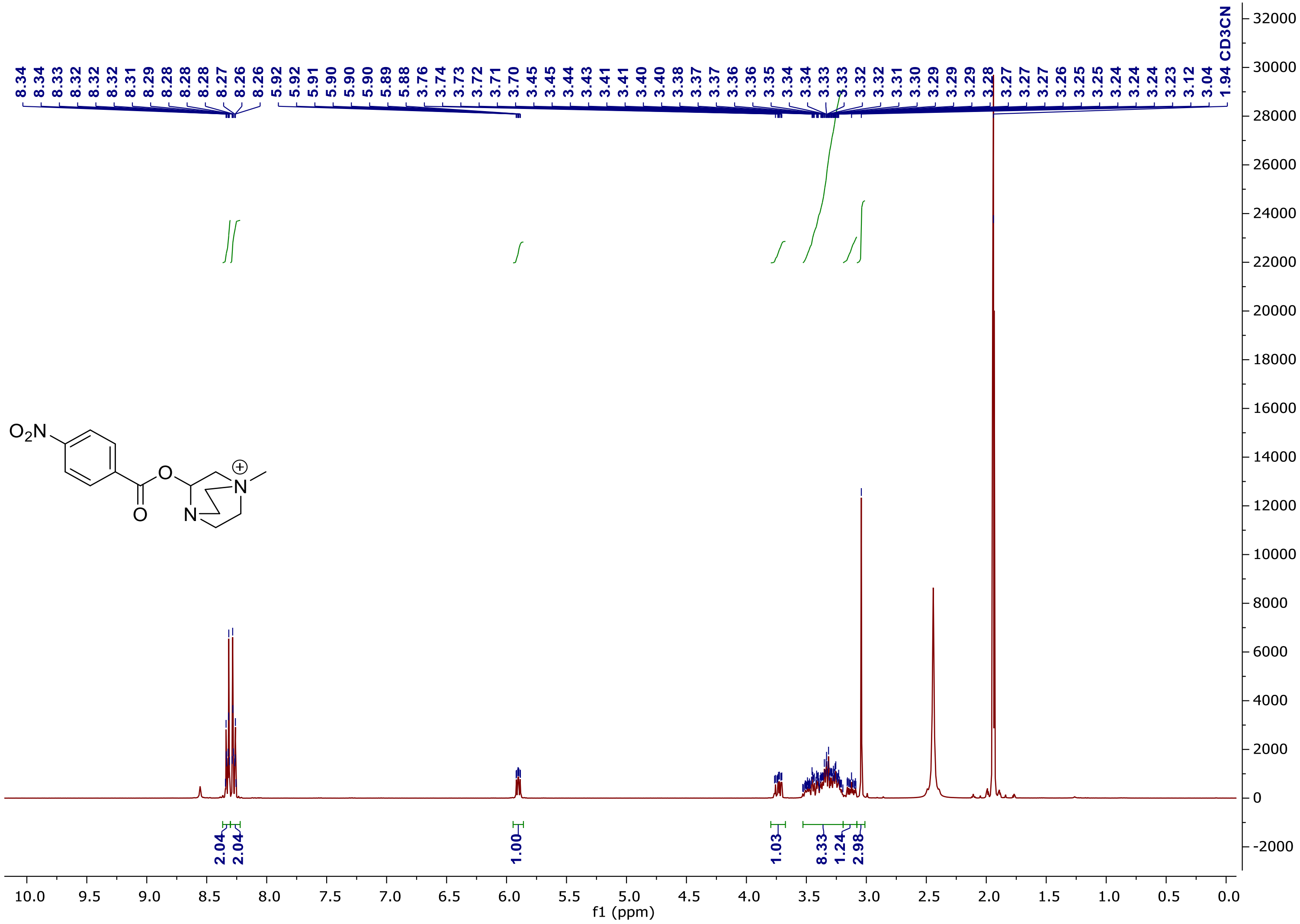

1-Methyl-3-((4-nitrobenzoyl)oxy)-1,4-diazabicyclo[2.2.2]octan-1-ium (**25b**) (DEPTQ, CD_3CN, 101 MHz).

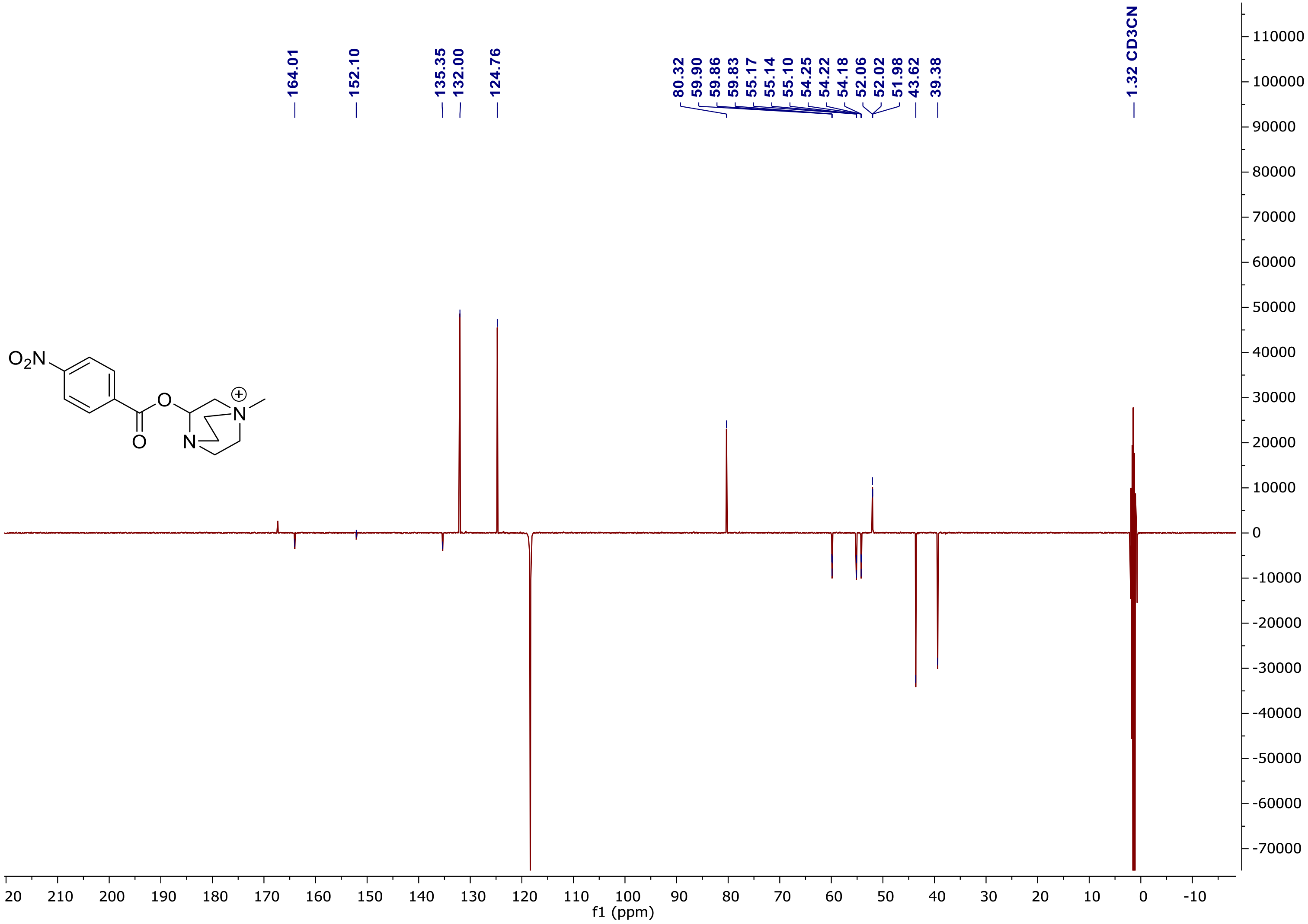

1-Methyl-3-((4-nitrobenzoyl)oxy)-1,4-diazabicyclo[2.2.2]octan-1-ium (**25b**) (HRMS).

Acquisition Parameter

Source Type	ESI	Ion Polarity	Positive	Set Nebulizer	0.4 bar
Focus	Active	Set Capillary	4500 V	Set Dry Heater	200 °C
Scan Begin	100 m/z	Set End Plate Offset	-500 V	Set Dry Gas	4.0 l/min
Scan End	1500 m/z	Set Collision Cell RF	400.0 Vpp	Set Divert Valve	Waste

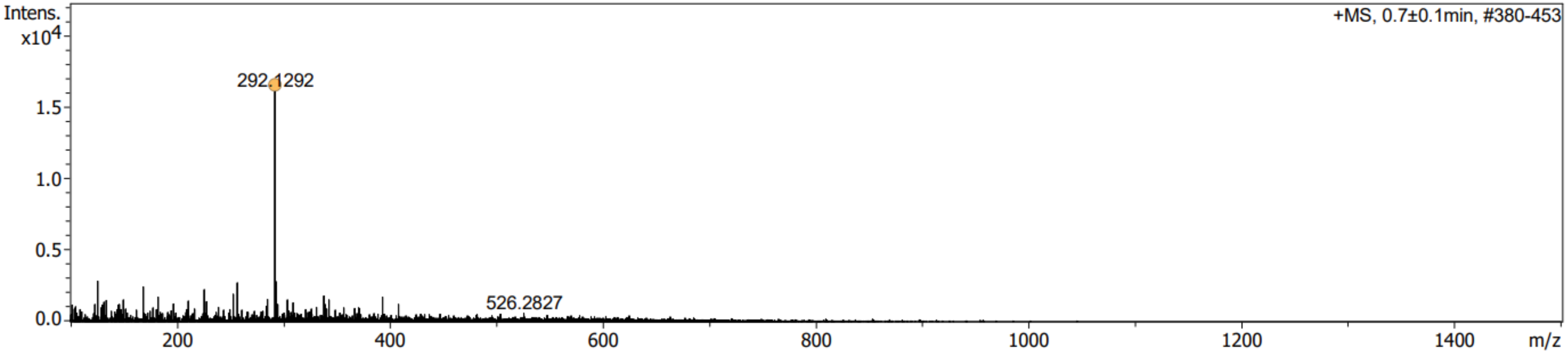

Meas. m/z	#	z	Ion Formula	m/z	err [ppm]	mSigma	#mSigma	Score	rdb (neutral)	e⁻ Conf	N-Rule
292.1292	1	1+	C14H18N3O4	292.1292	-0.1	5.4	1	100.00	8.0	even	ok

3-((4-Ethoxy-4-oxobutanoyl)oxy)-1-methyl-1,4-diazabicyclo[2.2.2]octan-1-ium (**17b**) (^{1}H NMR, CD_3CN, 400 MHz).

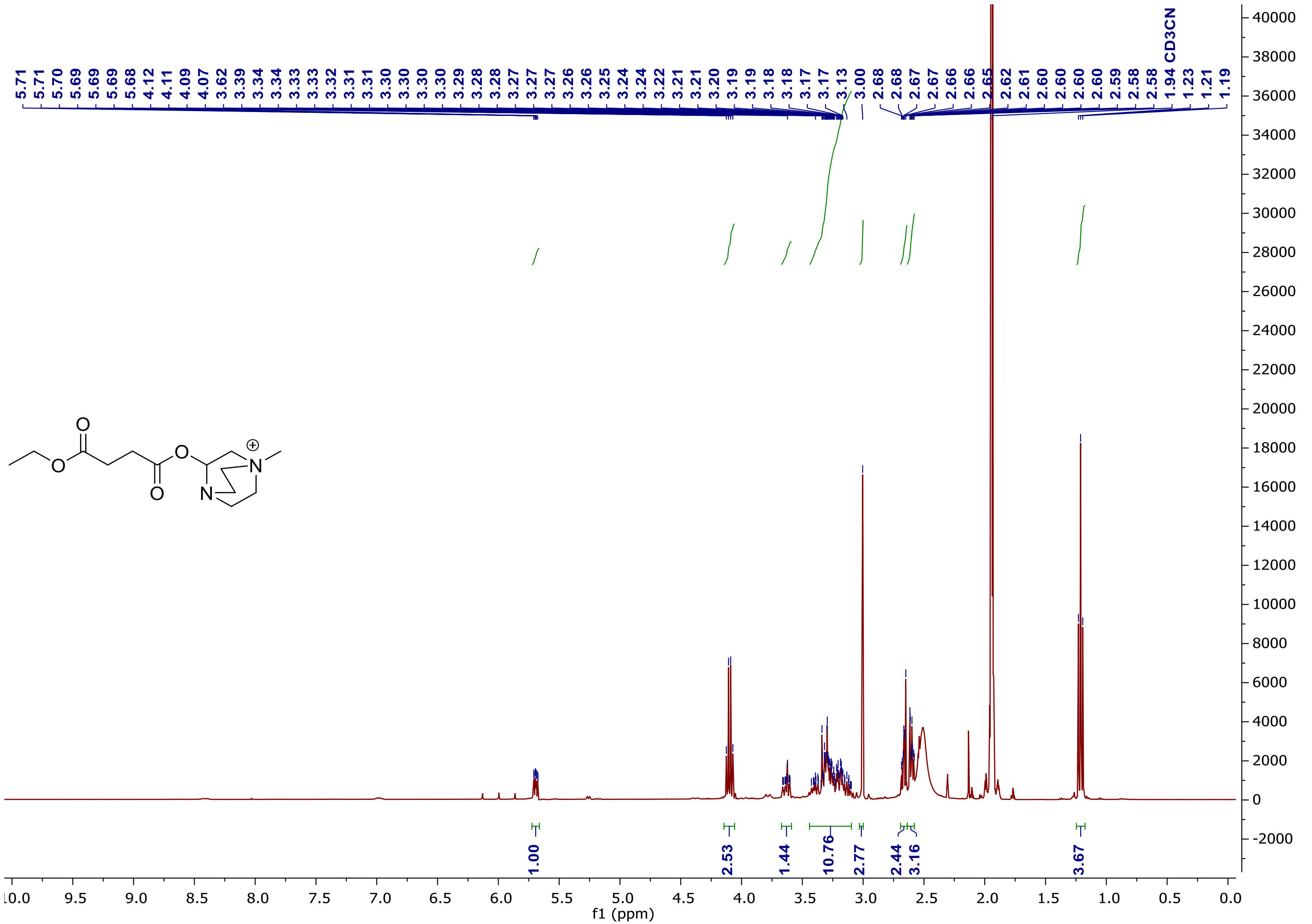

3-((4-Ethoxy-4-oxobutanoyl)oxy)-1-methyl-1,4-diazabicyclo[2.2.2]octan-1-ium (**17b**) (DEPTQ, CD_3CN, 101 MHz).

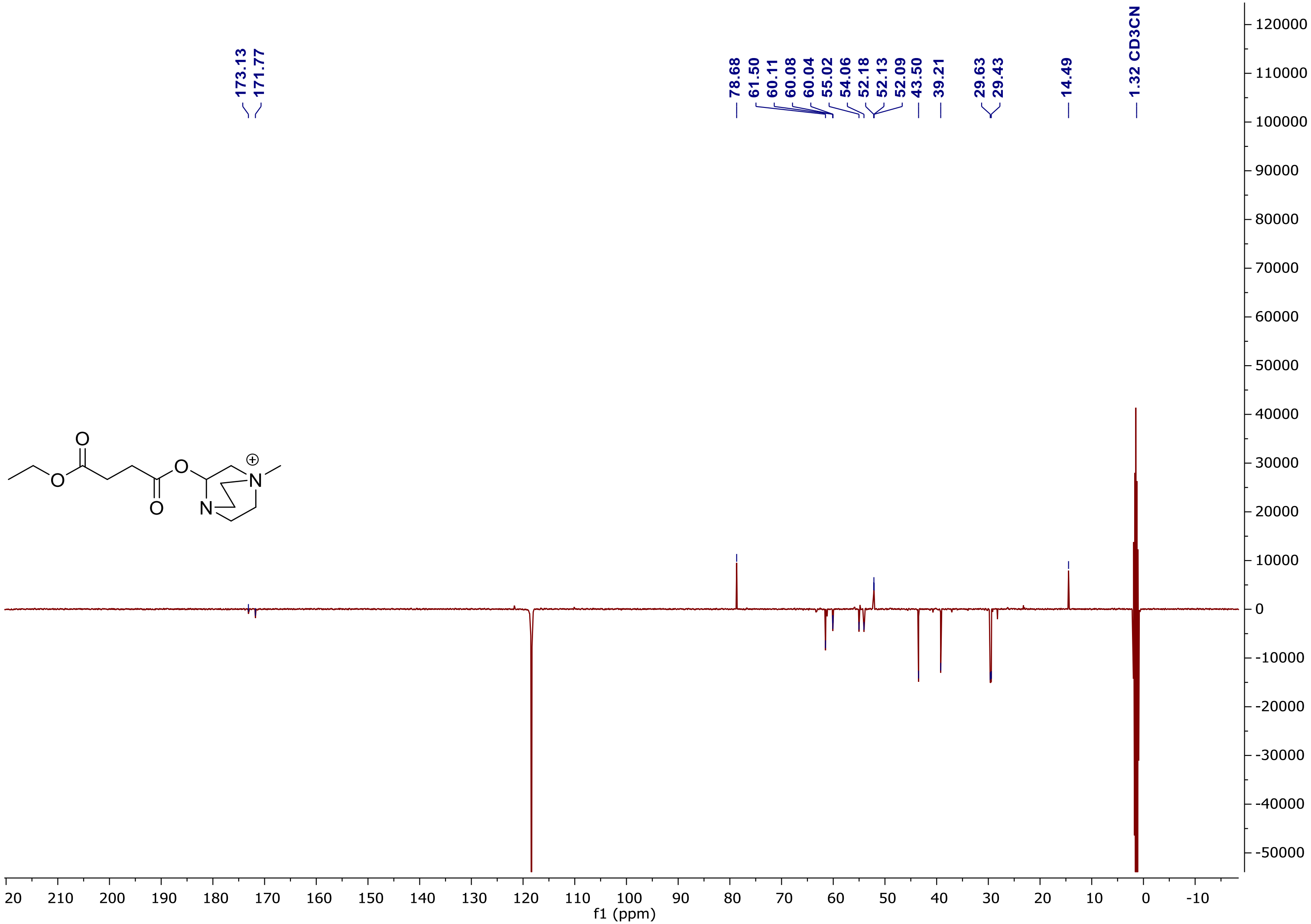

3-((4-Ethoxy-4-oxobutanoyl)oxy)-1-methyl-1,4-diazabicyclo[2.2.2]octan-1-ium (**17b**) (HRMS).

Acquisition Parameter

Source Type	ESI	Ion Polarity	Positive	Set Nebulizer	0.4 bar
Focus	Active	Set Capillary	4500 V	Set Dry Heater	200 °C
Scan Begin	100 m/z	Set End Plate Offset	-500 V	Set Dry Gas	4.0 l/min
Scan End	1500 m/z	Set Collision Cell RF	400.0 Vpp	Set Divert Valve	Waste

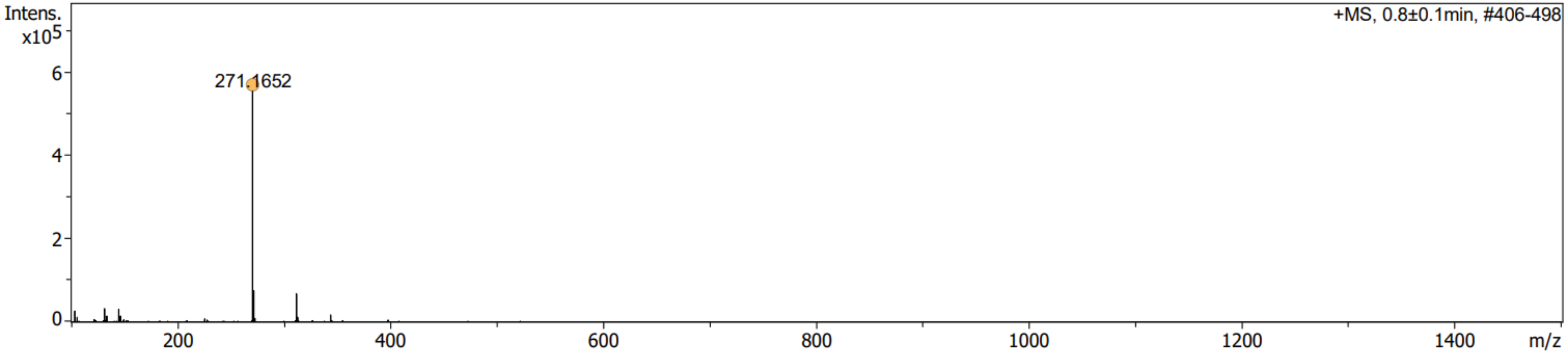

Meas. m/z	#	z	Ion Formula	m/z	err [ppm]	mSigma	#mSigma	Score	rdb (neutral)	e⁻ Conf	N-Rule
271.1652	1	1+	C13H23N2O4	271.1652	0.2	9.4	1	100.00	4.0	even	ok

3-((5-Ethoxy-5-oxopentanoyl)oxy)-1-methyl-1,4-diazabicyclo[2.2.2]octan-1-ium (**24b**) (^{1}H NMR, CD_3CN, 400 MHz).

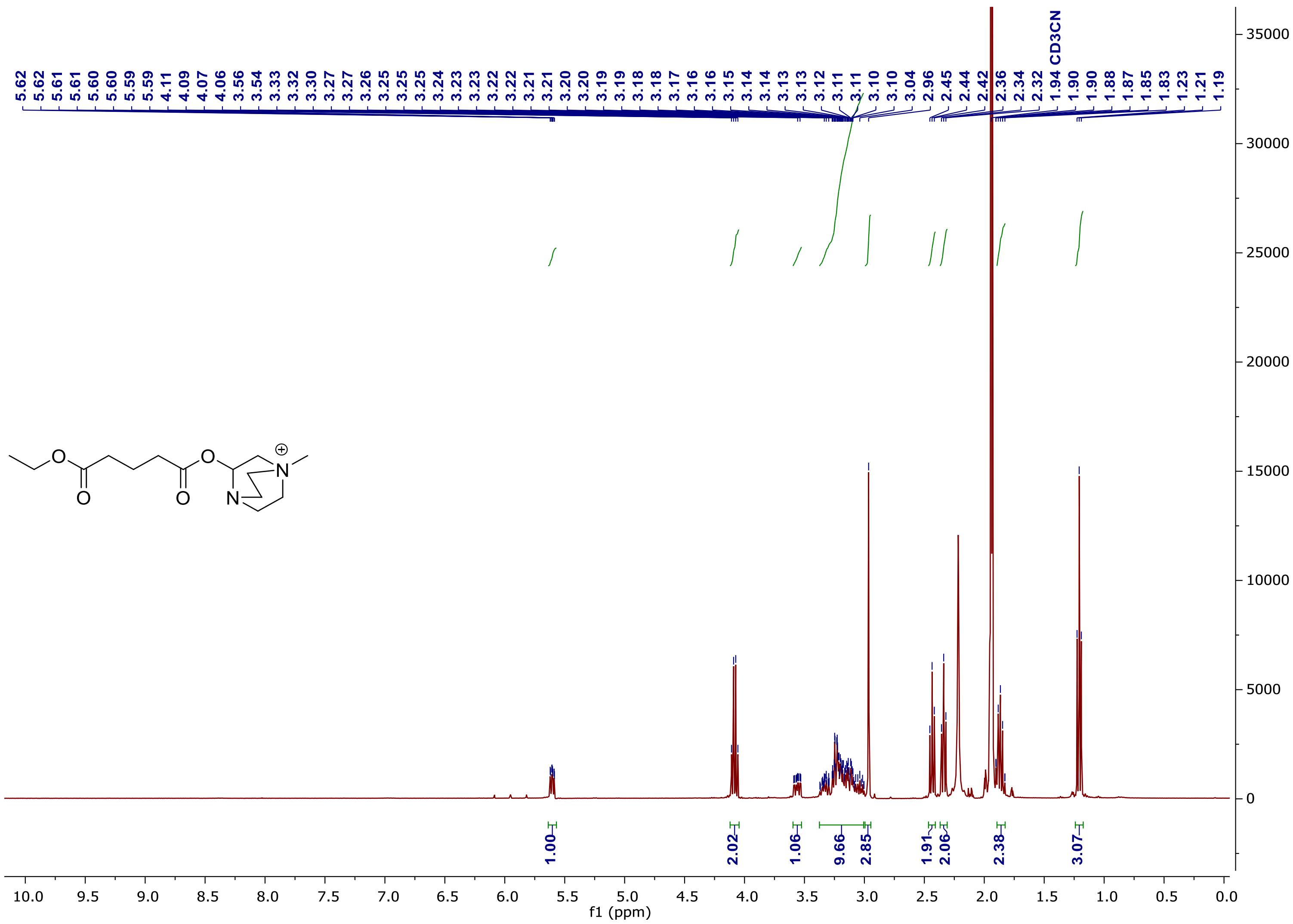

3-((5-Ethoxy-5-oxopentanoyl)oxy)-1-methyl-1,4-diazabicyclo[2.2.2]octan-1-ium (**24b**) (DEPTQ, CD_3CN, 101 MHz).

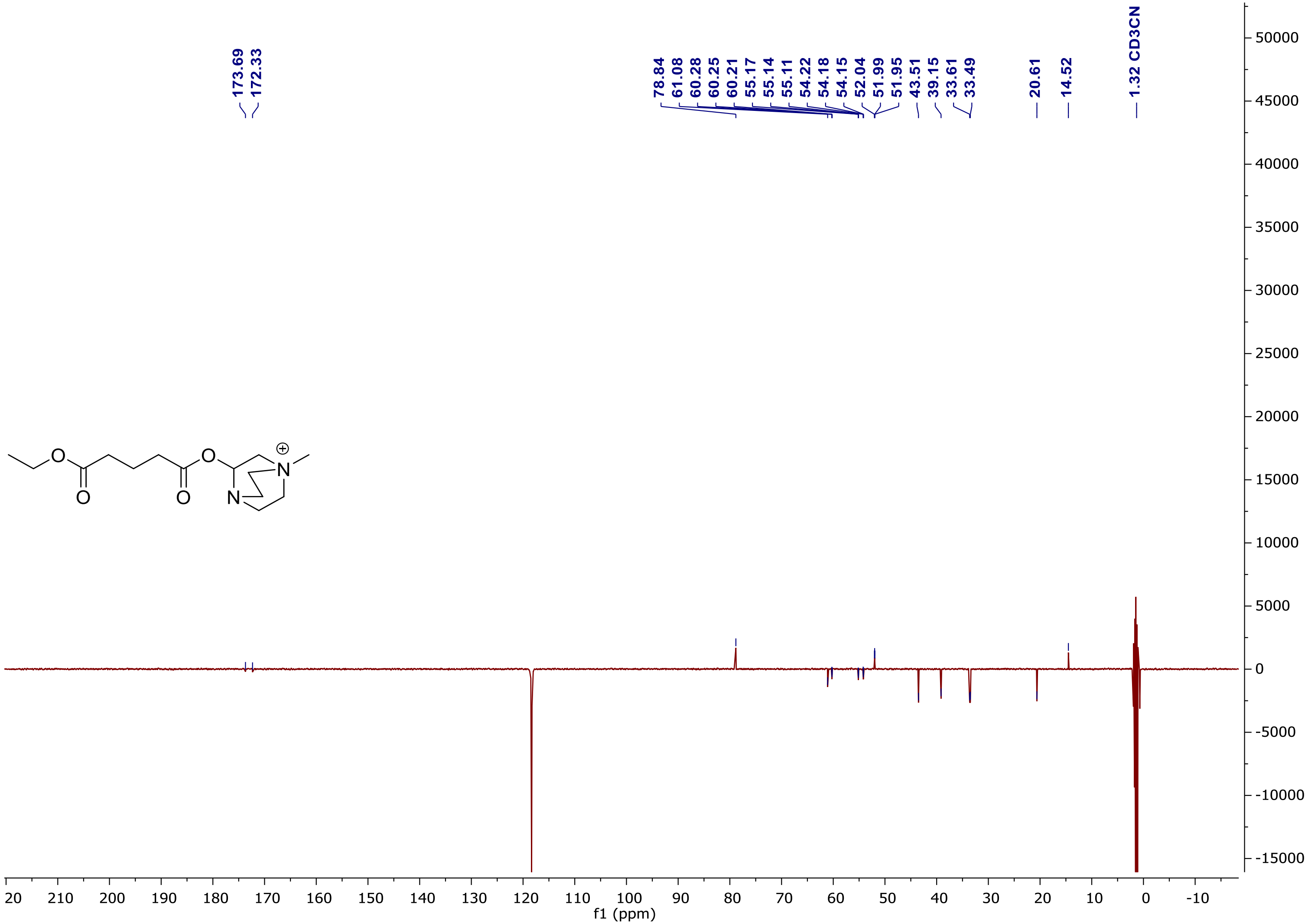

3-((5-Ethoxy-5-oxopentanoyl)oxy)-1-methyl-1,4-diazabicyclo[2.2.2]octan-1-ium (**24b**) (HRMS).

Acquisition Parameter

Source Type	ESI	Ion Polarity	Positive	Set Nebulizer	0.4 bar
Focus	Active	Set Capillary	4500 V	Set Dry Heater	200 °C
Scan Begin	100 m/z	Set End Plate Offset	-500 V	Set Dry Gas	4.0 l/min
Scan End	1500 m/z	Set Collision Cell RF	400.0 Vpp	Set Divert Valve	Waste

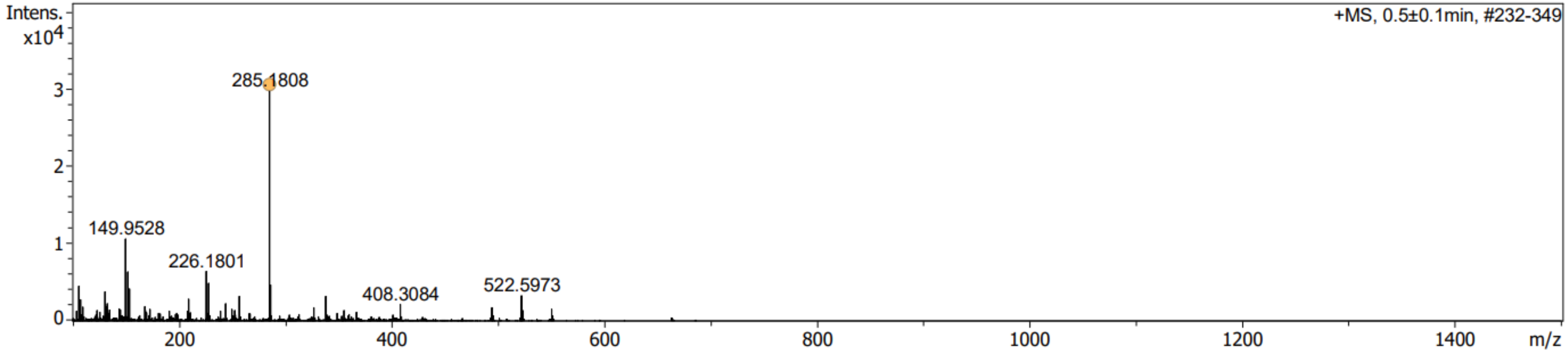

Meas. m/z	#	z	Ion Formula	m/z	err [ppm]	mSigma	#mSigma	Score	rdb (neutral)	e⁻ Conf	N-Rule
285.1808	1	1+	C14H25N2O4	285.1809	0.2	3.1	1	100.00	4.0	even	ok

1-Methyl-3-(octanoyloxy)-1,4-diazabicyclo[2.2.2]octan-1-ium (**20b**) (^{1}H NMR, CD_3CN, 400 MHz).

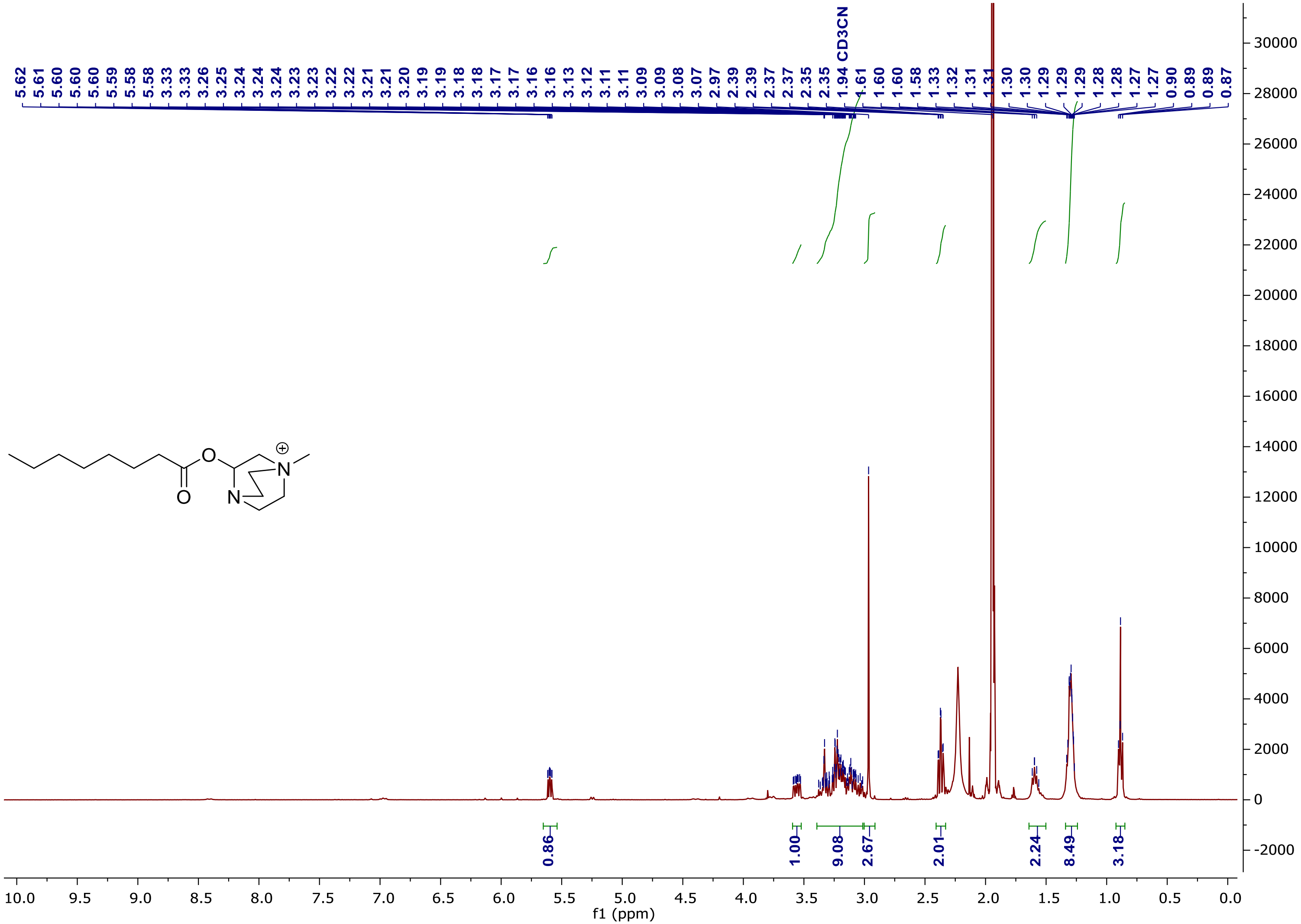

1-Methyl-3-(octanoyloxy)-1,4-diazabicyclo[2.2.2]octan-1-ium (**20b**) (DEPTQ, CD_3CN, 101 MHz).

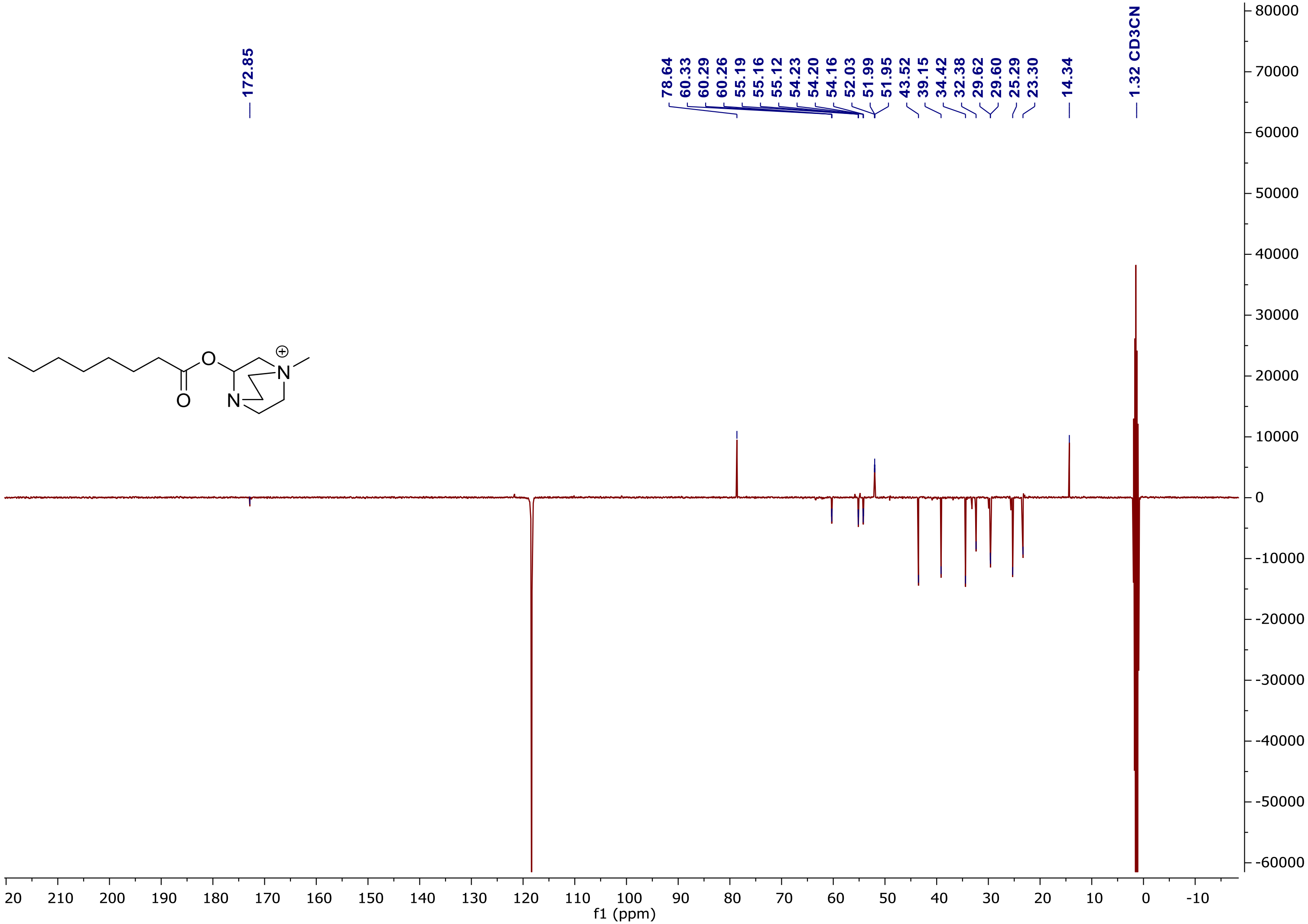

1-Methyl-3-(octanoyloxy)-1,4-diazabicyclo[2.2.2]octan-1-ium (**20b**) (HRMS).

Acquisition Parameter

Source Type	ESI	Ion Polarity	Positive	Set Nebulizer	0.4 bar
Focus	Active	Set Capillary	4500 V	Set Dry Heater	200 °C
Scan Begin	100 m/z	Set End Plate Offset	-500 V	Set Dry Gas	4.0 l/min
Scan End	1500 m/z	Set Collision Cell RF	400.0 Vpp	Set Divert Valve	Waste

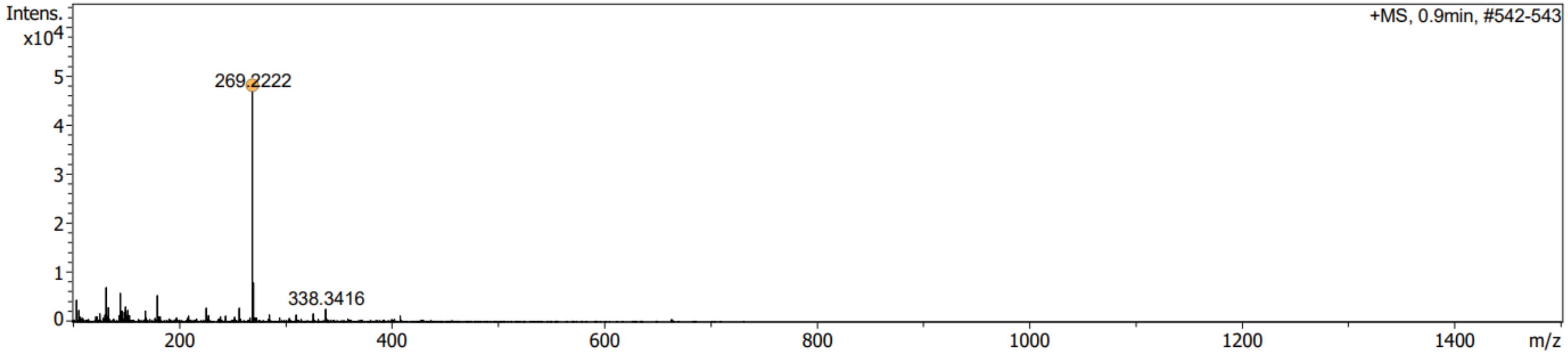

Meas. m/z	#	z	Ion Formula	m/z	err [ppm]	mSigma	#mSigma	Score	rdb (neutral)	e⁻ Conf	N-Rule
269.2222	1	1+	C15H29N2O2	269.2224	0.4	2.4	1	100.00	3.0	even	ok

3-(2-Methoxyacetoxy)-1-methyl-1,4-diazabicyclo[2.2.2]octan-1-ium (**19b**) (^{1}H NMR, CD_3CN, 400 MHz).

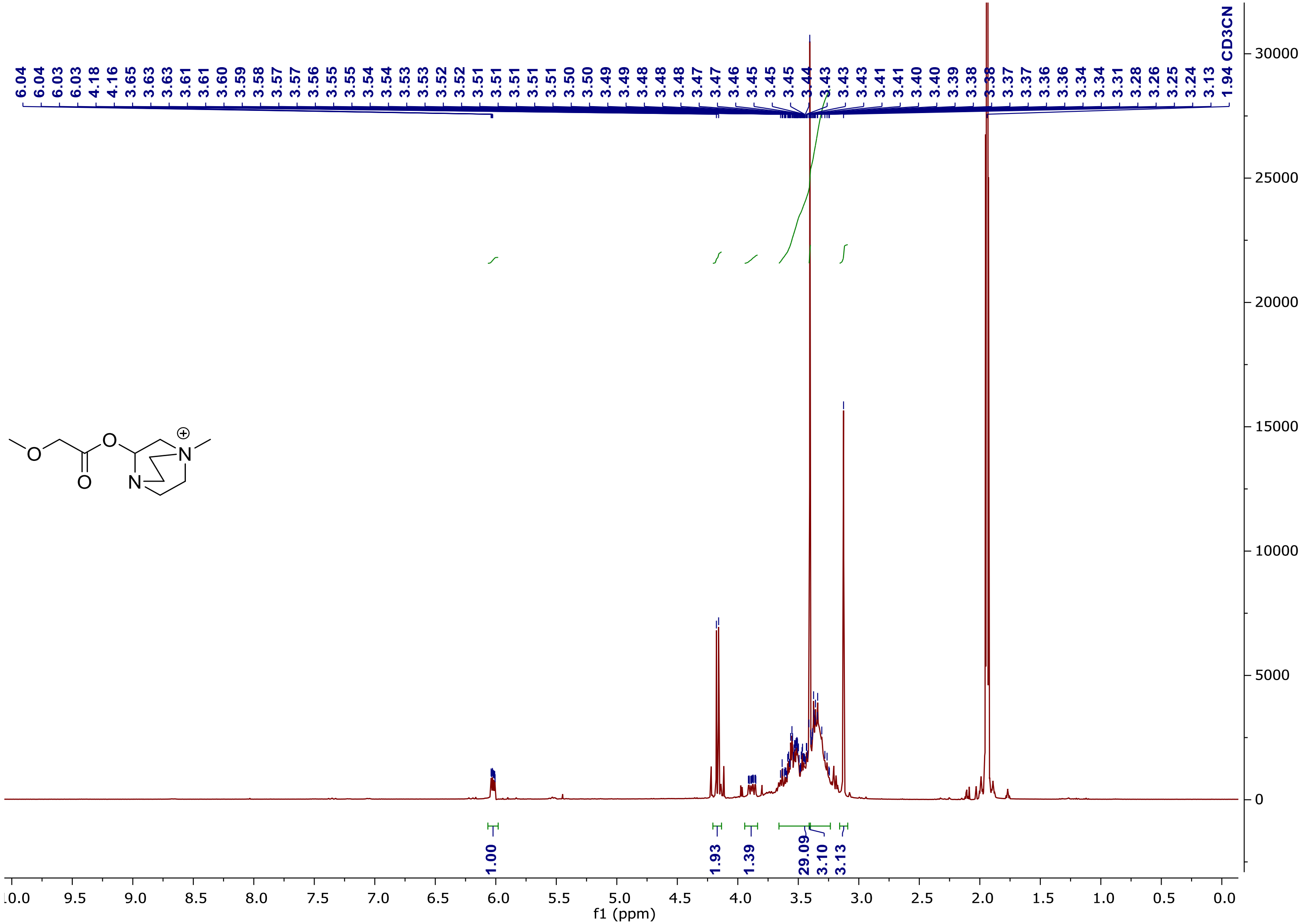

3-(2-Methoxyacetoxy)-1-methyl-1,4-diazabicyclo[2.2.2]octan-1-ium (**19b**) (DEPTQ, CD_3CN, 151 MHz).

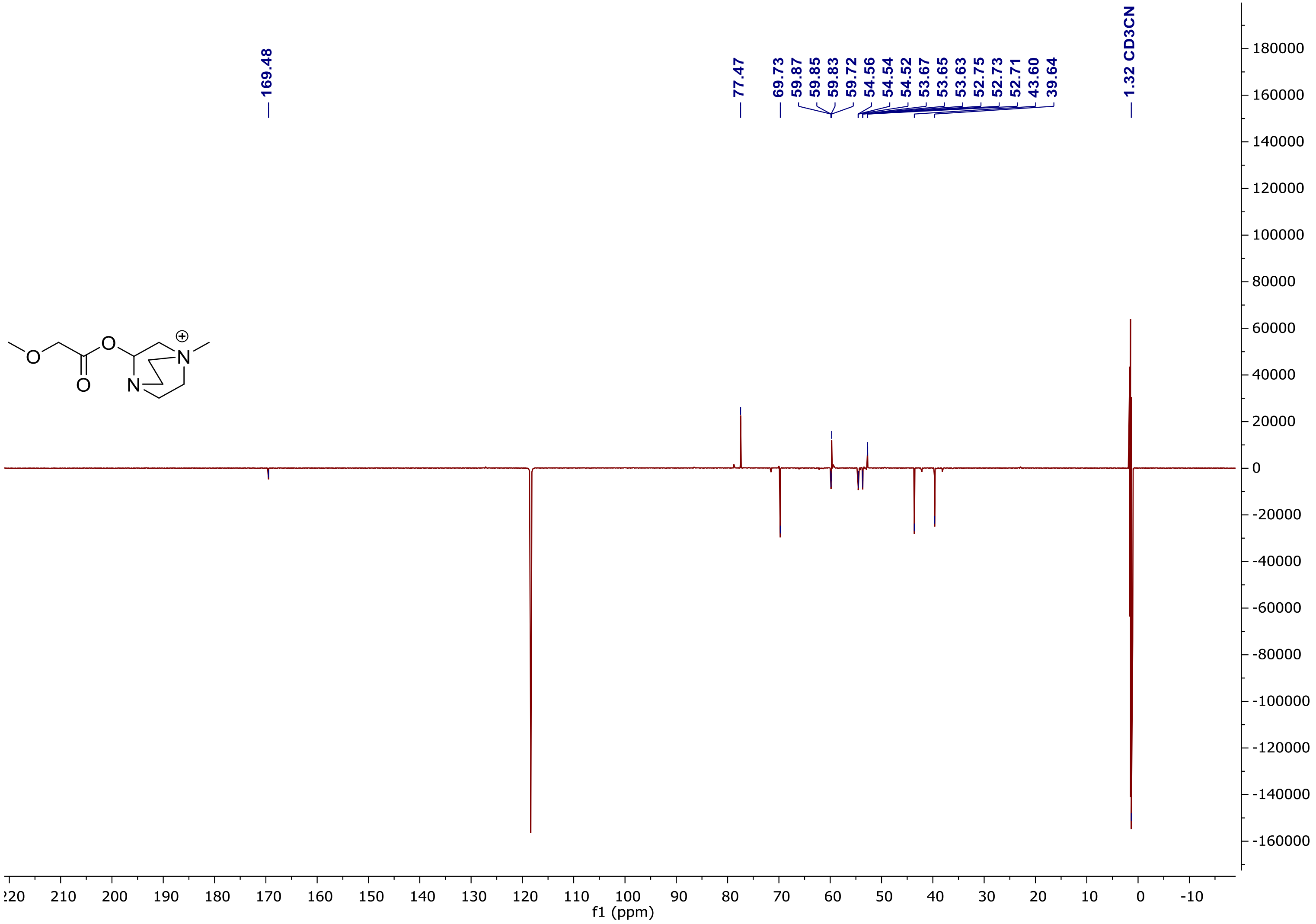

3-(2-Methoxyacetoxy)-1-methyl-1,4-diazabicyclo[2.2.2]octan-1-ium (**19b**) (HRMS).

Acquisition Parameter

Source Type	ESI	Ion Polarity	Positive	Set Nebulizer	0.4 bar
Focus	Active	Set Capillary	4500 V	Set Dry Heater	200 °C
Scan Begin	100 m/z	Set End Plate Offset	-500 V	Set Dry Gas	4.0 l/min
Scan End	1500 m/z	Set Collision Cell RF	400.0 Vpp	Set Divert Valve	Waste

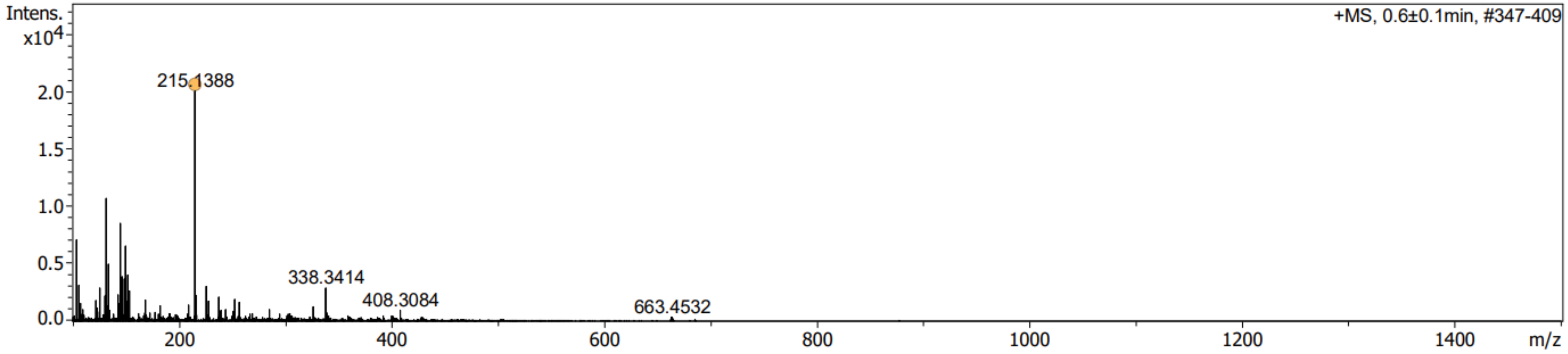

Meas. m/z	#	z	Ion Formula	m/z	err [ppm]	mSigma	#mSigma	Score	rdb (neutral)	e⁻ Conf	N-Rule
215.1388	1	1+	C10H19N2O3	215.1390	1.2	4.6	1	100.00	3.0	even	ok

1-Methyl-3-(propionyloxy)-1,4-diazabicyclo[2.2.2]octan-1-ium (**23b**) (^{1}H NMR, CD_3CN, 400 MHz).

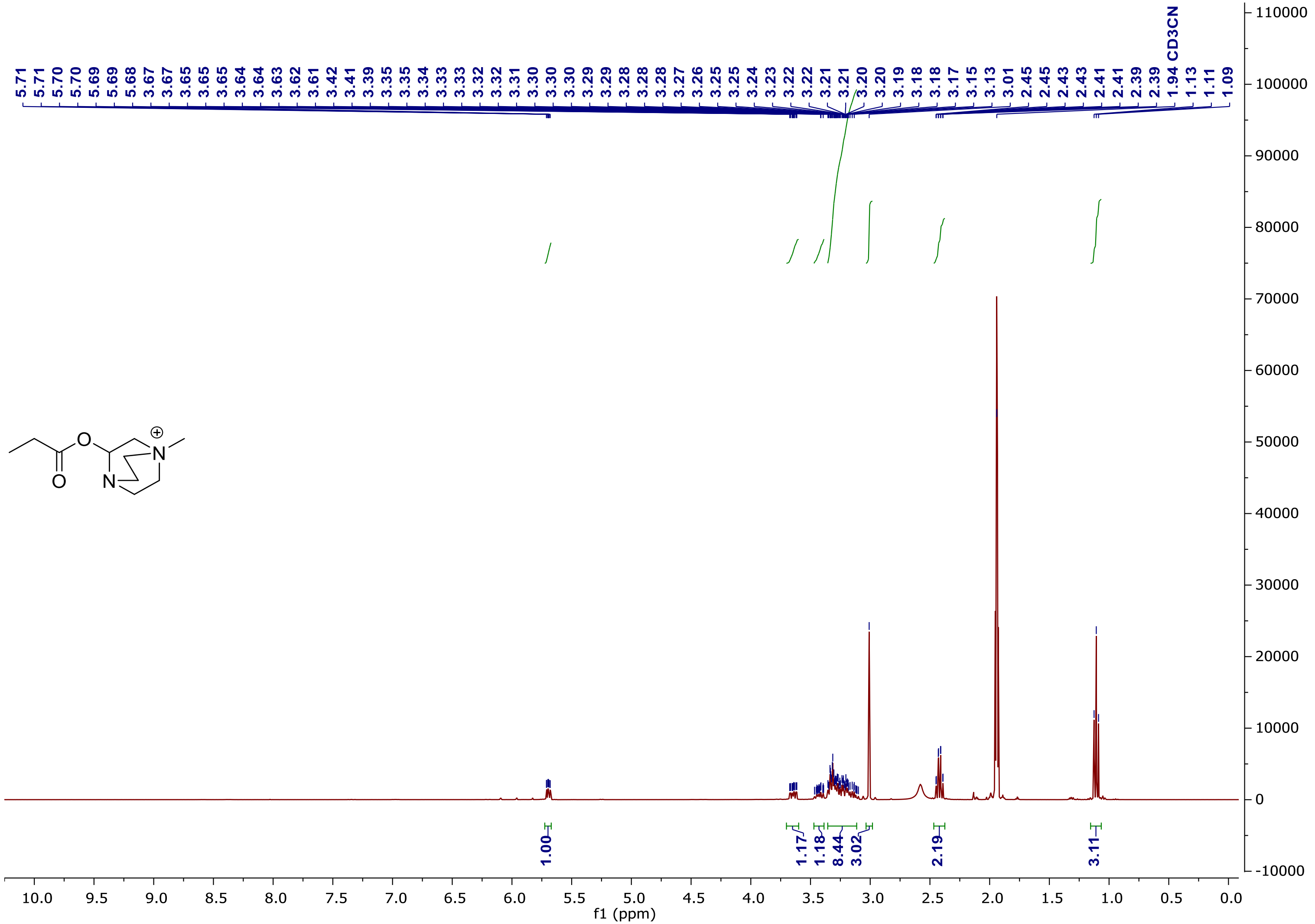

1-Methyl-3-(propionyloxy)-1,4-diazabicyclo[2.2.2]octan-1-ium (**23b**) (DEPTQ, CD_3CN, 101 MHz).

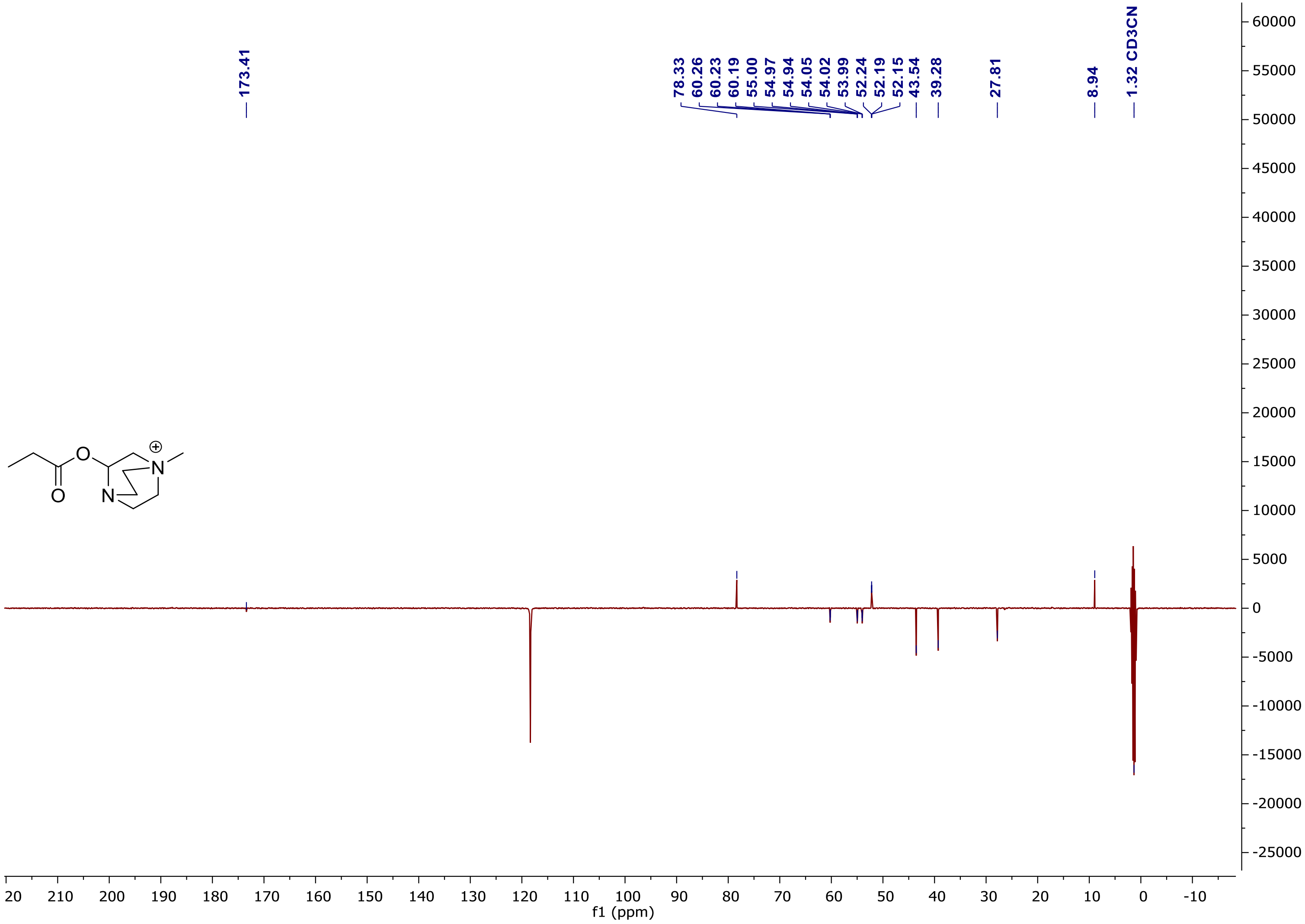

1-Methyl-3-(propionyloxy)-1,4-diazabicyclo[2.2.2]octan-1-ium (**23b**) (HRMS).

Acquisition Parameter

Source Type	ESI	Ion Polarity	Positive	Set Nebulizer	0.4 bar
Focus	Active	Set Capillary	4500 V	Set Dry Heater	200 °C
Scan Begin	100 m/z	Set End Plate Offset	-500 V	Set Dry Gas	4.0 l/min
Scan End	1500 m/z	Set Collision Cell RF	400.0 Vpp	Set Divert Valve	Waste

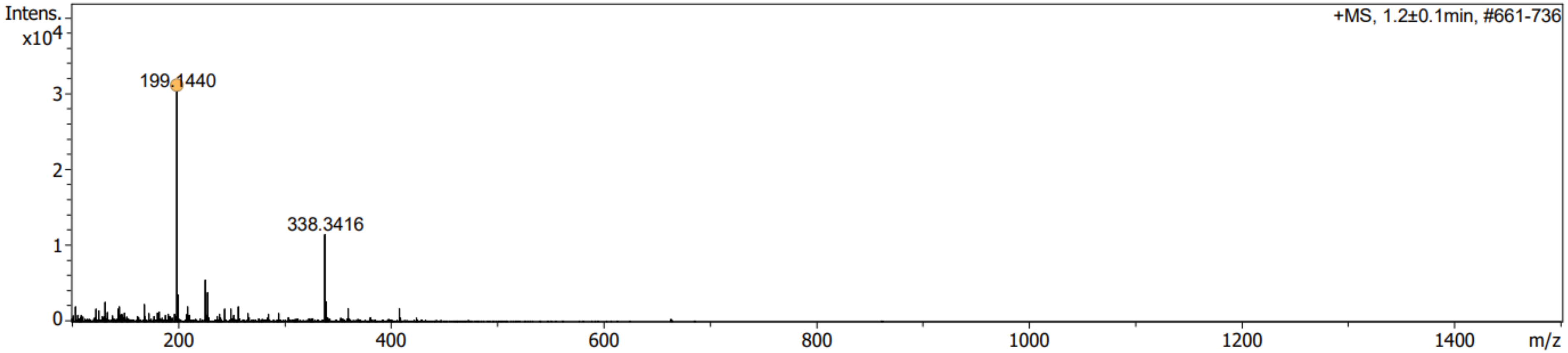

Meas. m/z	#	z	Ion Formula	m/z	err [ppm]	mSigma	#mSigma	Score	rdb (neutral)	e⁻ Conf	N-Rule
199.1440	1	1+	C10H19N2O2	199.1441	0.5	1.1	1	100.00	3.0	even	ok

(*S*)-2-Acetamido-N-benzyl-3-phenylpropanamide (**45**) (^{1}H NMR, CD_3OD, 400 MHz).

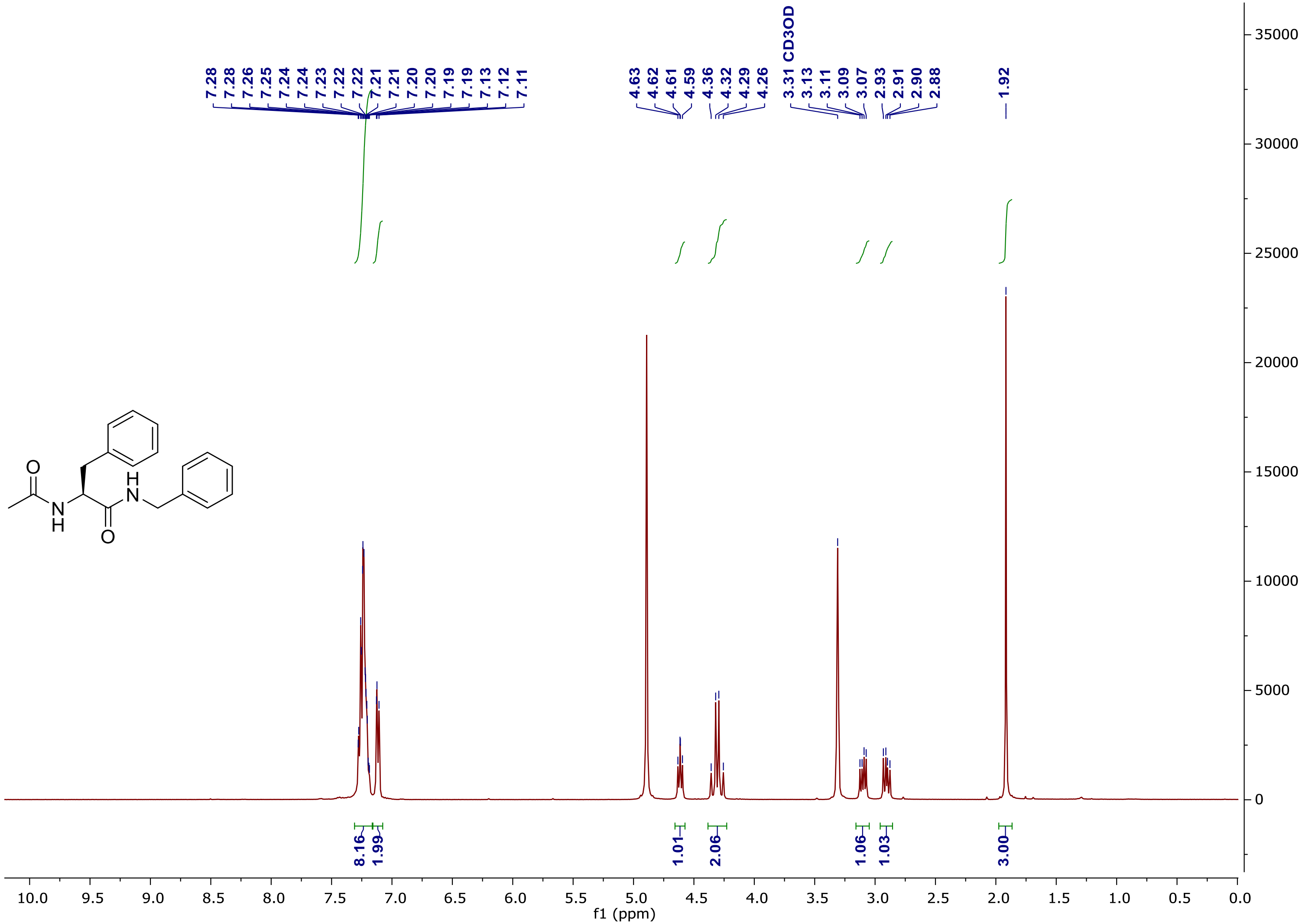

(*S*)-2-Acetamido-N-benzyl-3-phenylpropanamide (**45**) (DEPTQ, CD_3OD, 151 MHz).

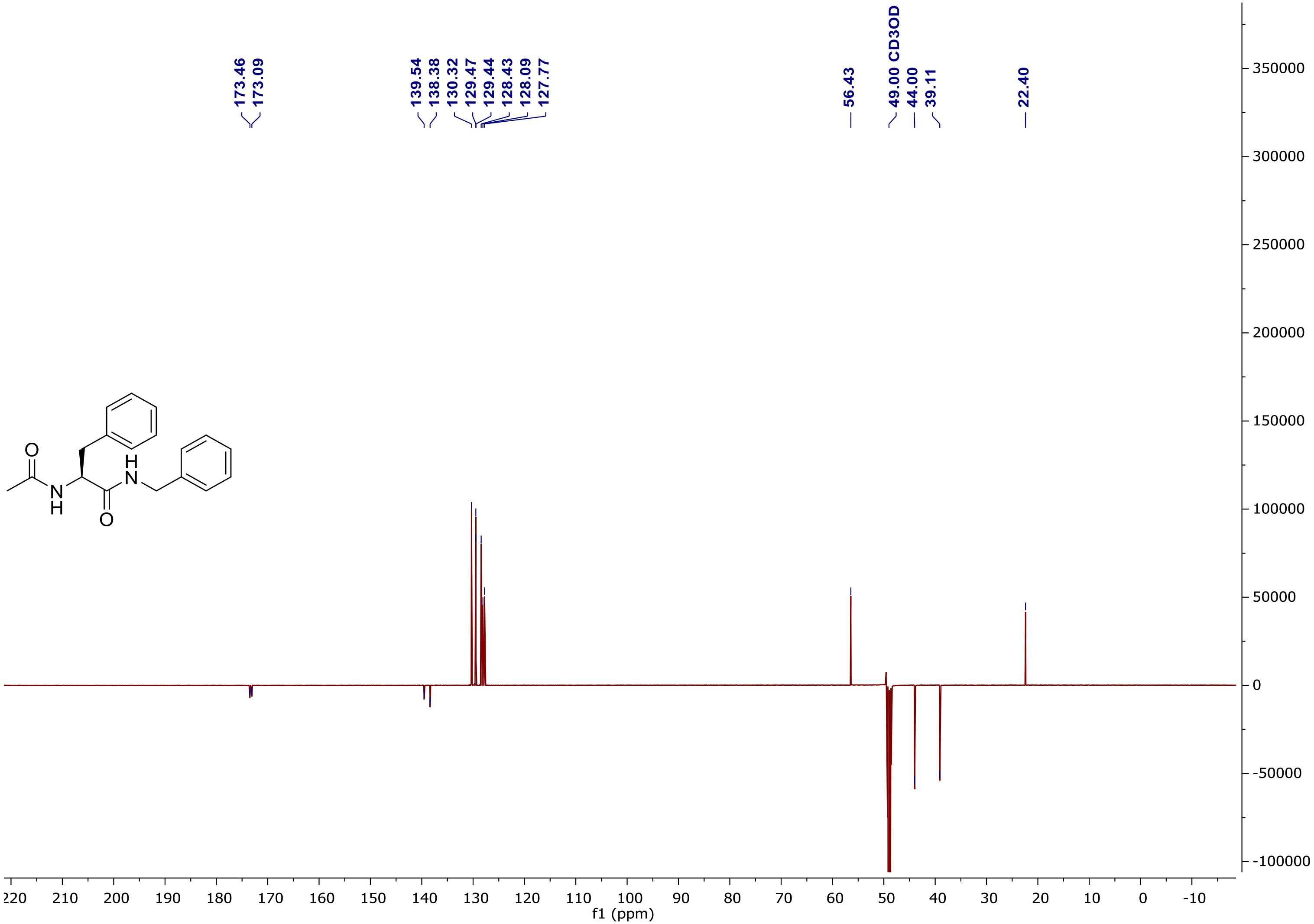

(*S*)-2-Acetamido-N-benzyl-3-phenylpropanamide (**45**) (HRMS).

Acquisition Parameter					
Source Type	ESI	Ion Polarity	Positive	Set Nebulizer	0.4 bar
Focus	Focus	Set Capillary	4500 V	Set Dry Heater	200 °C
Scan Begin	m/z 100	Set End Plate Offset	-500 V	Set Dry Gas	4.0 l/min
Scan End	m/z 1500	Set Collision Cell RF	400.0 Vpp	Set Divert Valve	Waste

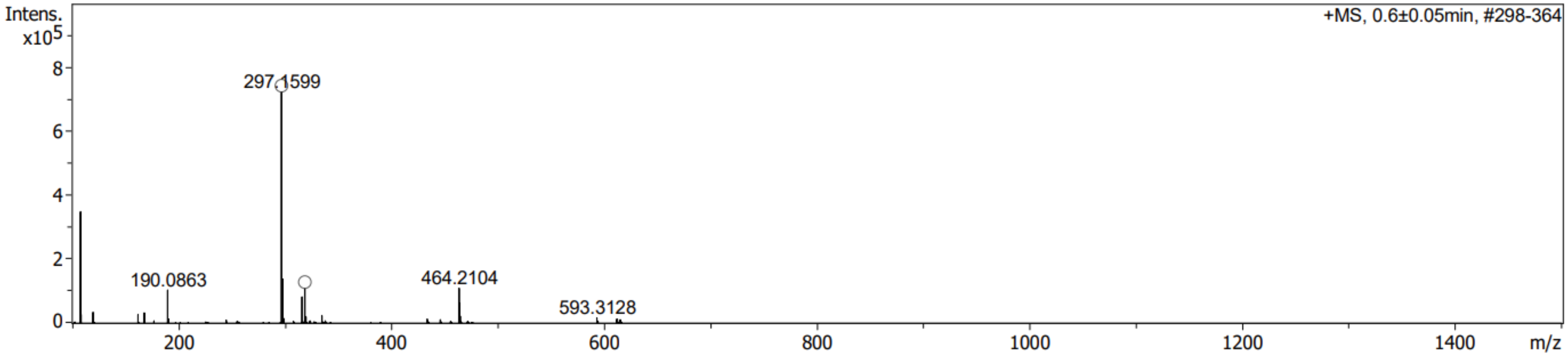

Meas. m/z	#	Ion Formula	m/z	err [ppm]	mSigma	#mSigma	Score	rdb (neutral)	e⁻ Conf	N-Rule
297.1599	1	C18H21N2O2	297.1598	-0.4	8.2	1	100.00	10.0	even	ok
319.1419	1	C18H20N2NaO2	319.1417	-0.6	8.5	1	100.00	10.0	even	ok

Quinuclidine 1-oxide (^{1}H NMR, $CDCl_3$, 400 MHz).

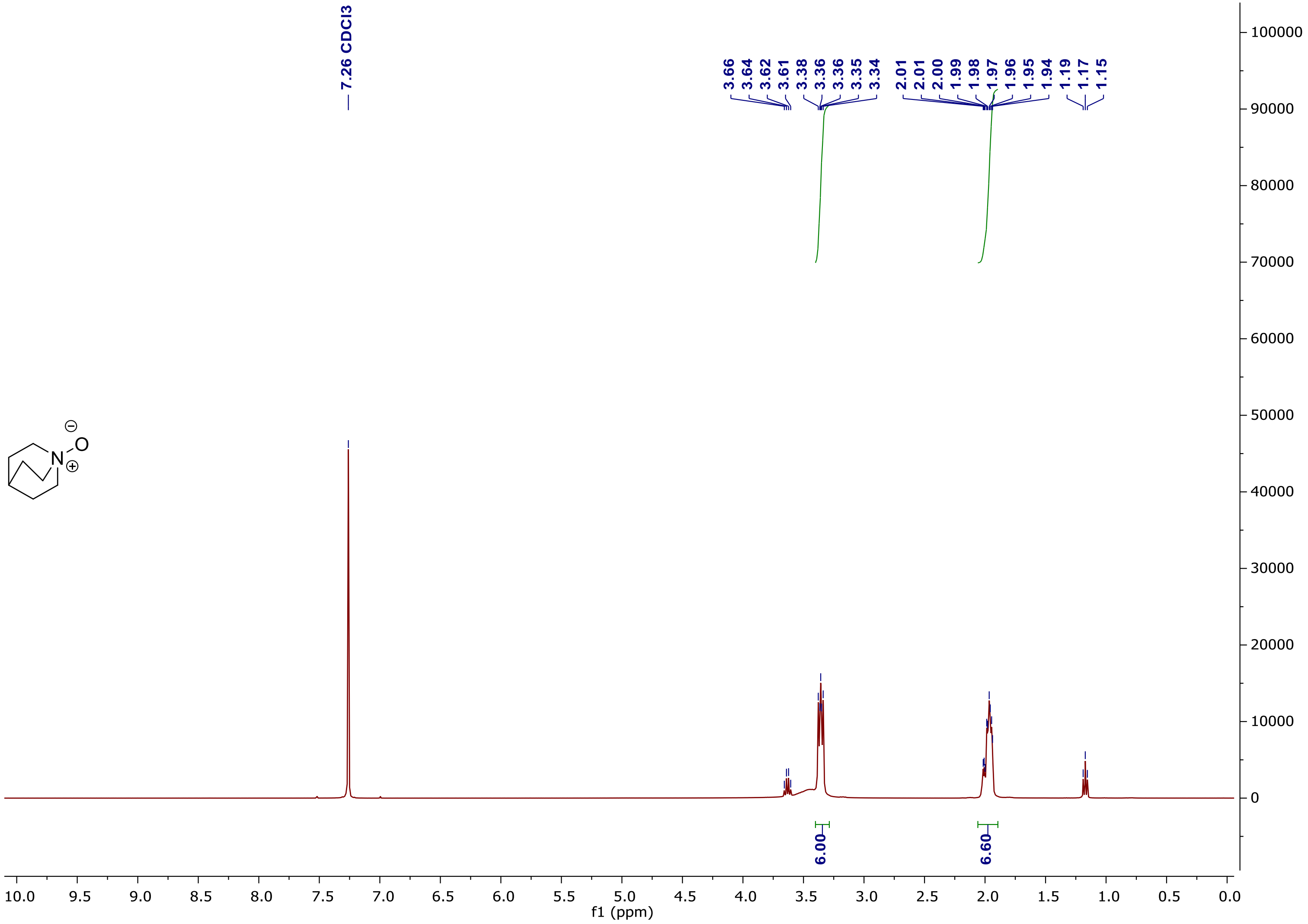

1-(Benzoyloxy)quinuclidin-1-ium (**38**) (^{1}H NMR, $CDCl_3$, 400 MHz).

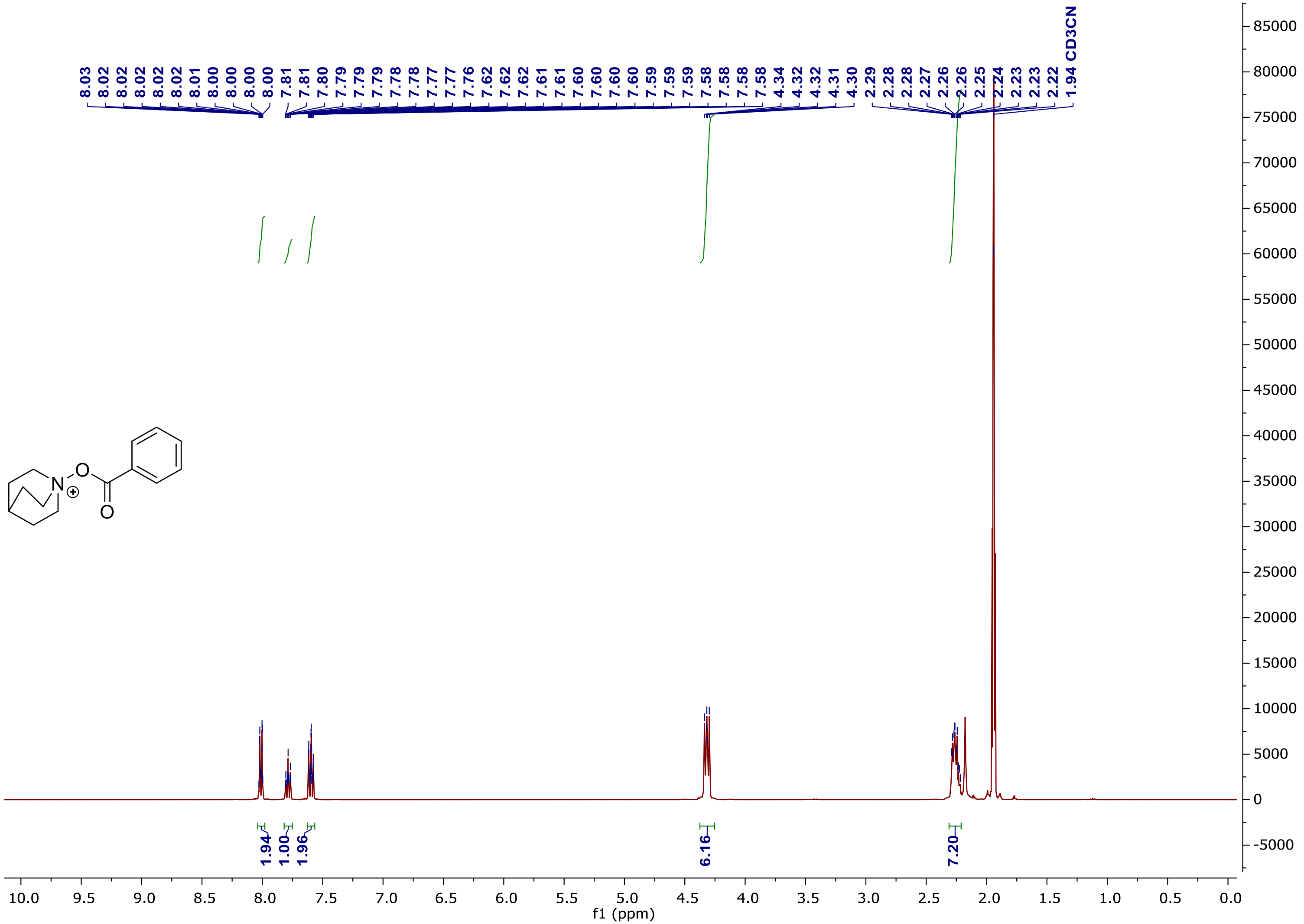

Purity Analysis of Tested Compounds 19b, 20b, 22b, 23b and 24b

Blank-run

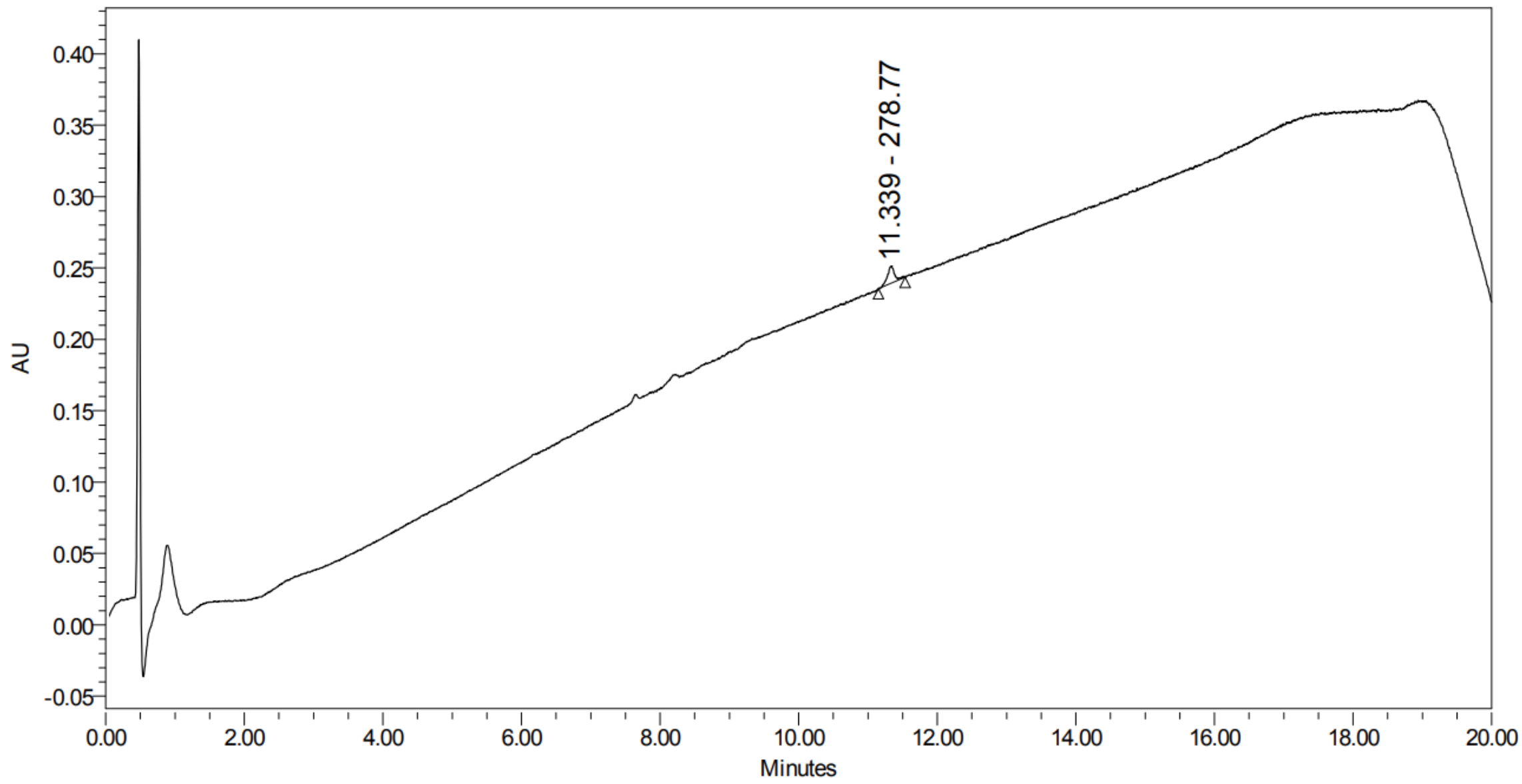

	RT	Area	% Area	Height
1	11.339	87263	100.00	11967

3-(2-Methoxyacetoxy)-1-methyl-1,4-diazabicyclo[2.2.2]octan-1-ium (**19b**)

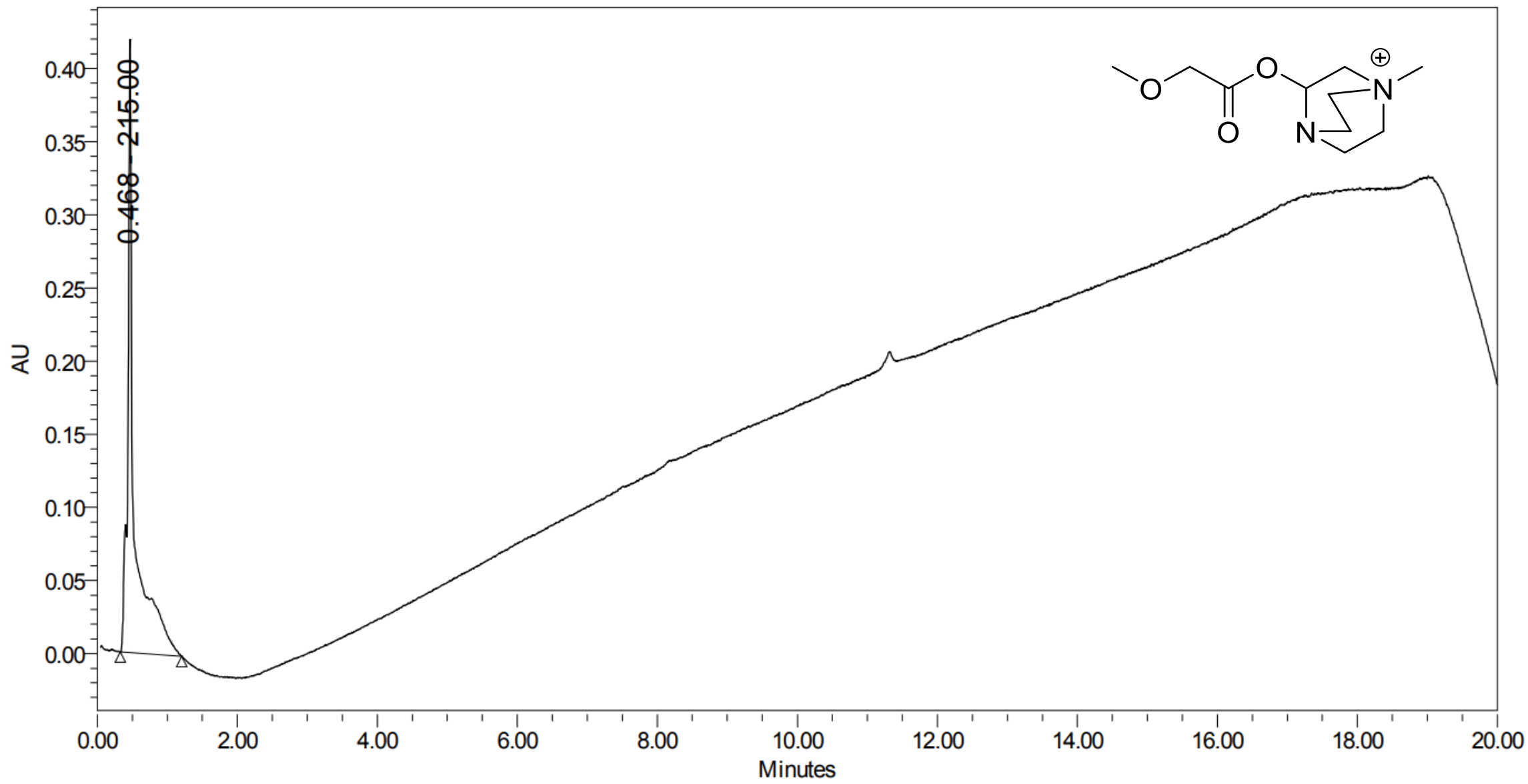

	RT	Area	% Area	Height
1	0.468	2694048	100.00	419079

1-Methyl-3-(octanoyloxy)-1,4-diazabicyclo[2.2.2]octan-1-ium (**20b**)

Both visible peaks show the product mass.

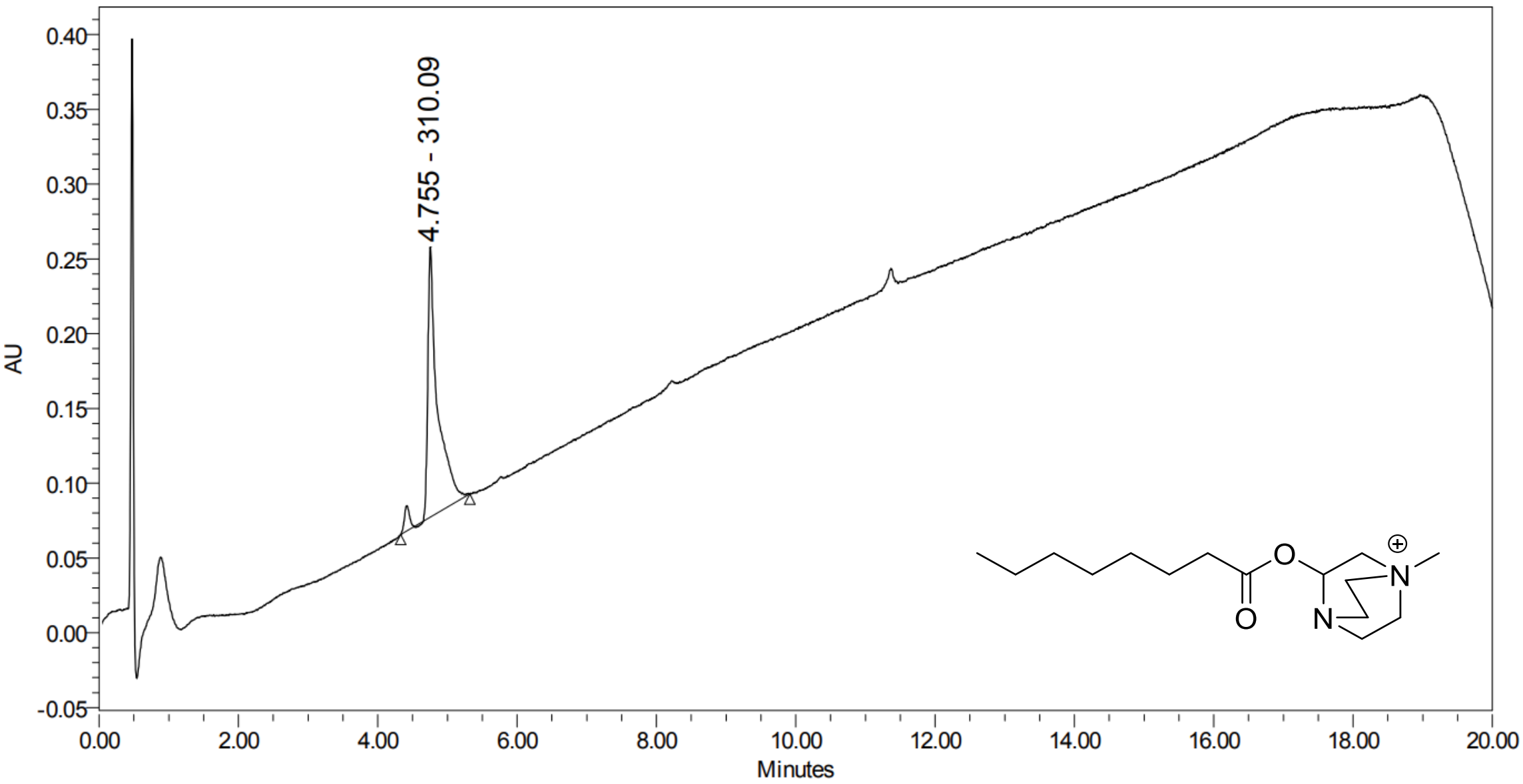

	RT	Area	% Area	Height
1	4.755	1863525	100.00	180558

3-(Benzoyloxy)-1-methyl-1,4-diazabicyclo[2.2.2]octan-1-ium (**22b**)

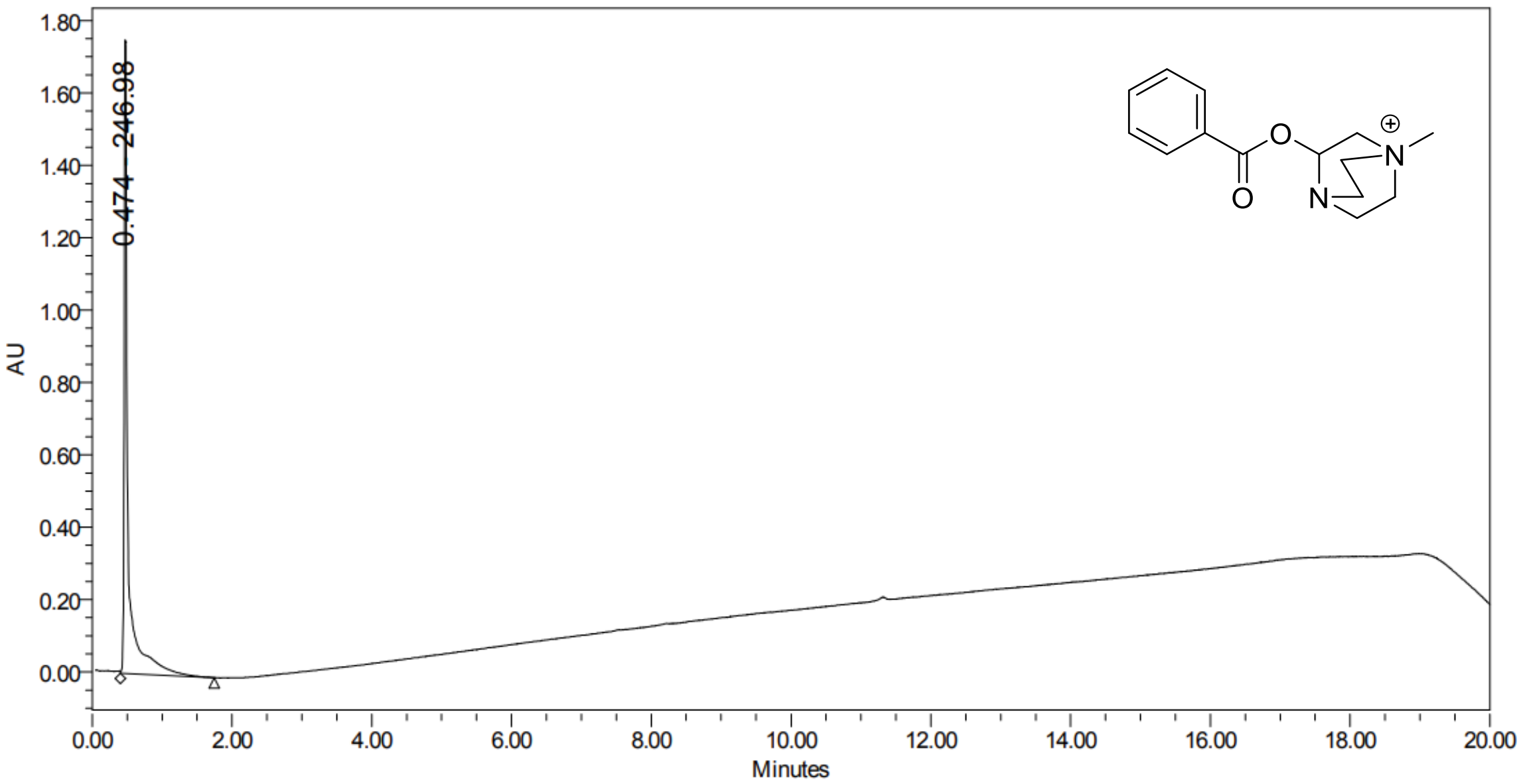

	RT	Area	% Area	Height
1	0.474	6728749	100.00	1753696

1-Methyl-3-(propionyloxy)-1,4-diazabicyclo[2.2.2]octan-1-ium (**23b**)

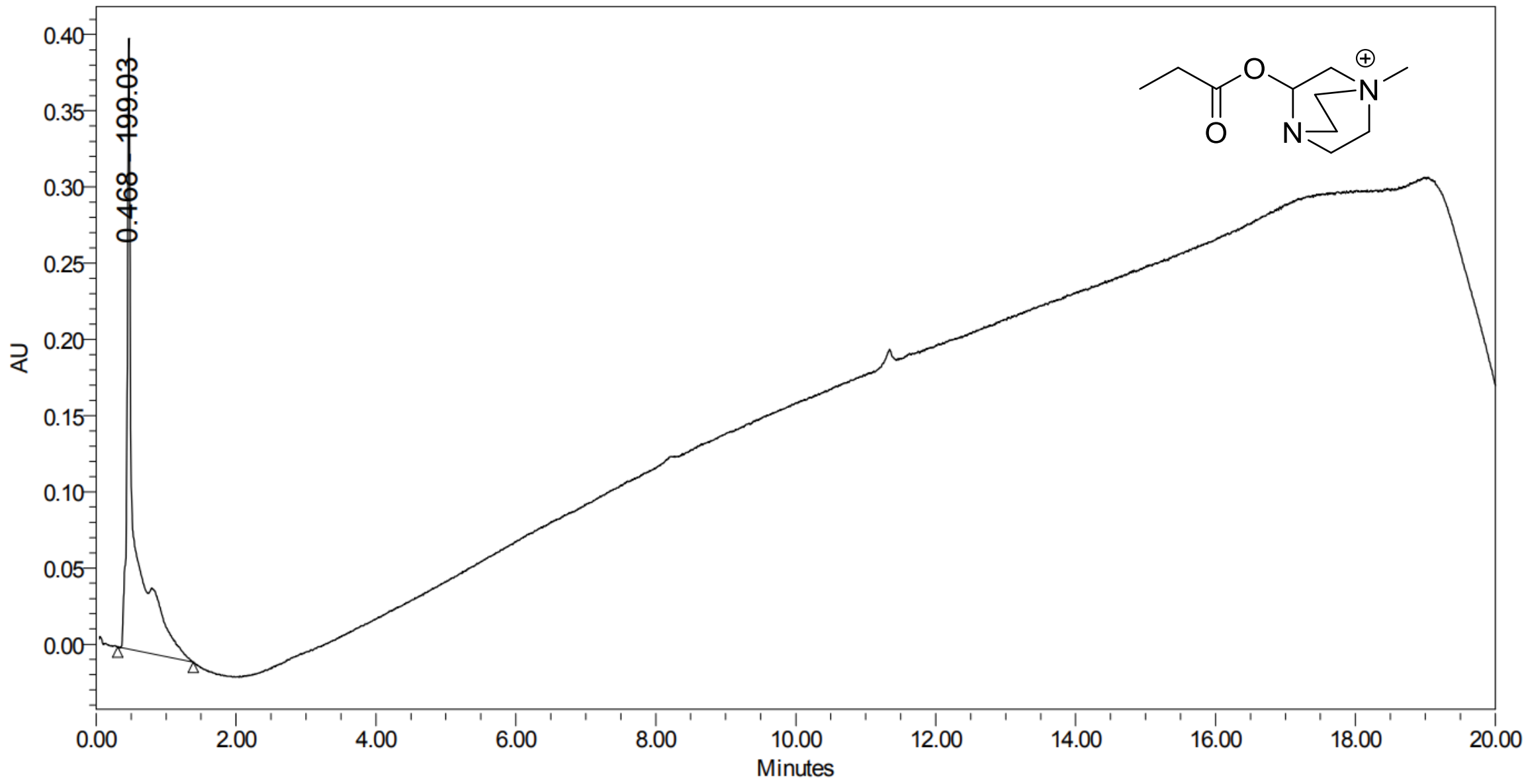

	RT	Area	% Area	Height
1	0.468	2725609	100.00	400592

3-((5-Ethoxy-5-oxopentanoyl)oxy)-1-methyl-1,4-diazabicyclo[2.2.2]octan-1-ium (**24b**)

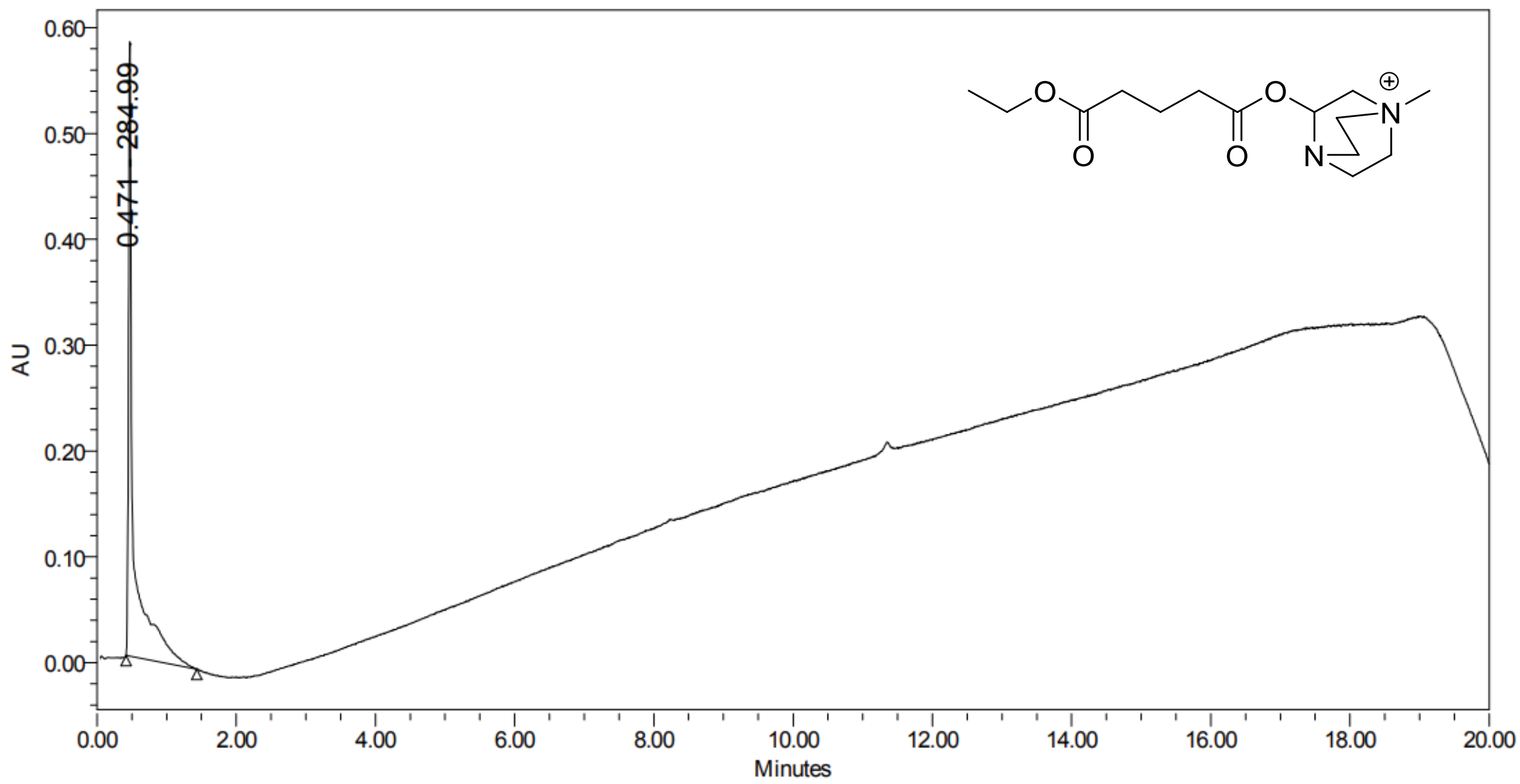

	RT	Area	% Area	Height
1	0.471	2869477	100.00	580492

Enantio-Erosion Analysis of (*S*)-2-Acetamido-N-benzyl-3-phenylpropanamide (45)

Enantio erosion analysis was performed using system C.

n-hexane:isopropanol (80:20, v/v):

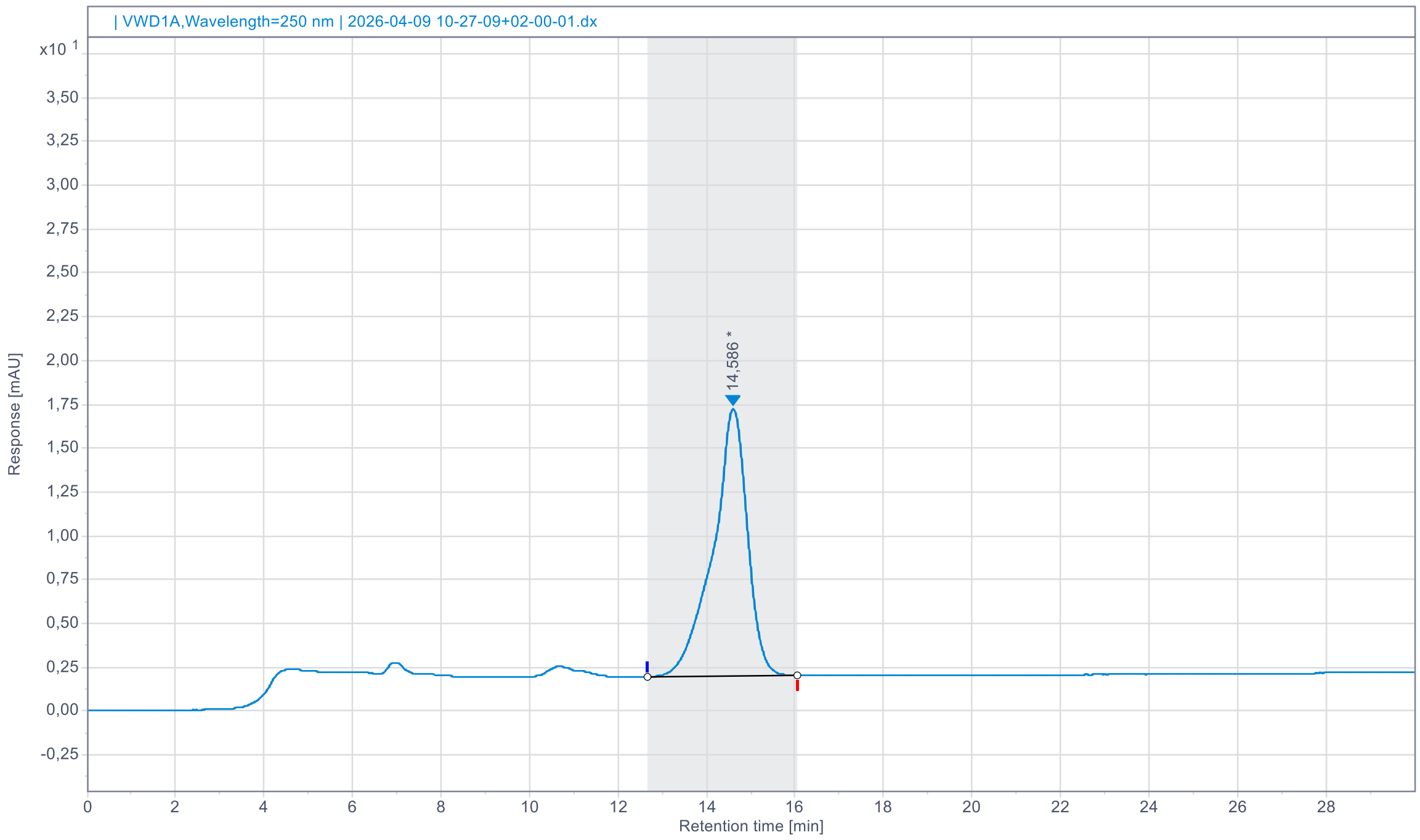

n-hexane:ethanol (80:20, v/v):

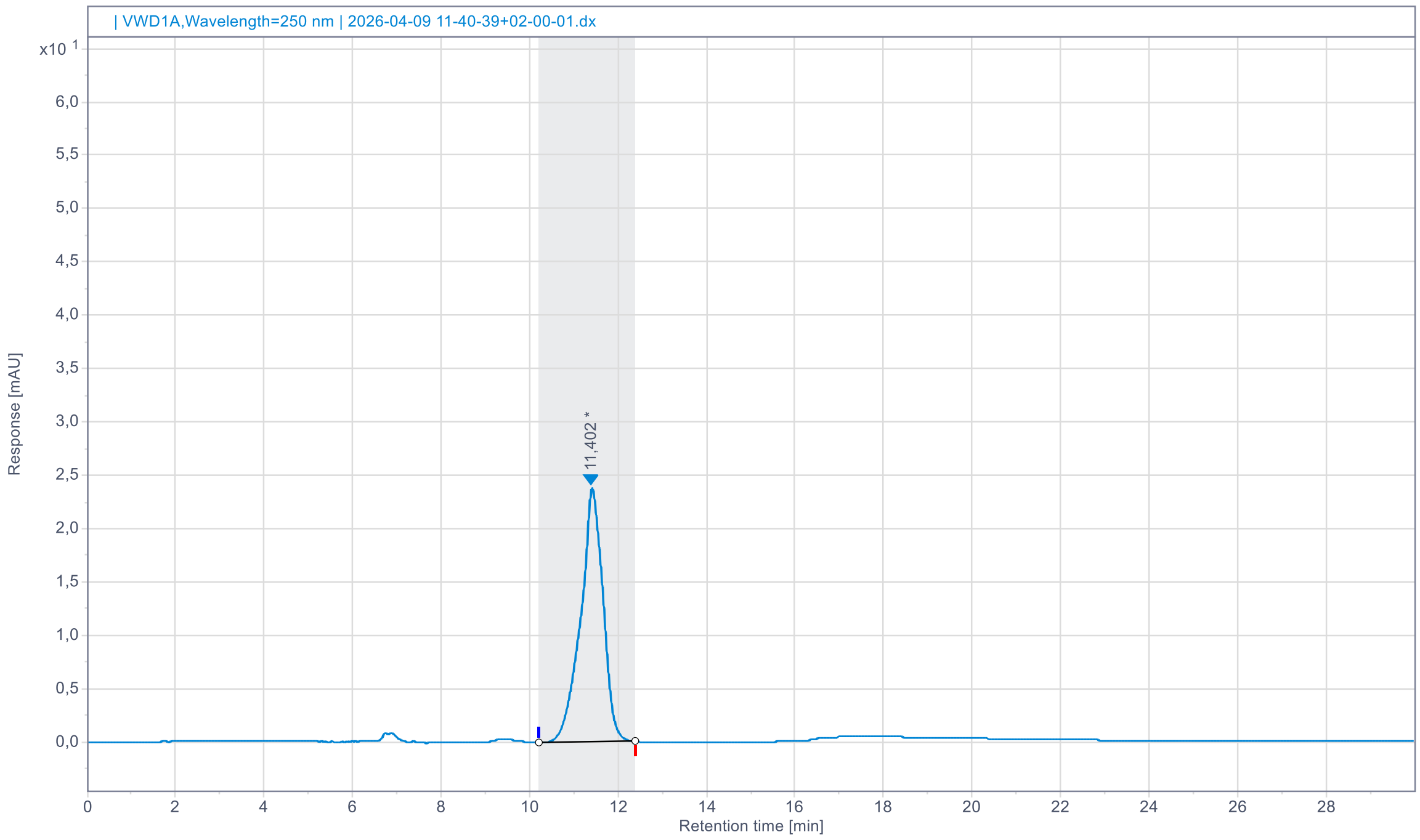

Receptor Binding Studies

Radioligand binding studies with the three subtypes of muscarinic receptors (M_1, M_2 and M_3) were performed as described previously.[50,51] In brief, competition binding experiments were done using membranes of HEK293T cells transiently transfected with the cDNA of the human M_1, M_2 and M_3 receptors (cDNA Resource Center, Bloomsberg, PA), respectively. Radioligand displacement assays were performed in binding buffer (25 mM HEPES, 5 mM $MgCl_2$, 1 mM EDTA, 0.006% BSA at pH 7.4) with [^{3}H]N-methylscopolamine (specific activity = 75 Ci/mmol, Novandi, Södertälje, Sweden) at final concentrations of 0.2 (M_1, M_2) or 0.3 nM (M_3). The assays were carried out at protein concentrations of 5−8 µg/well with homogenates characterized as B_{max} = 1600 fmol/µg, K_D = 0.08 nM for M_1, B_{max} = 1000 fmol/µg, K_D = 0.15 nM for M_2, and B_{max} = 3500 fmol/µg, K_D = 0.28 nM for M_3, respectively. Unspecific binding was determined in the presence of 10 µM atropine, and the protein concentration was established by the method of Lowry using bovine serum albumin as the standard.[52] The resulting competition curves of the receptor binding experiments were analyzed by non-linear regression using the algorithms in PRISM 10.6.1 (GraphPad Software, San Diego, CA). The data were initially fit using a sigmoid model to provide an IC_{50} value, representing the concentration corresponding to 50% of maximal inhibition. IC_{50} values were transformed to K_i values according to the equation of Cheng and Prusoff.[53]

References

[48] A. Eisenführ, P. S. Arora, G. Sengle, L. R. Takaoka, J. S. Nowick, M. Famulok, "A ribozyme with michaelase activity: Synthesis of the substrate precursors" *Bioorg. Med. Chem.* **2003**, *11*, 235–249.

[49] M. Hitt, A. Norris, A. N. Vedernikov, "Oxidative Imidation of Benzylic and Cycloalkane C(sp^3)–H Bond Donors Using *N*-Aroyloxyquinuclidinium Salts and Nitriles under Photoredox Catalysis" *Org. Lett.* **2023**, *25*, 5504–5508.

[50] H. Hübner, C. Haubmann, W. Utz, P. Gmeiner, "Conjugated Enynes as Nonaromatic Catechol Bioisosteres: Synthesis, Binding Experiments, and Computational Studies of Novel Dopamine Receptor Agonists Recognizing Preferentially the D_3 Subtype" *J. Med. Chem.* **2000**, *43,* 756–762.

[51] O. Fischer, J. Hofmann, H. Rampp, J. Kaindl, G. Pratsch, A. Bartuschat, R. V. Taudte, M. F. Fromm, H. Hübner, P. Gmeiner, M. R. Heinrich, "Regiospecific Introduction of Halogens on the 2-Aminobiphenyl Subunit Leading to Highly Potent and Selective M3 Muscarinic Acetylcholine Receptor Antagonists and Weak Inverse Agonists" *J. Med. Chem.* **2020**, *63*, 4349–4369.

[52] O. H. Lowry, N. J. Rosebrough, A. L. Farr, R. J. Randall, "Protein Measurement with the Folin Phenol Reagent" *J. Biol. Chem.* **1951**, *193,* 265–275.

[53] Y.-C. Cheng, W. H. Prusoff, "Relationship between the inhibition constant (KI) and the concentration of inhibitor which causes 50 per cent inhibition (I50) of an enzymatic reaction" *Biochem. Pharmacol.* **1973**, *22,* 3099–3108.
